# What implementation interventions increase cancer screening rates? a systematic review

**DOI:** 10.1186/1748-5908-6-111

**Published:** 2011-09-29

**Authors:** Melissa C Brouwers, Carol De Vito, Lavannya Bahirathan, Angela Carol, June C Carroll, Michelle Cotterchio, Maureen Dobbins, Barbara Lent, Cheryl Levitt, Nancy Lewis, S Elizabeth McGregor, Lawrence Paszat, Carol Rand, Nadine Wathen

**Affiliations:** 1 Program in Evidence-based Care, Cancer Care Ontario, Hamilton, Ontario, Canada; 2 Departments of Oncology and Clinical Epidemiology and Biostatistics, McMaster University, Hamilton, Ontario, Canada; 3 Hamilton Urban Core Community Centre, Hamilton, Ontario, Canada; 4 Department of Family and Community Medicine, Mount Sinai Hospital, University of Toronto, Toronto, Ontario, Canada; 5 Population Studies and Surveillance, Cancer Care Ontario, Toronto, Ontario, Canada; 6 School of Nursing, McMaster University, Hamilton, Ontario, Canada; 7 Department of Family Medicine, The University of Western Ontario, London, Ontario, Canada; 8 Department of Family Medicine, McMaster University, Hamilton, Ontario, Canada; 9 Primary Care, Cancer Care Ontario, Toronto, Ontario, Canada; 10 Prevention and Screening, Cancer Care Ontario, Toronto, Ontario, Canada; 11 Population Health Research, Alberta Health Services - Cancer Epidemiology, Prevention and Screening, Calgary, Alberta, Canada; 12 Department of Health Policy Management and Evaluation, University of Toronto, Toronto, Ontario, Canada; 13 Department of Radiation Oncology, University of Toronto, Toronto, Ontario, Canada; 14 Regional Cancer Prevention and Early Detection Network Hamilton, Niagara, Haldimand, Brant, Ontario, Canada; 15 Systemic, Supportive and Regional Cancer Programs, Juravinski Cancer Centre, Hamilton, Ontario, Canada; 16 Faculty of Information and Media Studies, The University of Western Ontario, London, Ontario, Canada

## Abstract

**Background:**

Appropriate screening may reduce the mortality and morbidity of colorectal, breast, and cervical cancers. However, effective implementation strategies are warranted if the full benefits of screening are to be realized. As part of a larger agenda to create an implementation guideline, we conducted a systematic review to evaluate interventions designed to increase the rate of breast, cervical, and colorectal cancer (CRC) screening. The interventions considered were: client reminders, client incentives, mass media, small media, group education, one-on-one education, reduction in structural barriers, reduction in out-of-pocket costs, provider assessment and feedback interventions, and provider incentives. Our primary outcome, screening completion, was calculated as the overall median post-intervention absolute percentage point (PP) change in completed screening tests.

**Methods:**

Our first step was to conduct an iterative scoping review in the research area. This yielded three relevant high-quality systematic reviews. Serving as our evidentiary foundation, we conducted a formal update. Randomized controlled trials and cluster randomized controlled trials, published between 2004 and 2010, were searched in MEDLINE, EMBASE and PSYCHinfo.

**Results:**

The update yielded 66 studies new eligible studies with 74 comparisons. The new studies ranged considerably in quality. Client reminders, small media, and provider audit and feedback appear to be effective interventions to increase the uptake of screening for three cancers. One-on-one education and reduction of structural barriers also appears effective, but their roles with CRC and cervical screening, respectively, are less established. More study is required to assess client incentives, mass media, group education, reduction of out-of-pocket costs, and provider incentive interventions.

**Conclusion:**

The new evidence generally aligns with the evidence and conclusions from the original systematic reviews. This review served as the evidentiary foundation for an implementation guideline. Poor reporting, lack of precision and consistency in defining operational elements, and insufficient consideration of context and differences among populations are areas for additional research.

## Introduction

According to the World Health Organization [1], cancer is a leading cause of death worldwide, accounting for 7.6 million deaths (or 13%) in 2008. In Canada, for example, an estimated 76,200 individuals will die of cancer and 173,800 new cases will be diagnosed in 2010 [2]. Colorectal cancer (CRC) is the second highest cause of cancer death overall in Canada with an estimated 22,500 new diagnoses and 9100 deaths attributable to the disease. An estimated 23,300 women will be diagnosed with breast cancer, and 5,400 will die. For both of these diseases, early screening leading to early detection has an impact on mortality and morbidity [2]. Similarly, evidence demonstrates that cervical cancer incidence rates have been declining, a situation for the most part due to adherence to Pap test screening [2].

Given the incidence of these cancers, national and regional governments have made a commitment to increase screening rates and facilitate the early diagnosis of disease. For example, in Ontario, Canada, formal province-wide screening programs are in place for breast cancer, cervical cancer, and CRC [3]. Several clinical practice guidelines have been developed to facilitate high-quality screening [*e.g*., [4,5]]. These guidelines focus on clinical issues (*e.g*., what are the most appropriate screening manoeuvres available, and how to ensure screening is safe, valid, and reliable). However, as with any new health intervention or technology, the uptake and application of clinical recommendations is complex, variable, and at less than optimum rates [6]. Effective strategies to improve the uptake of cancer screening are warranted if the full benefits of screening options are to be realized. Thus, in addition to the clinical guidance that already exists, guidance to facilitate effective implementation of cancer screening is required.

To advance quality improvement in the implementation of cancer screening programs, Cancer Care Ontario's (CCOs) Division of Prevention and Screening, in partnership with CCOs Program in Evidence-based Care, established the Cancer Screening Uptake Expert Panel (the Panel) (Additional File 1). Its mandate was to identify and recommend appropriate population-based and provider-based interventions to increase the uptake of screening for breast, cervical, and CRCs. To this end, a systematic review targeting ten interventions was undertaken by the Panel that ultimately served as the evidentiary base underpinning the development of an implementation guideline for this context. The specific guideline question we asked was: What interventions have been shown to increase the uptake of cancer screening by individuals, specifically for breast, cervical, and CRCs? Interventions of interest include:

1. Population-based interventions aimed to increase the demand for cancer screening:

a. client reminders and client incentives

b. mass media and small media

c. group education and one-on-one education

2. Population-based interventions aimed to reduce barriers to obtaining screening: reduction in structural barriers and reduction in out-of-pocket costs

3. Provider-directed interventions targeted at clinicians to implement in the primary care settings: provider assessment and feedback interventions and provider incentives

Our outcome of interest was completed screening rates.

## Methods

### Overview

A multi-step strategy was used to develop the systematic review. A scoping review was undertaken to identify high-quality practice guidelines or systematic reviews for adaptation. The original search yielded a systematic review by Jepson *et al*. [7]; it served as a base upon which a formal systematic review strategy was designed. Our original goal was to extend and update the Jepson review and search for literature published up to July 2008 (date this project was initiated). However, when the formal search strategy was executed, three more current alternative systematic reviews published in a July 2008 special issue of the *American Journal of Preventive Medicine *(AJPM) were identified [8-10]. While other reviews were available, we chose the AJPM bundle based on their direct relevance to the objectives of our project, their currency, and their quality. They served as our taxonomy of interventions and as an evidentiary foundation from which we conducted an update of the literature. This study reports on the update.

### Literature search strategy

An initial literature search update of the AJPM systematic reviews was conducted in the summer of 2008, and a second literature update search was conducted in summer 2010 in response to the quickly developing evidence base. Between the two updates, systematic searches covering 2004 to 2010 were conducted in MEDLINE (2008 July week 4 and 2010 May week 1), EMBASE (2008 week 32 and 2010 week 20), CINAHL (2008 August week 1), and PsycINFO (2008 July week 5 and 2010 May week 1) databases for randomized controlled trials (RCTs), and cluster RCTs assessing the impact of interventions, targeting either the public or healthcare providers, on breast, cervical, and CRC cancer screening rates. Note in our second update, we did not include the CINAHL database because of the poor return of relevant studies found in our first update experience. Reference sections of retrieved review articles were used to obtain additional articles not found by the formal searches, and Panel members were canvassed to determine if there were additional resources and sources of information that ought to be considered. The search strategies used are outlined in Additional File 2.

### Study selection criteria

#### Inclusion criteria

1. Study type/design: RCTs or cluster RCTs.

2. Study intervention: Client reminders, client incentives, mass media, small media, group education, one-on-one education, reducing structural barriers, reducing out-of-pocket costs, provider audit feedback and provider incentives. An operational definition of each intervention is presented in Table 1.

**Table 1:** Definitions of interventions.

Intervention	Systematic review intervention definition
Client Reminders	Printed letter or postcard or telephone communications that were client-tailored or untailored interventions and reminder or recall notifications. Could include one or more of follow-up printed or telephone reminder; additional text or discussion with information about barriers to screening; or appointment scheduling assistance.

Client Incentives	Small, non-coercive rewards (cash or coupons) motivating people to obtain screening for selves or others.

Mass Media	Community or larger-scale intervention campaigns, including television, radio, newspapers, magazines, and billboards. Interventions usually linked to other ongoing interventions.

Small Media	Included videos or tailored or untailored printed materials, such as letters, brochures, pamphlets, flyers, or newsletters distributed by healthcare systems or community groups.

Group Education	Conducted by a variety of healthcare educators through a variety of formats, for a variety of groups, and in a variety of settings.

One-on-One Education	In-person or telephone, tailored or untailored communication delivered by healthcare professionals, lay health advisors, or volunteers in a variety of settings.

Reducing Structural Barriers	Interventions that facilitate removal of non-economic barriers to accessing screening, for example by: reducing time or distance between screening location and target group; modifying hours of service; offering services in alternative settings (mammography vans); and eliminating/simplifying administrative process or other obstacles (*e.g*., scheduling, transportation, translation services). Could be combined with one or more secondary interventions: print/telephone reminders, cancer screening education, screening availability information.

Reducing Out-of-Pocket Costs to Clients	Removal or decreasing of economic barriers restricting access to screening (*e.g*., subsidizing screening through use of vouchers, reducing co-payments or other up-front client-borne expenses, reimbursing clients or clinics after services have been rendered, or adjusting the cost of federal or state insurance coverage. Could be combined with secondary supporting measures: cancer screening education, availability information, structural barrier reduction (*e.g*., assisting with language and cultural barriers; streamlining appointment scheduling).

Provider Assessment and Feedback	Involved evaluation of provider performance in delivering or offering screening to clients (assessment) and presenting providers with information about their performance in providing screening services (feedback). Could involve either group or individual practices, with possible comparison to goal or standard.

Provider Incentives	Direct or indirect rewards (monetary or non-monetary) that motivate providers to perform or make appropriate referral for cancer screening services. Assessment component, with or without feedback, might be included in intervention.

3. Clinical context: Eligible cancer screening modalities included mammogram (breast), Papanicolaou (Pap) test (cervical), and fecal occult blood test (FOBT), flexible sigmoidoscopy (FS), or colonoscopy (colorectal).

4. Study comparisons: One intervention or one combination of interventions versus no intervention; one intervention or one combination of interventions versus an alternative intervention or combination of interventions.

5. Outcome: The primary outcome of interest was the screening rate.

6. Publication type: Full reports.

7. Publication year: Studies published from November 2004 (last search date by the original reviews [8-10]) to May 2010.

#### Exclusion criteria

1. Studies published in languages other than English were excluded because translation services funding was not available.

2. Given that there is varied opinion whether or not there is a role for prostate-specific antigen (PSA) screening for prostate cancer in asymptomatic men at a population-based level, and thus, no agreement whether screening rates should be going up or down, we did not include studies aimed at interventions to increase this screening technique (see http://www.cancercare.on.ca/common/pages/UserFile.aspx?fileId=44610).

There are two important differences in these updated search criteria in contrast to the original systematic reviews. First, to manage scope and size, we restricted our study design criteria to RCTs and cluster RCTs. Second, we did not update the literature on economic efficiency, as was done in the original reviews, due to a lack of confidence about the generalizability and applicability of findings across health system contexts. The reader is directed to the original reviews [8-10] for details on these data.

#### Quality appraisal

The quality appraisals of the original systematic reviews were done using the Assessment of Multiple Systematic Reviews (AMSTAR) tool [11] (Additional File 3). The RCTs and cluster RCTs were evaluated along eight criteria: funding, randomization method, baseline characteristics, blinding, statistical power, achievement of target sample size, follow-up, and intention-to-treat analysis. While several tools and methodologies are available to appraise primary evidence [12], these criteria were chosen as they have been shown to be linked to potential biases in the study designs of interest and are used in the Risk of Bias tool by the Cochrane Collaboration [13].

#### Outcomes and synthesis of data

Overall intervention effectiveness, the primary outcome, was measured by screening completion (self-report or by record reviews). This was calculated as the overall median post-intervention increase (PII) in completed screening tests. This was represented as absolute percentage point (PP) change and either interquartile interval (IQI) when seven or more data points were available or range in all other cases. It is important to note that in the original reviews, different formulae were used to calculate PP change, depending on availability of data and study design [14].

For studies in which there were both baseline and post-test data, the PP was calculated by subtracting the difference between the number of control group individuals screened after and before the intervention time interval from the number of intervention group individuals screened after and before this interval. In contrast, in studies where there were post-test data only, the PP was calculated by subtracting the number of control group individuals screened from the number of intervention group individuals screened after the intervention time interval. In studies where more than one intervention was tested, PPs were calculated for each intervention tested.

Post-intervention results given in included studies as a percentage (relative) change from baseline or as odds ratios (ORs) that could not be converted to PP absolute changes were reported separately. Each included study determined screening completion by either client self-report or record reviews (Additional File 4).

As in the original systematic reviews, given the extreme heterogeneity we found among the eligible studies with respect to execution of interventions and metrics used to calculate screening, overall rates of absolute effectiveness (*i.e*., across studies) were not calculated in this update.

## Results

### Literature search results

#### Original review

As described, three original systematic reviews targeted ten interventions served as the foundation [8-10]. Table 1 provides the operational definition used to categorize the interventions from these reviews -- these definitions were used in the update. Overall quality of the original systematic review was adequate (Additional File 3). The number of eligible studies found per intervention pair in the original reviews ranged between 11 and 42, as described below:

1. client reminders and client incentives: 34 eligible studies

2. mass media and small media: 36 eligible studies

3. group education and one-on-one education: 42 eligible studies

4. reducing structural barriers and out-of-pocket costs for clients: 25 eligible studies

5. provider feedback and provider incentives: 11 eligible studies

The quality of primary studies in the original reviews was generally poor.

#### Update: new trials

**Figure 1:**
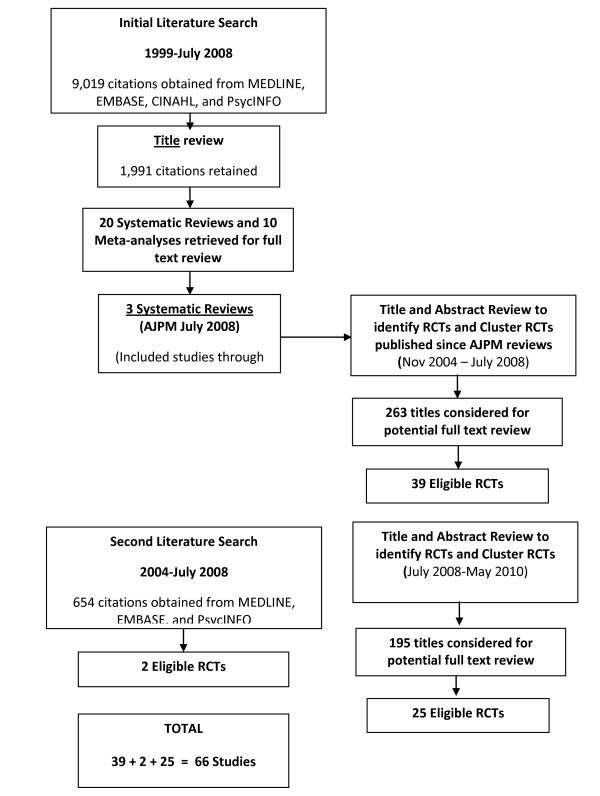
**Literature Search Results**.

Overall, 66 new RCTs and cluster RCTS reflecting 74 comparisons met inclusion criteria [15-80] (see Figure 1). The study quality ranged between poor and excellent. A description of the literature results for each cluster of interventions is described below.

#### Client reminders and client incentives

The literature search yielded 18 new RCTs and clustered RCTs published from November 2004 to May 2010 that met our eligibility criteria [15-32]. All were related to client reminders. A summary of key quality characteristics for the 18 RCTs included and a detailed summary of the outcome results are provided in Additional Files 5 and 6. Overall, the body of evidence is of weak to moderate quality.

#### Mass media and small media

The literature search yielded 23 new RCTs and cluster RCTs published from November 2004 to May 2010 that met our eligibility criteria [20,29,32-52]. All were related to small media interventions. A summary of key quality characteristics for the 23 included RCTs and a detailed summary of the outcome results can be found in Additional Files 7 and 8, respectively. The body of evidence ranges from weak to excellent quality.

#### Group education and one-on-one education

The literature search yielded 18 new RCTs and clustered RCTs published from November 2004 to May 2010 that met our eligibility criteria [53-70]: five targeting group education, 12 targeting one-on-one education, and one targeting both interventions. Data summaries of key quality characteristics and outcome results for the included RCTs can be found in Additional Files 9 and 10. Overall, the body of evidence is of moderate quality.

#### Reducing structural barriers and out of pocket costs

The literature search yielded six new RCTs published from November 2004 to May 2010 that met our eligibility criteria [36,54,58,71-73]. A summary of key quality characteristics for the six included RCTs and a detailed summary of the outcome results can be found in Additional Files 11 and 12, respectively. Overall, the body of evidence is of moderate quality.

#### Provider feedback and provider incentives

The literature search yielded nine new RCTs and cluster RCTs published from September 2004 to May 2010 that met our eligibility criteria [23,35,74-80]. Data summaries of key quality characteristics and outcome results for the nine included RCTs are provided in Additional Files 13 and 14. Overall, the body of new evidence is of weak to moderate quality.

### Outcomes

#### Client reminders and client incentives

##### Breast cancer/client reminders

Seven studies reported on eleven intervention arms fitting the definition of client reminders [15-21]. One study reported a significant increase in breast cancer screening for the tailored telephone plus print client reminder intervention over the usual care control group: 12.0 PP increase; OR = 1.9; p = 0.001 [15]. Three studies reported that tailored telephone reminders also resulted in significantly increased screening in comparison to the control group: 6.0 to 12.0 PP increase; OR = 1.6, p = 0.02 [15]; OR = 1.59_adj_; 95% CI, 1.27, 2.00; p ≤ 0.001 [16]; and p < 0.001 [17]. One of those studies and a fourth had significant results for tailored print client reminder interventions versus control: 9.0 PP increase each; OR = 1.7, p = 0.006 [15] and 64.3% versus 55.3%, respectively, p < 0.001 [18]. One study found that a tailored telephone intervention increased mammography, although non-significantly, compared to a no-intervention control: 7.8 PP increase [19]. Two targeted studies reporting on five client reminder interventions found significant and more robust effects in favour of manual or automated telephone reminders compared to usual care print interventions: 8.0 PP increase; p = 0.004; and 4.5 PP increase; AOR = 1.32; p = 0.014 [20,21]. In the previously mentioned study [21], an enhanced letter reminder only yielded a 2.7 PP increase in comparison to the usual care print reminder.

##### Cervical cancer/client reminders

Four studies reported on four intervention arms fitting the definition of client reminders [16,17,22,23]. Two studies reported that tailored telephoned client reminders resulted in higher cervical cancer screening in comparison to those of the usual care control groups: 13.0 PP increase; OR_adj _= 1.73; 95% CI, 1.31, 2.27; p ≤ 0.001 [16] and 7.0 PP increase; p < 0.001 [17]. A third study dealt with a population-wide reminder letter mail-out intervention compared to a no-letter control group and reported significantly higher Pap test screening overall (p < 0.05) for the intervention group versus the control at the 90-day follow-up: 1.54 PP increase; p < 0.05 [22]. The fourth study had modest results favouring an intervention strategy employing the delivery of a targeted letter signed by the patient's physician in combination with a facilitator visit to evaluate provider screening practices: 1.97 PP increase; OR = 1.17; p < 0.036 [23].

##### Colorectal cancer/client reminders

Eleven studies involving sixteen intervention arms dealt with colorectal screening interventions based on client reminders [16,17,24-32]. Six studies [16,17,24,25,28,29] looked at uptake results for all three colorectal screening tests combined. Two found that personalized telephone reminder interventions, with mailed educational print material, resulted in higher colorectal screening adherence in the intervention group versus control group: 15.0 PP increase; OR_adj _= 1.92; 95% CI 1.49, 2.47; p ≤ 0.001 [16] and 13.0 PP increase; p < 0.001 [17]. The third study, which used the Insure^® ^Fecal Immunochemical Test [FIT] rather than the gFOBT, reported significantly higher overall CRC screening test uptake for all three intervention arms in comparison to the control group for both print and print plus telephone reminders [24]. Differences were more robust for participants who actually received the intervention in comparison to the intention to treat analysis [24].

Another study, a cluster trial that looked at uptake for the three CRC screening tests, used a physician-signed personalized reminder letter with educational material and an FOBT kit as an intervention [25]. The study found no difference in screening uptake for any screening test at two years: 0.02 PP increase; p = 0.51 but did find a significant increase for FS testing in the intervention arm at five years: 3.0 PP increase; p < 0.01 [25]. However, it is unclear whether this trial made adjustments for the design effect associated with cluster randomization. Of the two remaining studies considering all forms of CRC testing, one used a computerized system to deliver reminder forms to three intervention arms (clinicians only, patients only, and both) and found significant overall improvement in screening rates across all arms in comparison to baseline: average 9 PP increase; p = 0.002 [28]. It is important to note that results for each intervention arm were not given. The final study reported a modest increase of CRC screening uptake in the multilingual clinic posters plus reminder call intervention in comparison to the poster only and usual care arms: 0.5 PP increase and 1.5 PP increase, respectively [29]. The additional phone reminder was most successful in the subset of patients overdue for CRC testing compared to usual care results: OR 1.49; p = 0.001. This cluster trial did not adjust for design effects, thus a unit of analysis error has possibly skewed significance test results [29].

Two studies directed interventions at colonoscopy screening uptake, using personal navigators to provide telephone reminders and motivational support [26] as well as print reminders and educational material [27]. Both studies reported higher test completion for the intervention group than for the control group: 40.8 PP increase; p = 0.058 [22] and 11.7 PP increase; p = 0.001 [27]. Another three studies focused on FOBT uptake by providing patients with reminders, an FOBT kit, and educational materials [30-32]. The print and telephone reminder intervention studies had substantially higher odds of FOBT card return: 16.2 PP increase; AOR 2.02; 95% CI 1.48, 2.74; p < 0.001 [30]; and 25.4 PP increase; OR 11.3; 95% CI 5.8, 22.0 [31]. The third study found mixed results of an email versus mail reminder system in the private and public access groups. The intervention was successful in the former: 3.0 PP increase; but the control outperformed the intervention in the latter: -33.0 PP decrease [32]. The researchers of the pilot study attributed the poor results to problems addressing system and access barriers faced by participants.

##### Client incentives

No studies were found that looked at client incentives alone as an intervention to increase breast, cervical, or CRC screening uptake.

##### Client reminders and client incentives - summary and interpretation

Fifty-two studies comprise the complete evidentiary base: 34 from the original review [see [8]] and 18 from the update [15-32]. All evidence focused on the client reminders. No studies were found that met inclusion criteria in either the original review or the update regarding client incentives.

In the original review, Baron *et al*. [8] concluded that there was strong evidence such interventions increased both breast and cervical screening, especially with the addition of other messages or forms of intervention. However, the evidence did not exist to demonstrate a similar impact of those 'enhancements' on never-screened or hard-to-reach women. Sufficient evidence existed to show that client reminders increased guaiac-based FOBT (gFOBT) screening. Across the cancer screening sites, the percentage point increase (PPIs) ranged from 10.2 to 14.0.

Eighteen new RCTs were found [See Additional Files 5 and 6]. PPIs ranged from 2.7 to 12.0 for breast cancer; 1.54 to 13.0 for cervical cancer, and -33.0 to 40.8 for CRC. It is important to note, however, that the quality of the RCTs is questionable; the reporting of key quality domains (method of randomization, blinding, *et al*.) was universally incomplete. Thus, despite the high level of evidence we considered, the execution of these studies may be such that bias has been introduced.

For those studies targeting breast and cervical screening, eight of eleven showed statistically significant differences in screening uptake favouring the intervention groups, further supporting the Baron *et al*. [8] findings. The effective interventions profiled in these studies were tailored reminders, both telephone and print, and in addition, a large-scale reminder letter mail-out for cervical screening. For the effect of client reminder interventions on colorectal screening, five studies reported significant increases for the three CRC screening tests overall (although one study used immunochemical rather than gFOBT), one study reported significantly higher uptake for FS testing for colonoscopy, and two other studies reported increased FOBT screening. The study results add support to the Baron *et al*. [8] positive findings for the impact of client reminders on FOBT screening and demonstrate that they could improve FS and colonoscopy rates. Effective interventions included tailored telephone reminders enhanced with educational materials and/or personal navigators.

#### Mass media and small media

##### Mass media

No studies were found that looked at mass media alone as an intervention to increase breast, cervical, or CRC screening uptake.

##### Breast cancer/small media

Seven studies [20,33-38] involving eleven intervention arms looked at the impact of small media interventions on breast cancer screening uptake, in comparison to control groups. One study reported increased screening for three intervention groups consisting of personalized invitation letters with or without reminder letters or telephone calls versus the comparison group: one letter, 4.1 PP increase; two letters, 7.1 PP increase; p = 0.05; one letter plus telephone call (available telephone number) 11.9 PP increase; p = 0.001 [33]. Another study implementing three intervention strategies found automated telephone reminders more successful than the usual care print equivalent: 4.5 PP increase; OR 1.32; 95% CI, 1.06, 1.64; p = 0.014; whereas an enhanced letter reminder containing a breast cancer booklet placed second but with a non-significant increase in screening: 2.7 PP increase; OR 1.19; 95% CI 0.96, 1.48; p = 0.117 [20]. A third study, a cluster trial using trained staff to deliver short scripted loss-framed messages by telephone plus appointment scheduling assistance, reported significantly higher odds of mammograms in the intervention arm versus the control: 11.9 PP increase; OR_adj _= 1.914; χ^2 ^= 7.48; p = 0.0063; 95% CI, 1.20, 3.05 [34]; however, it is unclear whether this study made adjustments for the design effect associated with cluster randomization. A fourth study showed only a small significant increase in the intervention group screening for mailed educational materials plus telephone counselling: 4.2 PP increase; p = 0.02 [35]. The remaining three studies were not as promising [36-38]. One study reported a cultural tailored pamphlet plus recommendations faired poorly against monthly health advisor sessions plus access enhancing services: -32.8 PP decrease; OR = 0.21; p < 0.0001 [36]. The last two studies [37,38] concluded there was limited evidence for either intervention group being more effective than the control group when using tailored and targeted educational materials versus targeted materials only.

##### Cervical cancer/small media

Three cervical screening studies that involved five small media intervention arms [39-41] looked at the impact of small media interventions on cervical screening uptake. In one study, brief automated interactive voice response educational telephone calls resulted in only a slight overall increase in uptake at three months for the intervention group (0.43%), compared to the control group, that then decreased over time. However, subgroup analysis found a higher increase for the more at-risk intervention age 50 to 69 group at six months (1.35% increase; 95% CI, 1.28, 1.42), and the intervention was described as a 'feasible' option [39]. Personalized letters, educational material, and telephone follow-up resulted in significantly higher cervical screening for one study intervention group: OR = 2.29; p = 0.002 [40], while in another study only a letter signed by the public health doctor resulted in a small but non-significant increase in screening at three-month follow-up compared to the control group: 2.8 PP increase [41].

##### Colorectal cancer/small media

Thirteen studies compared colorectal screening uptake in 20 intervention arms to that in control groups. Eight of the studies involved all three colorectal screening tests (FOBT, FS, and colonoscopy) [29,42-48]. Four studies used FOBT [32,51,52], and one study used colonoscopy [49].

Two studies had intervention participants individually view an educational video, either in clinic [43] or mailed to home [42]. One study reported a non-significant difference (p = 0.61) in screening, favouring the control group [43], but the second reported a significant increase in screening uptake for the intervention group for those participants who actually watched the video: 17.6 PP increase; OR = 2.81, 95% CI 1.85, 4.26 [42]. A third study, which had intervention participants individually use an interactive educational CRC website, reported the intervention group was significantly more likely at 24 weeks follow-up to be screened for any test than the control group that viewed a standard non-interactive site: 26.0 PP increase; p = 0.035 [44].

One study that used customized mailed print booklets reported a non-significant difference in adherence between the tailored intervention and not tailored comparison group, favouring the comparison group, for the uptake of any screening test at three-month follow-up: 7.0 PP increase; p = 0.30 [45]. A separate mailed educational intervention study conducted on first degree relatives of CRC patients found a non-significant increase of screening activity in support of standard care: -2.0 PP increase; p = 0.91 [46]. In a study comparing untailored mailed print material to tailored and re-tailored material, follow-up at 14 months showed that only multiple tailored print mail-outs had significantly better results compared to the control group: 9.0 PP increase; p = 0.03 [47]. Personalized letters, educational material, a FOBT kit and contact information to schedule a colonoscopy/FS as an alternative were mailed out to intervention patients resulting in significantly higher screening rates: 5.8 PP increase; p < 0.001. The mailings primarily increased the return of FOBT cards and the intervention effect increased with age: 50 to 59 y, 3.7 PP increase; 60 to 69 y, 7.3 PP increase and 70 to 80 y, 10.1 PP increase [48].

Another two studies utilized comparable intervention methods and found similar results [49,29]; however, one study only considered colonoscopies. Compared to the usual care arms, both studies reported that all four intervention arms show a moderate statistically significant increase in up-to-date CRC screening. However, in both cases, small media alone in the form of a culturally tailored booklet or clinic poster faired only slightly lower than a combined intervention strategy of small media plus telephone discussion (11.2 versus 12.2 PP increase and 3.5 versus 4.0 PP increase, respectively [49,29]). The additional time and expenses of a single telephone session were deemed inefficient, because it did not add significantly to treatment effects. It is important to note that one cluster trial [29] did not adjust for cluster effects leading to potentially skewed result.

The four remaining studies involved only FOBT, either guaiac-based or immunochemical (FIT) [32,50-52]. The study using FIT compared three interventions to a control standard invitation letter, and found a significantly increased screening uptake for the intervention group receiving advance notice of the invitation letter compared to the control group at 12 weeks: 8.8 PP increase; RR = 1.23; 95% CI, 1.06, 1.43 [51]. One study using gFOBT found no significant difference in completion between the usual care (education by nurse) and intervention group (educational computer program): 1.0 PP difference favouring the usual care nurse education over the intervention; p = 0.89 [50], but suggested the similar results meant that the computer program could be a resource-saving choice. The final two studies reported a substantial increase in FOBT card returns by using an educational video intervention or educational sheets plus reminder calls: 15.2 PP increase; OR = 2.0; p = 0.044; and 25.4 PP increase; OR = 11.3; p < 0.001 [52,32].

##### Mass media and small media: summary and interpretation

The systematic review yielded very different results for the effectiveness of mass media alone and small media alone. In all, 57 studies met inclusion criteria: 34 in the original review [see [8]] and 23 in the update [20,29,32-52].

With respect to mass media alone, the original systematic review failed to yield studies that met eligibility criteria. So too did the update. However, it should be noted that studies examining the effectiveness of mass media may more typically use study designs other than those considered in the update. For example, time series or before-after designs may be the more appropriate strategy to evaluate the role of mass media, given the inherent challenges of managing potentially confounding exposure between the control and intervention groups. Thus, while there is insufficient evidence to support or refute the role of this intervention to facilitate the uptake of screening given the criteria we used, studies using other designs may have yielded different conclusions.

In contrast to the lack of evidence for mass media, there is an abundance of evidence to recommend the use of small media to increase rates of breast, cervical, and CRC screening in the general population. Baron *et al*. [8] concluded that strong evidence existed to show that small media interventions increased breast and cervical screening, as well as colorectal screening for gFOBT, across a range of populations and settings, with the percentage point increases (PPIs) ranging from 4.5 to 12.7.

Twenty-three new RCTs were found examining the role of small media to increase the uptake of cancer screening. While the reporting of study quality was generally incomplete, where it existed, the quality of the studies appeared adequate: methods of randomization and blinding strategies aligned with current methodological norms, baseline characteristics were generally balanced, and statistical methods appropriate. PPIs ranged from -2.1 to 11.9 (outlier: -32.8), 1.35 to 2.8, and 1.0 to 26.0, for breast, cervical, and CRC screening, respectively.

Three of seven and two of five studies targeting breast and cervical screening respectively, found a significant increase in screening favouring small media. Brief telephone messages, including an interactive voice response system or personalized invitation letters enhanced by telephone follow-up were profiled in these studies. These results further support those reported by Baron *et al*. [8] for small media interventions. In contrast, however, three of the four remaining breast cancer studies incorporated small media print materials reported the intervention did not increase overall mammography rates creating doubt in the value of print-alone small media strategies.

In contrast to Baron *et al*. [8], some evidence in favour of small media was found for a range of screening CRC screening modalities (gFOBT, FS, or colonoscopy). Here, small media involving a specific interactive website intervention (any test), advance notification of an invitation letter (FIT), an educational video (FS), and educational booklet plus newsletter mail/phone call indicate possible interventions that could be pursued. Nine of thirteen studies reported a significant increase in CRC screening for the intervention arms. The most successful studies implemented educational videos, websites, or information sheets. Mailed education materials with or without telephone communication were also successful, however the added telephone intervention was found to be resource inefficient when compared to mailed intervention alone.

#### Group education and one-on-one education

##### Breast cancer/group education

One study [53] looked at the impact of group education on breast cancer screening uptake and reported no significant difference for the intervention group compared to the control group overall: 8.0 PP increase; OR = 1.26; 95% CI 0.74, 2.14, p = 0.39. However, there was a significant increase for the intervention arm in a subgroup of women who knew about mammograms but had never been screened: 16.0 PP increase; OR = 1.99; 95% CI, 1.03, 3.85, p = 0.04. A second study found that combined media and lay health worker educational outreach intervention to have a significantly larger effect size than the comparison group of media education alone for Vietnamese women [54]: 14.2 PP increase; OR = 3.21; 95% CI, 1.92, 5.36. The final study found no significant differences between the control group and the social network support/education group for either age strata considered (40 to 51 y and ≥ 52 y) [55].

##### Cervical cancer/group education

A single study was found that looked at group education alone as an intervention to increase cervical screening among Samoan women. Culturally tailored interactive group discussion sessions supplemented by educational booklets significantly increased Pap smear use, favouring the intervention group: 23.4 PP increase; OR = 2.0; 95% CI, 1.3, 3.2; p < 0.01 [56]. However, it is important to mention that the clustering of groups were not factored into the analysis.

##### Colorectal cancer/group education

Two studies found in the update reported on group education interventions for CRC. The first study compared two types of culturally relevant group education presentations for Native Hawaiians about FOBT [57], using a slide presentation by a non-Hawaiian nurse as the control group and a more complex culturally targeted presentation by a Native Hawaiian doctor and presenters as the intervention group. However, after randomization, 64% of participants were found to be already up-to-date with CRC screening. For the unscreened, the control presentation proved to be very slightly more effective than the intervention group at motivating adherence. The second study targeted towards increasing CRC screening among African Americans compared group education, one-on-one education, or financial support to usual care [58]. The group education cohort was the most successful intervention, nearly doubling the rate at which participants were screened in comparison to the usual care group: 9.7 PP increase. Statistical significance was reached when the subset of contactable patients was considered in the analysis, but not when using an intention to treat analysis for all enrolled participants. While one-on-one education and financial support also showed promise, neither reached statistical significance. It is unclear whether the analyses adjusted for group allocation.

##### Breast cancer/one-on-one education

Four studies involving four intervention arms utilized one-on-one education [59-62]. One study [59] found no difference between the intervention, consisting of educational and actively supportive telephone calls plus print educational material, and the comparison group: 2.0 PP increase; OR_adj _= 1.16; 95% CI, 0.86, 1.57, p = 0.33. The second study, a cluster trial that provided one-on-one culturally sensitive and tailored education through a lay health advisor as an intervention, reported statistically significant increases in breast screening in the intervention group, compared to the control group [60]. The increase was not only significant overall within 12 months of the intervention: 15.2 PP increase; RR = 1.56; 95% CI 1.29, 1.87, p < 0.001 [60], but also within racial groups: African Americans, RR = 1.54; 95% CI, 1.11, 2.14, p = 0.008; Native Americans, RR = 1.58; 95% CI, 1.18, 2.13, p = 0.002; and whites, RR = 1.54; 95% CI, 1.05, 2.25, p = 0.024. However, it is unclear whether this trial made adjustments for the design effect associated with cluster randomization. The third study reported a significant increase in mammography for an educational telephone counselling intervention compared to a mailed information intervention within one year of the first intervention contact: 12.6 PP increase; p = 0.04, although the difference became non-significant (p = 0.29) after the second contact a year later [61]. The final study used lay health workers to set up one-on-one discussion sessions culturally tailored towards low literacy Hispanic farm women [62]. Mammography screening was higher among women in the intervention group for those who completed the follow-up: 10.9 PP increase. The intention to treat analysis, however, failed to demonstrate a significant increase: 5.0 PP increase, p > 0.05.

##### Cervical cancer/one-on-one education

One study identified for this category found no difference between the intervention, consisting of educational and actively supportive telephone calls plus print educational material, and the control group: 1.0 PP increase; OR_adj _= 1.18 (0.82, 1.70), p = 0.38 [63]. A second study also found no significant differences using lay health workers to promote Pap smear use in low literacy Hispanic farm women: 5.3 PP increase; p > 0.05 [62]. However, a separate analysis among those women who responded for follow-up reported a significant intervention effect for cervical screening completion in the intervention arm: 15.9 PP increase; p < 0.05.

##### Colorectal cancer/one-on-one education

Ten studies involving 14 intervention arms dealt with the effect of one-on-one education on colorectal screening uptake, including tailored and/or scripted telephone counselling plus other educational interventions [59,63-67] and in-person education sessions with culturally equivalent nurses or clinic nurses [68,69]. Six studies looked at all three colorectal tests (FOBT, FS, and colonoscopy) [58,59,63-65,70].

For all three CRC tests, one study found that an intervention consisting of educational and actively supportive telephone calls plus print educational material resulted in higher CRC screening adherence in the intervention group compared to the comparison group: 7.0 PP increase; OR_adj _= 1.69; 95% CI, 1.03, 2.77, p = 0.04 [59]. Another study reported significant uptake of all tests at six months follow-up by the tailored telephone intervention group, an uptake 4.4 times higher than for the control group: 20.9 PP increase; RR = 4.4; 95% CI, 2.6, 7.7 [63]. A third study reported that, overall, the intervention did not increase CRC screening when compared to the control group [64]. However, when the analysis looked at the telephone counselling intervention subgroup actually reached by telephone, in comparison to the 'no call' and control groups, there was a highly significant difference in favour of the intervention subgroup: 7.0 PP increase; p < 0.0001 [64]. The fourth study involving all three screening tests reported no significant differences in screening uptake between tailored and untailored interventions groups [65] in promoting or maintaining screening. The final two studies failed to find a significant difference in favour of an automated telephone outreach or health education session [58,70].

The one study that looked at FOBT and FS uptake results reported non-significant increases for the intervention group compared to the control group at three months follow-up (FOBT, p = 0.086; FS, p = 0.115), but a significant increase at six months for FS: 18.7 PP increase; p < 0.019 [66]. A study involving colonoscopy uptake in poor attendees at screening found a significant difference in favour of the one-on-one education group over the brochure group: OR_adj _= 2.14; 95% CI, 0.99, 4.63, p = 0.05 [67].

The two studies using FOBT found significantly higher screening completion for the educator intervention groups versus control: 41.9 PP increase; OR_adj _= 6.38; 95% CI, 3.44, 11.85 [68] and 14.6 PP increase; p < 0.001 [69].

##### Group education and one-on-one education: summary and interpretation

A total of 60 studies met inclusion criteria in this systematic review: 42 from the original review [8] and 18 found with the update [53-70]. The evidence regarding the role of group education interventions for the general population is incomplete and inconsistent with respect to direction of findings and magnitude of effects. The most promising evidence regarding the effectiveness of group education was found in studies with interventions aimed at specific communities. Thus, this intervention may be appropriate for special populations (*e.g*., populations for whom access is challenging), but more study in this area is warranted.

In contrast, the evidence regarding one-on-one education appears more compelling. In the original review, Baron *et al*. [8] determined there was strong evidence for an increase in breast and cervical cancer screening with one-on-one education, for both tailored and untailored interventions. However, insufficient evidence existed to determine the effectiveness for that type of intervention in increasing CRC screening.

Significant increases in breast screening rates for one-on-one education for both face-to-face and telephone interventions were found in this update (two of four studies), supporting the original review. However, no significant difference between groups for cervical screening was found in the two studies when an ITT analysis was used. This contrary evidence, however, did not provide a compelling argument to sway interpretation of the totality of evidence to a different conclusion from that of Baron *et al*. [8].

For CRC screening, the new studies found significant differences for CRC screening test uptake, in favour of the one-on-one education interventions, for CRC screening overall (two studies), colonoscopy uptake (one study), FOBT uptake (two studies), and FS (one study). Three of the remaining four studies also reported increases in the intervention arm, but not to a significant effect. There are challenges with these studies, including significant differences emerging in subgroup analyses only, variability between groups at baseline, variability in the magnitude of effect, and overall quality concerns with the studies. Thus, although there was general consistency in the results, the limitations of the new evidence preclude us recommending this suite of interventions at this time. Rather, we believe the new studies provide emerging evidence regarding the potential use of one-on-one education as a strategy to facilitate CRC screening.

#### Reducing structural barriers and out-of-pocket expenses

##### Breast cancer/reducing structural barriers

Two studies were found to reduce structural barriers in breast cancer, specifically minority groups of Vietnamese and African American women, respectively. The first study tested a combined intervention strategy of media education plus lay health worker outreach, and reported a significant intervention effect size for mammography uptake versus the media education alone comparison group: 14.2 PP increase, OR = 3.21; 95% CI, 1.92, 5.36. The additional lay health worker outreach was able to increase screening by providing participants with access enhancing services [54]. The second study compared low-dose intervention of a culturally tailored pamphlet with screening recommendations versus a high-dose intervention of health advisor sessions, low-cost referrals, scheduling assistance, and transport services [36]. The high-dose intervention improved mammography screening rates in low income African-American women: 32.8 PP increase; OR = 4.7; 95% CI, 2.4, 9.4; p < 0.0001.

##### Cervical cancer/reducing structural barriers

One study was found that looked at the impact on cervical screening uptake of interventions to reduce structural barriers through promotive efforts [71]. The intervention was aimed at meeting participants' stated requirements of friendly treatment and/or of suitable appointment times in order to provide a cervical smear. A significant increase (p < 0.0001) in screening uptake was seen in the intervention group versus the control group: 11.0 PP increase, with the implementation of changes such as alternative clinic sites, after-hour appointments, offering transport, and utilizing specially chosen examiners.

##### Colorectal cancer/reducing structural barriers

One study utilized culturally tailored materials delivered by a health navigator, who also reviewed available methods, schedules appointments, translated materials, and organized transportation. This intervention was found to be successful as colorectal screening rates were significantly higher that of the control group: 15.6 PP increase; p < 0.001 [72].

##### Breast cancer/reducing out-of-pocket costs

One study comparing two interventions to a control reported on the effect of a monetary incentive on mammography uptake [73]. The two personally addressed mailer interventions significantly increased mammogram uptake in comparison to a no-intervention control by 0.23% and 0.75%, respectively. One intervention combined the mailers with a monetary incentive provided post-mammogram, a strategy that significantly increased that intervention's effectiveness by 0.52% in comparison to the mailer-only intervention.

##### Cervical cancer/reducing out-of-pocket costs

No studies were found that looked at the impact on cervical screening uptake of interventions to reduce out-of-pocket costs.

##### Colorectal cancer/reducing out-of-pocket costs

One study tested a financial support strategy among African-Americans offering reimbursements for up to US$500 for out-of-pocket costs incurred for CRC screening [58]. The study also looked at one-on-one education and group education interventions compared to usual care. The financial support intervention placed third of those studies reporting moderate screening increases: 4.2 PP increase; p = not significant. However it is unclear if this trial appropriately adjusted for the effect of clustering.

##### Reducing structural barriers and out-of-pocket expenses: summary and interpretation

Our review yielded mixed results. A total of 31 studies met inclusion criteria: 25 from the original review [see [9]] and six found with our update [36,54,58,71-73]. With respect to reducing structural barriers, the original review by Baron *et al*. [9] determined strong evidence for the effectiveness in increasing breast cancer screening (using mobile vans or providing free transportation) and CRC screening specifically utilizing gFOBT (particularly through mailing a kit with return postage) but insufficient evidence to support this type of intervention to improve cervical cancer screening rates. The three new studies align with these findings: significant increases in breast and CRC screening rates were found for lay health workers providing access enhancing services in combination with secondary interventions. New RCT evidence was found for cervical cancer screening favouring the use of barrier reduction interventions (PPI = 11.0). However, in contrast to breast and CRC screening, where we believe there is sufficient evidence in the original and newer studies to recommend interventions aimed to reduce structural barriers, the Panel does not believe there is sufficient evidence to support or refute the use of these strategies for cervical screening.

A challenge with this collection of studies is that the interventions to mitigate structural barriers varied considerably in the types of specific strategies employed. Baron *et al*. [9] did not conduct subgroup analyses to explore the relative magnitude of effect of one strategy (*e.g*., mobile units) versus an alternative strategy (*e.g*., free transportation).

With respect to reducing out-of-pocket expenses, Baron *et al*. [9] concluded that sufficient evidence existed to state that reducing out-of-pocket costs through ensuring screening costs were covered increased breast cancer screening by mammography. The breast cancer study found in the update, while positive, showed small absolute benefits. Evidence for this intervention for cervical or CRC study was incomplete in the original review, and the remaining studies identified in the update for CRC reported no significant differences between the financial support and control groups. No relevant studies were found in the update for cervical cancer. An important consideration for this evidentiary base, as it relates to the Canadian context and similar healthcare systems, is the applicability of the types out-of-cost expenses considered. Specifically, the out-of-pocket expenses considered (*i.e*., free client vouchers and government benefits to offset costs of screening tests) are not relevant to the Canadian context where screening tests for breast cancer, cervical cancer, and CRC (FOBT, FS, and colonoscopy) are paid for in a publicly funded healthcare system. More appropriate interventions for our context might include resources to offset travel costs to the screening centre, to pay for child care, or to offset lost wages. Thus, as it applies to Canada and similar systems, there is insufficient evidence to support or refute the role of reducing out-of-pocket expenses as a mechanism to improve uptake of cancer screening.

#### Provider assessment feedback and provider incentives

##### Breast, cervical, and colorectal cancer/provider assessment and feedback

Eight additional studies published since 2004 were found that met inclusion criteria. One study [74] with one intervention arm looked at the impact of a provider-directed assessment and feedback intervention on cancer screening uptake, specifically for CRC. The study reported a statistically significant increase in CRC screening for the intervention group compared to the control group for completion of FOBT, FS, or colonoscopy: 8.9 PP increase; p = 0.003 [74]. A second study using a practice audit with academic detailing and facilitator feedback was also found to significantly increase mammography rates: 17.0 PP increase, p = 0.015 [75]. A third study implementing a provider assessment and feedback intervention reported significant increases in screening rates for breast cancer: 20.0 PP increase; p = 0.04, but not for CRC: 0.0 PP increase [76].

Five other studies looked only at the assessment of the service delivery component of provider-directed interventions and reported results in terms of the intervention impact on screening [23,35,77-79]. One study, which looked at the effect of an intervention on the delivery of 13 preventive health manoeuvres, found differences in favour of the cancer screening intervention: mammography, 37.3 PP increase; Pap smear, 9.0 PP increase; and FOBT 33.3 PP increase, with an adjustment for confounders resulting in a statistically significant increase in favour of the intervention for FOBT: RR_adj _= 6.69; 95% CI 1.85, 24.17, p ≤ 0.05, and a slight increase for mammography: RR_adj _= 1.41; 95% CI, 0.76, 2.61 [77].

Another study intervention provided quality enhancements for cervical cancer screening procedures combined with patient reminders reported a consistent increase in the proportion of women obtaining Pap smears: 3 mos 0.70 PP increase; 6 mos 0.94 PP increase; 9 mos 1.97 PP increase; OR = 1.17; trend test p < 0.036 [23]. A third study found provider assessment and education significantly increased colorectal screening in the intervention group compared to the control: 12.0 PP increase; OR = 2.25; 95% CI, 1.67, 3.04; p < 0.001 [78]. It is important to note that physicians received Strengths, Weaknesses, Opportunities, and Threats (SWOT) analysis to increase practice efficiency as part of the intervention and that CRC screening rates included referrals and completion. The other two studies, both measuring the impact of a practitioner education program on delivery of cancer screening, reported no significant differences between the intervention and control groups for any CRC test completion [79] or for mammography completion [35], with results favouring the control group.

##### Breast, cervical, and colorectal cancer/provider incentives

One Italian study [80] used a provider incentive intervention to compare patient screening compliance for FOBT (guaiac and immunochemical) between hospitals (gastroenterology units; no incentive) and general practitioners (GPs; financial incentive). They reported a significantly higher screening uptake response for GPs (intervention) over hospitals (control): 34.1 PP increase; RR = 3.4; 95% CI, 3.13-3.70.

##### Provider assessment and feedback and provider incentives: summary and interpretation

The total evidentiary base is comprised of 16 studies: 11 included in the original systematic review [see [10]] and nine that met inclusion criteria in the update [23,35,74-80]. Sabatino *et al*. [10] determined that sufficient evidence existed to state that provider assessment and feedback interventions were an effective means of increasing breast, cervical, and colorectal FOBT screening (median PPI 13.0), although the intervention was more effective for trainees than for established practitioners. There was insufficient evidence, however, to determine intervention effectiveness in increasing FS or colonoscopy screening.

The RCT update provided additional evidence to support the Sabatino *et al*. [10] positive finding for the effectiveness of provider assessment and feedback interventions in increasing breast cancer screening and moderately for colorectal FOBT screening uptake. Of the studies that utilized only the provider assessment component and not provider feedback, supporting evidence of effectiveness was found for FOBT, but only marginally for breast and cervical screening.

Together these findings indicate that provider assessment and feedback strategies may be effective in increasing breast, cervical, and colorectal FOBT screening uptake. In contrast to Sabatino *et al*. [10], the new evidence found was strongest for colorectal FOBT screening. Little new work has been done examining this strategy for cervical screening, and this would be an area for additional research to better understand its role.

With respect to provider incentives, while Sabatino *et al*. [10] concluded there was insufficient evidence to support the use provider incentives to increase breast, cervical, or CRC screening, the addition of the new evidence found in the uptake makes this intervention more promising. Although the interventions studied here are not directly relevant to all heath care contexts, equivalent scenarios exist. For example, in Canadian provinces, changes to the provincial fee schedule through the implementation of new items such as preventive care bonuses may yield favourable results in patient care. However, the quality and quantity of the studies in this area make firm conclusions regarding its role difficult.

##### Final conclusions

Sixty-six RCTs and cluster RCTs reflecting 74 comparisons were identified in the update. In summary, and with considered judgement and integration with the data from the original systematic reviews [8-10], the Panel concludes that client reminders, small media, and provider audit and feedback appear to be reasonable strategies to increase the uptake of screening for breast, cervical, and CRCs. In contrast, one-on-one education appears to be an effective intervention to increase the uptake of breast and cervical cancer screening at a population level and a potential intervention to increase the uptake of CRC screening. Similarly, while reducing structural barriers appears to be an effective strategy to increase the uptake of breast and CRC screening, their role in cervical screening is not known. At this stage, more study is required to assess client incentives, mass media, group education, reduction-of-out of pocket cost and provider incentive interventions. Of particular note, context relevant studies are required to better evaluate those interventions dealing with compensation and sources (*e.g*., client incentives, reduction of out-of-pocket costs, and provider incentives) as anticipate that differences between public, private, and mixed healthcare systems may have a significant impact on how these interventions can be designed and executed.

There are clear strengths to the approach we took. This includes an explicit and transparent methodology, high quality critical appraisal, and clear considered judgement regarding the interpretation of the data. In addition, we used three high-quality systematic reviews as our evidentiary foundation [8-10], reducing duplication in effort, which is an important consideration in methods related to systematic review and guideline development. However, there are also some limitations.

While there is a benefit of relying and building upon existing high-quality reviews, one can become somewhat bound by the approach, taxonomy, and organizational framework of the foundational reports. For example, our review did not include all possible interventions that might be relevant to the goal of increasing screening rates (*e.g*., educational interventions for professionals). It was beyond the scope of this review, given the resources for the project, to fill in these gaps. This is an area for future study.

Similarly, following the style of the foundational reports, our review was organized as of function of implementation strategy. An alternative approach would have been to organize according to screening site (breast, cervical, CRC). As described above, this review serves as the evidentiary source for an implementation guideline for the Ontario, Canada cancer system. Given the increased focus on integrated screening, we believe our organizational approach to the evidence base better serves this agenda. However, for other contexts, a different organizational structure may better serve the needs of the stakeholders and knowledge users.

Another limitation is in regard to the strategy used to measure change in screening rates and how one interprets these data. The original systematic reviews calculated individual absolute PPs from each study, using one of three methods, depending on availability of the data, and then combined PPs across studies (Additional File 4). The absolute PPs for the new studies emerging in the update were calculated using these methods. While we know larger PP values are more desirable, because they come from a range of different data elements, we cannot conclusively provide accurate estimates on the absolute impact of a particular intervention. Nonetheless, the data in the original systematic reviews and the new data found in this update are consistent in terms of direction and consistency of effectiveness.

Another challenge of this research is that there are potentially multiple screening modalities for each cancer site. For example, within CRC screening, the options include FOBT using guaiac-based tests and immunochemical tests, FS, and colonoscopy, as well as other, less common testing modalities. In the opinion of the Panel, the evidentiary base fails to provide comprehensive analyses for each of the potential modalities. Thus, caution is required when interpreting a situation where studies on the same modality yield inconsistent results or studies across modalities appear to favour one screening modality over another. In either case, differences in outcome may be due to issues specific to the modality itself (with some modalities being easier to promote than others) or issues relevant to execution of the study independent of the modality under investigation. These competing hypotheses cannot be teased apart at this time.

There are some clear next steps in the research enterprise related to the science and practice of knowledge translation interventions designed to increase cancer screening rates. They are related to inherent challenges in this literature, and include the failure to provide specific direction and description regarding how interventions are implemented across studies, the lack of consistency regarding how interventions are labelled, and the lack of knowledge regarding the mechanisms underpinning interventions that are responsible for behavior change. For example, while there is evidence in favour of client reminders, the Panel cannot advise on which precise actions are most effective and most likely to yield the greatest impact (*e.g*., by letter versus by call). Advances have been made to guide development, execution, and reporting of some strategies related to the implementation science agenda (*e.g*., AGREE II in the case of practice guidelines http://www.agreetrust.org and the Patient Decision Aids Resource http://decisionaid.ohri.ca/ in the case of patient decision aids). Parallel work would be valuable for other promising interventions.

Similarly, there is a lack of consistency in nomenclature of interventions and the tactics inherent in them. For example, differentiating between letters and invitations is confusing, and both are implicated in both client reminders and small media tactics. With regards to the mechanisms that explain why an intervention may or may not yield change, here too the data are not transparent and the theoretical underpinnings incomplete in the primary studies. For example, small media may have the objective of trying to be persuasive in a context of nudging an individual towards a particular decision, or it may have the objective of providing balanced information in an atmosphere of shared decision making. These distinctions were rarely articulated in the primary studies and the interface between the intervention and the theory behind the strategies rarely addressed. Future studies in this area should more precisely define the features of different interventions to establish the relative effectiveness of each mechanism.

In summary, our systematic review identified reasonable candidate implementation interventions aimed to increase the uptake of breast, cervical, and CRC screening. This systematic review has subsequently been used as the evidentiary foundation of an implementation guideline on this topic.

## Competing interests

The authors declare that they have no competing interests.

## Authors' contributions

MB and CD developed the original study concept and protocol. MB, CD, and LB were responsible for acquisition and analysis of the data; development of the initial draft manuscript, and manuscript revisions. All authors were responsible for the interpretation of the data; review of the draft versions of the manuscript; provision of feedback for important intellectual revisions; and review and final approval of the version to be published.

## Supplementary Material

Additional file 1 **Members of Cancer Screening Uptake Expert Panel**. Click here for file

Additional file 2 **Literature Search Strategies**. Literature search strategies for the update are provided for Medline, EMBASE, CINAHL and PsycINFO. Click here for file

Additional file 3 **AMSTAR assessment of included systematic reviews**. Quality appraisal of the original evidentiary base (systematic reviews) using the AMSTAR tool. Click here for file

Additional file 4 **Formulae for the calculation of percent point (PP) change**. Formulas utilized in percent point change calculations are dependent on the measurements provided in each study. Click here for file

Additional file 5 **Study quality characteristics of included randomized controlled trials for client reminder interventions**. All studies are related to client reminders since no trials were obtained for client incentive interventions. Information on publication status, funding, randomization method, baseline, characteristics, blinding, statistical power, target sample size, follow-up period and intention to treat analysis are provided. Click here for file

Additional file 6 **Randomized controlled trial results: Client Reminders**. All studies are related to client reminders since no trials were obtained for client incentive interventions. Information on participant criteria, study group numbers, intervention descriptions, reporting, and results are provided. Click here for file

Additional file 7 **Study quality characteristics of included randomized controlled trials for small media interventions**. All studies are related to small media since no trials were obtained for mass media interventions. Information on publication status, funding, randomization method, baseline, characteristics, blinding, statistical power, target sample size, follow-up period and intention to treat analysis are provided. Click here for file

Additional file 8 **Randomized controlled trial results: Small Media**. All studies are related to small media since no trials were obtained for mass media interventions. Information on participant criteria, study group numbers, intervention descriptions, reporting, and results are provided. Click here for file

Additional file 9 **Study quality characteristics of included randomized controlled trials for group education and one-on-one education**. Information on publication status, funding, randomization method, baseline, characteristics, blinding, statistical power, target sample size, follow-up period and intention to treat analysis are provided. Click here for file

Additional file 10 **Randomized controlled trial results: Group Education and One-On-One Education**. Information on participant criteria, study group numbers, intervention descriptions, reporting, and results are provided. Click here for file

Additional file 11 **Study quality characteristics of included randomized controlled trials for reducing structural barriers and out-of-pocket expenses**. Information on publication status, funding, randomization method, baseline, characteristics, blinding, statistical power, target sample size, follow-up period and intention to treat analysis are provided. Click here for file

Additional file 12 **Randomized controlled trial results: Reducing Structural Barriers and Out-of-Pocket Expenses**. Information on participant criteria, study group numbers, intervention descriptions, reporting, and results are provided. Click here for file

Additional file 13 **Study quality characteristics of included randomized controlled trials for interventions directed at providers**. All studies are related to provider assessment/feedback since no trials were obtained for provider incentive interventions. Information on publication status, funding, randomization method, baseline, characteristics, blinding, statistical power, target sample size, follow-up period and intention to treat analysis are provided. Click here for file

Additional file 14 **Randomized controlled trial results: Interventions Directed at Providers**. All studies are related to provider assessment/feedback since no trials were obtained for provider incentive interventions. Information on participant criteria, study group numbers, intervention descriptions, reporting, and results are provided. Click here for file
