# Supplementary material for: What implementation interventions increase cancer screening rates? a systematic review
Source: Implement Sci. 2011 Sep 29;6:111. doi: 10.1186/1748-5908-6-111 (PMC3197548; doi:10.1186/1748-5908-6-111)
Supplement: Additional file 1 — Members of Cancer Screening Uptake Expert Panel. [file 1748-5908-6-111-S1.DOC]

**Additional File 1. Members of Cancer Screening Uptake Expert Panel.**

| Dr. Melissa Brouwers, Chair  Provincial Director, Program in Evidence-based Care (PEBC), Cancer Care Ontario  Associate Professor, Department of Oncology and Associate Member, Department of Clinical Epidemiology & Biostatics  McMaster University, Hamilton ON | Dr. Cheryl Levitt  Provincial Clinical Lead, Primary Care  Cancer Care Ontario, Toronto ON |
| --- | --- |
| Dr. Angela Carol  Family Physician  Hamilton Urban Core Community Centre  Hamilton ON | Dr. Nancy Lewis  Senior Policy and  Planning Officer  Cancer Care Ontario, Toronto ON |
| Dr. June Carroll  Associate Professor  Department of Family and Community Medicine  Mount Sinai Hospital  University of Toronto, Toronto ON | Dr. S. Elizabeth McGregor  Research Scientist  Population Health Research,  Alberta Health Services - Cancer Epidemiology, Prevention & Screening  Calgary AB |
| Dr. Michelle Cotterchio  Scientist and Associate Professor  Population Studies and Surveillance  Cancer Care Ontario  and Dalla Lana School of Public Health, University of Toronto, Toronto ON | Dr. Lawrence Paszat  Senior  Scientist, Institute for Clinical Evaluative Sciences  Associate Professor, Dept of Health Policy Management and Evaluation and Dept of Radiation Oncology  University of Toronto, Toronto ON |
| Ms. Carol De Vito  Research Coordinator  PEBC, Cancer Care Ontario  Hamilton, ON | Ms. Carol Rand  Chair, Regional Cancer Prevention and Early Detection Network Hamilton, Niagara, Haldimand, Brant  Director of Systemic, Supportive  and Regional Cancer Programs  Juravinski Cancer Centre, Hamilton ON |
| Dr. Maureen Dobbins  Associate Professor and  Career Scientist, Ontario Ministry of Health & Long-Term Care  School of Nursing, McMaster University  Hamilton ON | Dr. Nadine Wathen Associate Professor  Faculty of Information & Media Studies  University of  Western Ontario, London ON |
| Dr. Barbara Lent  Professor  Department of Family Medicine  University of Western Ontario, London, ON | Ms. Lavannya Bahirathan  Research Assistant  PEBC, Cancer Care Ontario  Hamilton, Ontario |
