# Supplementary material for: What implementation interventions increase cancer screening rates? a systematic review
Source: Implement Sci. 2011 Sep 29;6:111. doi: 10.1186/1748-5908-6-111 (PMC3197548; doi:10.1186/1748-5908-6-111)
Supplement: Additional file 2 — Literature Search Strategies. Literature search strategies for the update are provided for Medline, EMBASE, CINAHL and PsycINFO. [file 1748-5908-6-111-S2.DOC]

**Additional File 2. Update 1: Literature search strategies: 1999 – 2008.**

**Ovid MEDLINE(R) <1996 to July Week 4 2008> Search Strategy:**

- 1. exp mammography/
  2. exp vaginal smears/
  3. exp occult blood/
  4. exp sigmoidoscopy/
  5. exp colonoscopy/
  6. exp prostate-specific antigen/
  7. mammogra*.mp.
  8. (cervical smear or cervical screen*).mp.
  9. (pap smear or pap test*).mp.
  10. (papanicolaou smear or papanicolaou test*).mp.
  11. vagina* smear*.mp. (6591)
  12. (fobt or fecal occult blood test* or faecal occult blood test* or occult blood).mp.
  13. sigmoidoscopy.mp.
  14. colonoscopy.mp.
  15. (prostate-specific antigen* or psa).mp.
  16. exp patient acceptance of health care/
  17. physician-patient relations/
  18. knowledge, attitudes, practice/
  19. persuasive communication/
  20. patient education/
  21. health promotion/
  22. exp patient compliance/
  23. exp patient participation/
  24. (accept* or adher* or apathy or attend* or attitude* or barrier* or behavio* or compli* or comply* or consent* or cooperat* or dropout* or drop* out* or improv* or increas* or incidence or motivat* or nonattend* or non-attend* or nonrespon* or non-respon* or particip* or prev?lence or promot* or refus* or respon* or satisf* or takeup* or uptake or utili*).mp.
  25. (adopt* or alert* or appointment* or audit* or campaign* or community or counsel* or decision aid* or educat* or feedback or home visit* or hotline or invit* or intervention* or letter* or mail* or media or phone or office system prompt* or opinion leader* or questionnaire* or risk factor assessment* or recall or recruit* or reminder* or reminder system* or self-refer* or send or sent or strateg* or telephon* or training or video*).mp.
  26. or/24-25
  27. meta-analysis as topic/
  28. meta analysis.pt.
  29. (meta analy* or metaanaly*).tw.
  30. (systematic review* or pooled analy* or statistical pooling or mathematical pooling or statistical summar* or mathematical summar* or quantitative synthes?s or quantitative overview).tw. (15351)
  31. (systematic adj (review* or overview?)).tw.
  32. (exp review literature as topic/ or review.pt. or exp review/) and systematic.tw. (18923)
  33. or/27-32
  34. (cochrane or embase or psychlit or psyclit or psychinfo or psycinfo or cinahl or cinhal or science citation index or scisearch or bids or sigle or cancerlit).ab.
  35. (reference list* or bibliograph* or hand-search* or relevant journals or manual search*).ab.
  36. (selection criteria or data extraction or quality assessment or jadad scale or methodological quality).ab.
  37. (study adj selection).ab.
  38. 36 or 37 or 34 or 35
  39. review.pt.
  40. 38 and 39
  41. random*.mp. or Randomized controlled trial.pt.
  42. 41 not review.pt.
  43. 33 or 40 or 42
  44. (comment or letter or editorial or note or erratum or short survey or news or newspaper article or patient education handout or case report or historical article).pt.
  45. 43 not 44
  46. or/1-15
  47. or/16-23
  48. 46 and (47 or 26) and 45
  49. limit 48 to (english language and humans and yr="1999 - 2008")
  50. 49 not animal/

**Database: EMBASE <1996 to 2008 Week 32> Search Strategy:**

- 1. cancer screening.mp. or Cancer Screening/
  2. exp Mammography/ or mammogra*.mp.
  3. exp Vagina Smear/
  4. ((cervical or pap* or vagina*) adj (smear* or screen* or test*)).mp.
  5. sigmoidoscopy.mp. or exp SIGMOIDOSCOPY/
  6. colonoscopy.mp. or exp COLONOSCOPY/
  7. (fobt or fecal occult blood test or faecal occult blood test or occult blood).mp. or exp Occult Blood/
  8. (psa or (prostate adj specific adj antigen)).mp. or exp Prostate-Specific Antigen/
  9. 1 or 2 or 3 or 4 or 5 or 6 or 7 or 8
  10. exp Patient Compliance/ or Patient Information/ or Doctor Patient Relation/ or exp Patient Participation/ or Cancer Patient/ or Patient Satisfaction/ or Patient Attitude/
  11. persuasive communication/
  12. Patient Education/
  13. Health Promotion/
  14. 10 or 11 or 12 or 13
  15. (accept* or adher* or apathy or attend* or attitude* or barrier* or behavio* or compli* or comply* or consent* or cooperat* or dropout* or drop* out* or improv* or increas* or incidence or motivat* or nonattend* or non-attend* or nonrespon* or non-respon* or particip* or prev?lence or promot* or refus* or respon* or satisf* or takeup* or uptake or utili*).mp.
  16. (adopt* or alert* or appointment* or audit* or campaign* or community or counsel* or decision aid* or educat* or feedback or home visit* or hotline or invit* or intervention* or letter* or mail* or media or phone or office system prompt* or opinion leader* or questionnaire* or risk factor assessment* or recall or recruit* or reminder* or reminder system* or self-refer* or send or sent or strateg* or telephon* or training or video*).mp.
  17. 15 or 16
  18. exp Meta Analysis/ or exp "Systematic Review"/
  19. (meta analy$ or metaanaly$).tw.
  20. (systematic review$ or pooled analy$ or statistical pooling or mathematical pooling or statistical summar$ or mathematical summar$ or quantitative synthes?s or quantitative overview).tw.
  21. (systematic adj (review$ or overview?)).tw.
  22. exp "Review"/ or review.pt.
  23. (systematic or selection criteria or data extraction or quality assessment or jadad scale or methodological quality).ab.
  24. (study adj selection).ab.
  25. (cochrane or embase or psychlit or psyclit or psychinfo or psycinfo or cinahl or cinhal or science citation index or scisearch or bids or sigle or cancerlit).ab.
  26. (reference list$ or bibliograph$ or hand-search$ or relevant journals or manual search$).ab.
  27. 22 and (23 or 24 or 25 or 26)
  28. random*.mp. or randomized controlled trial.pt.
  29. 28 not review.pt.
  30. (editorial or note or letter erratum or short survey).pt. or abstract report/ or letter/ or case study/
  31. 18 or 19 or 20 or 21 or 27 or 29
  32. 31 not 30
  33. 9 and (14 or 17) and 32
  34. limit 33 to (human and english language and yr="1999 - 2008")

**CINAHL - Cumulative Index to Nursing & Allied Health Literature <1982 to August Week 1 2008> Search Strategy:**

- 1. mammogra*.mp. or exp Mammography/
  2. sigmoidoscopy.mp. or exp SIGMOIDOSCOPY/
  3. colonoscopy.mp. or exp COLONOSCOPY/
  4. ((cervical or pap* or vagina*) adj (smear* or screen* or test*)).mp. or exp Cervical Smears/
  5. (fobt or fecal occult blood test or faecal occult blood test or occult blood).mp. or exp Occult Blood/
  6. (psa or (prostate adj specific adj antigen)).mp. or exp Prostate-Specific Antigen/
  7. exp Cancer Screening/ or cancer screen*.mp.
  8. 1 or 2 or 3 or 4 or 5 or 6 or 7
  9. exp professional-patient relations/
  10. Patient Education/
  11. Health Promotion/
  12. exp Patient Compliance/
  13. consumer participation/ or preventive health care/ or women's health services/
  14. 9 or 10 or 11 or 12 or 13
  15. (accept* or adher* or apathy or attend* or attitude* or barrier* or behavio* or compli* or comply* or consent* or cooperat* or dropout* or drop* out* or improv* or increas* or incidence or motivat* or nonattend* or non-attend* or nonrespon* or non-respon* or particip* or prev?lence or promot* or refus* or respon* or satisf* or takeup* or uptake or utili*).mp.
  16. (adopt* or alert* or appointment* or audit* or campaign* or community or counsel* or decision aid* or educat* or feedback or home visit* or hotline or invit* or intervention* or letter* or mail* or media or phone or office system prompt* or opinion leader* or questionnaire* or risk factor assessment* or recall or recruit* or reminder* or reminder system* or self-refer* or send or sent or strateg* or telephon* or training or video*).mp.
  17. 15 or 16
  18. Meta Analysis/ or (meta-analy* or metaanaly* or (meta adj analy*)).mp.
  19. (systematic review* or pooled analy* or statistical pooling or mathematical pooling or statistical summar* or mathematical summar* or quantitative synthes?s or quantitative overview).mp.
  20. (systematic adj (review* or overview?)).mp.
  21. exp "Literature Review"/
  22. 18 or 19 or 21 or 21
  23. (cochrane or embase or psychlit or psyclit or psychinfo or psycinfo or cinahl or cinhal or science citation index or scisearch or bids or sigle or cancerlit).ab.
  24. (reference list* or bibliograph* or hand-search* or relevant journals or manual search*).ab.
  25. (selection criteria or data extraction or quality assessment or jadad scale or methodological quality).ab.
  26. (study adj selection).ab.
  27. 23 or 24 or 25 or 26
  28. review.pt.
  29. 27 and 28
  30. Clinical Trials/ or random*.mp.
  31. 30 not review.pt.
  32. 22 or 29 or 31
  33. (comment or letter or editorial or note or erratum or short survey or news or newspaper article or patient education handout or case report or historical article).pt.
  34. 32 not 33
  35. 8 and (14 or 17) and 34
  36. limit 35 to english
  37. limit 36 to yr="1999 - 2008"

**Database: PsycINFO <1985 to July Week 5 2008> Search Strategy:**

- 1. exp Cancer Screening/
  2. mammogra*.mp.
  3. ((papanicolaou or pap or cervical or vaginal) adj (smear* or test*)).mp.
  4. (fobt or fecal occult blood test or faecal occult blood test or occult blood).mp.
  5. sigmoidoscopy.mp.
  6. colonoscopy.mp.
  7. ((prostate adj specific adj antigen) or psa).mp.
  8. 1 or 2 or 3 or 4 or 5 or 6 or 7
  9. treatment compliance/ or client attitudes/ or client education/ or client participation/ or treatment barriers/
  10. therapeutic processes/
  11. health attitudes/ or health behavior/ or health knowledge/ or health promotion/
  12. persuasive communication/
  13. public service announcements/ or health promotion/ or mass media/ or prevention/ or public health/ (27954)
  14. 9 or 10 or 11 or 12 or 13
  15. (accept* or adher* or apathy or attend* or attitude* or barrier* or behavio* or compli* or comply* or consent* or cooperat* or dropout* or drop* out* or improv* or increas* or incidence or motivat* or nonattend* or non-attend* or nonrespon* or non-respon* or particip* or prev?lence or promot* or refus* or respon* or satisf* or takeup* or uptake or utili*).mp.
  16. (adopt* or alert* or appointment* or audit* or campaign* or community or counsel* or decision aid* or educat* or feedback or home visit* or hotline or invit* or intervention* or letter* or mail* or media or phone or office system prompt* or opinion leader* or questionnaire* or risk factor assessment* or recall or recruit* or reminder* or reminder system* or self-refer* or send or sent or strateg* or telephon* or training or video*).mp.
  17. 15 or 16
  18. (meta analy* or meta-analy* or metaanaly*).mp.
  19. (systematic review* or pooled analy* or statistical pooling or mathematical pooling or statistical summar* or mathematical summar* or quantitative synthes?s or quantitative overview).mp.
  20. (systematic adj (review* or overview?)).mp.
  21. 18 or 19 or 20
  22. (cochrane or embase or psychlit or psyclit or psychinfo or psycinfo or cinahl or cinhal or science citation index or scisearch or bids or sigle or cancerlit).ab.
  23. (reference list* or bibliograph* or hand-search* or relevant journals or manual search*).ab.
  24. (selection criteria or data extraction or quality assessment or jadad scale or methodological quality).ab.
  25. (study adj selection).ab.
  26. 22 or 23 or 24 or 25
  27. RCT or random*).mp.
  28. 21 or 26 or 27 (1872898)
  29. 8 and (14 or 17) and 28
  30. limit 29 to yr="1999 - 2008"
  31. limit 30 to human

**Appendix 2. Update 2: Literature search strategies continued: 2008 – 2010.**

**Ovid MEDLINE(R) <1996 to May Week 1 2010> Search Strategy:**

1. exp mammography/
2. exp vaginal smears/
3. exp occult blood/
4. exp sigmoidoscopy/
5. exp colonoscopy/
6. exp prostate-specific antigen/
7. mammogra*.mp.
8. (cervical smear or cervical screen*).mp.
9. (pap smear or pap test*).mp.
10. (papanicolaou smear or papanicolaou test*).mp.
11. vagina* smear*.mp.
12. (fobt or fecal occult blood test* or faecal occult blood test* or occult blood).mp.
13. sigmoidoscopy.mp.
14. colonoscopy.mp.
15. (prostate-specific antigen* or psa).mp.
16. exp patient acceptance of health care/
17. physician-patient relations/
18. knowledge, attitudes, practice/
19. persuasive communication/
20. patient education/
21. health promotion/
22. exp patient compliance/
23. exp patient participation/
24. (accept* or adher* or apathy or attend* or attitude* or barrier* or behavio* or compli* or comply* or consent* or cooperat* or dropout* or drop* out* or improv* or increas* or incidence or motivat* or nonattend* or non-attend* or nonrespon* or non-respon* or particip* or prev?lence or promot* or refus* or respon* or satisf* or takeup* or uptake or utili*).mp.
25. (adopt* or alert* or appointment* or audit* or campaign* or community or counsel* or decision aid* or educat* or feedback or home visit* or hotline or invit* or intervention* or letter* or mail* or media or phone or office system prompt* or opinion leader* or questionnaire* or risk factor assessment* or recall or recruit* or reminder* or reminder system* or self-refer* or send or sent or strateg* or telephon* or training or video*).mp.
26. or/24-25
27. meta-analysis as topic/
28. meta analysis.pt.
29. (meta analy* or metaanaly*).tw.
30. (systematic review* or pooled analy* or statistical pooling or mathematical pooling or statistical summar* or mathematical summar* or quantitative synthes?s or quantitative overview).tw.
31. (systematic adj (review* or overview?)).tw.
32. (exp review literature as topic/ or review.pt. or exp review/) and systematic.tw.
33. or/27-32
34. (cochrane or embase or psychlit or psyclit or psychinfo or psycinfo or cinahl or cinhal or science citation index or scisearch or bids or sigle or cancerlit).ab.
35. (reference list* or bibliograph* or hand-search* or relevant journals or manual search*).ab.
36. (selection criteria or data extraction or quality assessment or jadad scale or methodological quality).ab.
37. (study adj selection).ab.
38. 36 or 37 or 34 or 35
39. review.pt.
40. 38 and 39
41. random*.mp. or Randomized controlled trial.pt.
42. 41 not review.pt.
43. 33 or 40 or 42
44. (comment or letter or editorial or note or erratum or short survey or news or newspaper article or patient education handout or case report or historical article).pt.
45. 43 not 44
46. or/1-15
47. or/16-23
48. 46 and (47 or 26) and 45
49. limit 48 to (english language and yr="2008 - 2010")
50. 49 not animal/

**Database: EMBASE <1996 to 2010 Week 20> Search Strategy:**

1. cancer screening.mp. or Cancer Screening/
2. exp Mammography/ or mammogra*.mp.
3. exp Vagina Smear/
4. ((cervical or pap* or vagina*) adj (smear* or screen* or test*)).mp.
5. sigmoidoscopy.mp. or exp SIGMOIDOSCOPY/
6. colonoscopy.mp. or exp COLONOSCOPY/
7. (fobt or fecal occult blood test or faecal occult blood test or occult blood).mp. or exp Occult Blood/
8. (psa or (prostate adj specific adj antigen)).mp. or exp Prostate-Specific Antigen/
9. 1 or 2 or 3 or 4 or 5 or 6 or 7 or 8
10. exp Patient Compliance/ or Patient Information/ or Doctor Patient Relation/ or exp Patient Participation/ or Cancer Patient/ or Patient Satisfaction/ or Patient Attitude/
11. persuasive communication/
12. Patient Education/
13. Health Promotion/
14. 10 or 11 or 12 or 13
15. (accept* or adher* or apathy or attend* or attitude* or barrier* or behavio* or compli* or comply* or consent* or cooperat* or dropout* or drop* out* or improv* or increas* or incidence or motivat* or nonattend* or non-attend* or nonrespon* or non-respon* or particip* or prev?lence or promot* or refus* or respon* or satisf* or takeup* or uptake or utili*).mp.
16. (adopt* or alert* or appointment* or audit* or campaign* or community or counsel* or decision aid* or educat* or feedback or home visit* or hotline or invit* or intervention* or letter* or mail* or media or phone or office system prompt* or opinion leader* or questionnaire* or risk factor assessment* or recall or recruit* or reminder* or reminder system* or self-refer* or send or sent or strateg* or telephon* or training or video*).mp.
17. 15 or 16
18. exp Meta Analysis/ or exp "Systematic Review"/
19. (meta analy$ or metaanaly$).tw.
20. (systematic review$ or pooled analy$ or statistical pooling or mathematical pooling or statistical summar$ or mathematical summar$ or quantitative synthes?s or quantitative overview).tw.
21. (systematic adj (review$ or overview?)).tw.
22. exp "Review"/ or review.pt.
23. (systematic or selection criteria or data extraction or quality assessment or jadad scale or methodological quality).ab.
24. (study adj selection).ab.
25. (cochrane or embase or psychlit or psyclit or psychinfo or psycinfo or cinahl or cinhal or science citation index or scisearch or bids or sigle or cancerlit).ab.
26. (reference list$ or bibliograph$ or hand-search$ or relevant journals or manual search$).ab.
27. 22 and (23 or 24 or 25 or 26)
28. random*.mp. or randomized controlled trial.pt.
29. 28 not review.pt.
30. (editorial or note or letter erratum or short survey).pt. or abstract report/ or letter/ or case study/
31. 18 or 19 or 20 or 21 or 27 or 29
32. 31 not 30
33. 9 and (14 or 17) and 32
34. limit 33 to (human and english language and yr="2008 - 2010")

**Database: PsycINFO <1985 to May Week 1 2010> Search Strategy:**

1. exp Cancer Screening/
2. mammogra*.mp.
3. ((papanicolaou or pap or cervical or vaginal) adj (smear* or test*)).mp.
4. (fobt or fecal occult blood test or faecal occult blood test or occult blood).mp.
5. sigmoidoscopy.mp.
6. colonoscopy.mp.
7. ((prostate adj specific adj antigen) or psa).mp.
8. 1 or 2 or 3 or 4 or 5 or 6 or 7
9. treatment compliance/ or client attitudes/ or client education/ or client participation/ or treatment barriers/
10. therapeutic processes/
11. health attitudes/ or health behavior/ or health knowledge/ or health promotion/
12. persuasive communication/
13. public service announcements/ or health promotion/ or mass media/ or prevention/ or public health/
14. 9 or 10 or 11 or 12 or 13
15. (accept* or adher* or apathy or attend* or attitude* or barrier* or behavio* or compli* or comply* or consent* or cooperat* or dropout* or drop* out* or improv* or increas* or incidence or motivat* or nonattend* or non-attend* or nonrespon* or non-respon* or particip* or prev?lence or promot* or refus* or respon* or satisf* or takeup* or uptake or utili*).mp.
16. (adopt* or alert* or appointment* or audit* or campaign* or community or counsel* or decision aid* or educat* or feedback or home visit* or hotline or invit* or intervention* or letter* or mail* or media or phone or office system prompt* or opinion leader* or questionnaire* or risk factor assessment* or recall or recruit* or reminder* or reminder system* or self-refer* or send or sent or strateg* or telephon* or training or video*).mp.
17. 15 or 16
18. (meta analy* or meta-analy* or metaanaly*).mp.
19. (systematic review* or pooled analy* or statistical pooling or mathematical pooling or statistical summar* or mathematical summar* or quantitative synthes?s or quantitative overview).mp.
20. (systematic adj (review* or overview?)).mp.
21. 18 or 19 or 20
22. (cochrane or embase or psychlit or psyclit or psychinfo or psycinfo or cinahl or cinhal or science citation index or scisearch or bids or sigle or cancerlit).ab.
23. (reference list* or bibliograph* or hand-search* or relevant journals or manual search*).ab.
24. (selection criteria or data extraction or quality assessment or jadad scale or methodological quality).ab.
25. (study adj selection).ab.
26. 22 or 23 or 24 or 25
27. (RCT or random*).mp.
28. 21 or 26 or 27
29. 8 and (14 or 17) and 28
30. limit 29 to yr="2008 - 2010"
31. limit 30 to human
