# Supplementary material for: What implementation interventions increase cancer screening rates? a systematic review
Source: Implement Sci. 2011 Sep 29;6:111. doi: 10.1186/1748-5908-6-111 (PMC3197548; doi:10.1186/1748-5908-6-111)
Supplement: Additional file 3 — AMSTAR assessment of included systematic reviews. Quality appraisal of the original evidentiary base (systematic reviews) using the AMSTAR tool. [file 1748-5908-6-111-S3.DOC]

**Additional File 3. AMSTAR assessment of included systematic reviews [11].**

| **Publication (Reference)**  **AMSTAR Item** | **Baron 2008 (8)** | **Baron 2008 (9)** | **Sabatino 2008 (10)** |
| --- | --- | --- | --- |
| 1. **A priori design** | Y | Y | Y |
| 1. **Duplicate study selection** | Y | Y | Y |
| 1. **Comprehensive literature search performed** | Y | Y | Y |
| 1. **Status of publication in inclusion criteria** | Y | Y | Y |
| 1. **List of studies included and excluded** | N | N | N |
| 1. **Characteristic of included studies provided** | N | N | N |
| 1. **Scientific quality of included studies assessed & documented** | Y | Y | Y |
| 1. **Scientific quality of included studies used appropriately** | Y | Y | Y |
| 1. **Methods used to combine findings appropriate?** | Y | Y | Y |
| 1. **Likelihood of publication bias assessed** | N | N | N |
| 1. **Conflict of interest stated** | C | C | C |

Abbreviations: AMSTAR, Assessment of Multiple Systematic Reviews; C = Cannot answer; N = No; NA = Not Applicable; Y = Yes.
