# Supplementary material for: What implementation interventions increase cancer screening rates? a systematic review
Source: Implement Sci. 2011 Sep 29;6:111. doi: 10.1186/1748-5908-6-111 (PMC3197548; doi:10.1186/1748-5908-6-111)
Supplement: Additional file 4 — Formulae for the calculation of percent point (PP) change. Formulas utilized in percent point change calculations are dependent on the measurements provided in each study. [file 1748-5908-6-111-S4.DOC]

**Additional File 4. Formulae for the calculation of percentage point (PP) change.**

For studies with before-and-after measurements and concurrent comparison groups:

PP = (Ipost -Ipre) - (Cpost - Cpre); baseline = Ipre

For studies with post-only measurements and concurrent comparison groups:

PP = (Ipost - Cpost); baseline = Cpost

For studies with before-and-after measurements and no concurrent comparison groups:

PP = (Ipost - Ipre); baseline = Ipre

Note: This formula was used in the original systematic reviews only.

Where:

Ipost = reported percentage of intervention group screened after intervention;

Ipre = reported percentage of intervention group screened, immediately before intervention;

Cpost = reported percentage of comparison group screened after intervention;

Cpre = reported percentage of comparison group screened, immediately before intervention; and

Baseline = the estimated study population screening rate in the absence of or prior to intervention.

Post-intervention results given in included studies as a percentage (relative) change from baseline or as odds ratios (ORs) that could not be converted to PP absolute changes were reported separately. Each included study determined screening completion by either client self-report or record reviews. Absolute rates of overall effectiveness (i.e., across studies) between the control arm and the intervention arm were not provided in the systematic reviews.
