# Supplementary material for: What implementation interventions increase cancer screening rates? a systematic review
Source: Implement Sci. 2011 Sep 29;6:111. doi: 10.1186/1748-5908-6-111 (PMC3197548; doi:10.1186/1748-5908-6-111)
Supplement: Additional file 5 — Study quality characteristics of included randomized controlled trials for client reminder interventions. All studies are related to client reminders since no trials were obtained for client incentive interventions. Information on publication status, funding, randomization method, baseline, characteristics, blinding, statistical power, target sample size, follow-up period and intention to treat analysis are provided. [file 1748-5908-6-111-S5.DOC]

**Additional File 5. Study quality characteristics of included randomized controlled trials for client reminder interventions. (No trials were obtained for client incentive interventions.)**

| **Study** | **Publication status** | **Funding** | **Randomization method** | **Baseline characteristics** | **Blinding** | **Statistical Power** | **Achievement of Target Sample Size** | **Follow-up** | **Intention-to-Treat (ITT) analysis** |
| --- | --- | --- | --- | --- | --- | --- | --- | --- | --- |
| **Interventions targeting the public to increase demand for screening** | | | | | | | | | |
| ***Client Reminders: Breast Cancer*** | | | | | | | | | |
| **Non-clustered** | | | | | | | | | |
| Beach et al., 2007 [16] | Full publication | NCI | NR | NR | NR | NR | NR | Intervention group followed 18 mos or until up-to-date (2001-2004) | Yes; 44 pts dropped from analyses: 4 Creole speakers and 40 with history of cancer were excluded |
| Champion et al, 2007 [15] | Full publication | NINR | NR | Balanced | NR | NR | NR | At 4 mos | NR |
| Chaudhry et al., 2007 [18] | Full publication | Mayo Foundation | NR | NR | NR | 1,955 pts needed for 80% power with p=.05. | Yes | At 12 mos; 518 randomized did not participate (did not consent to participate) | Only randomized pts who consented to participate analyzed |
| Dietrich et al., 2006 [17] | Full publication | NCI | Sealed randomization forms; computer-based random-number generator | Balanced | Reviewers blinded to study hypotheses and to group assignment | Assumed proportion  of women screened differed by 0.1 for each 3 primary tests with power of 0.8; to correct for multiple comparisons, type I error of 0.0167 (0.05/3); assumed withdrawal rate 20% & needed sample size =1400 | Yes | At 18 mos | Yes; 18% of intervention group lost to f/u |
| Allen et al., 2005 [19] | Full publication | University of California Breast Cancer Research Program | NR | Balanced | NR | NR | NR | At 6 mos | Yes; 76 lost to f/u |
| DeFrank et al., 2009 [21] | Full publication | NCI & AHRQ | Eligible participants pre-randomized to a group with larger proportions allocated to 2/3 intervention arms | Data collected; no comparisons made | NR | 3545 pts needed to provide 80% power to detect a 6% difference in intervention arms; two- tailed tests with alpha 0.05 | Yes | At 1, 2, 3, and 4 yrs | Yes; Analysis of all randomized pts minus 220 (excluded) |
| Goel et al., 2009 [20] | Full publication | ACS | Women randomized to either care (stratified by age and race) | Balanced | NR | NR | NR | At 2 mos | NR |
| ***Client Reminders: Cervical Cancer*** | | | | | | | | | |
| **Clustered** | | | | | | | | | |
| Jensen et al., 2009 [23] | Full publication | NR | Block randomization at the physician level according to practice numbers | Data from practices collected; balanced | GPs and researchers not blinded | NR | NR | At 5, 8 & 11 mos | NR |
| **Non-clustered** | | | | | | | | | |
| Beach et al., 2007 [16] | See *Client Reminders: Breast Cancer* |  |  |  |  |  |  |  |  |
| Dietrich et al., 2006 [17] | See *Client Reminders: Breast Cancer* |  |  |  |  |  |  |  |  |
| Morrell et al., 2005 [22] | Full publication | NR | NR | Balanced | NR | α=5%, power=80% | Yes | At 90 days | Analysis included all randomized participants |
| ***Client Reminders: Colorectal Cancer*** | | | | | | | | | |
| **Clustered** | | | | | | | | | |
| Walsh et al., 2005 [25] | Full publication | ACS & UCSF | Block randomization of physicians, stratified by group size | Physicians: more of intervention group in academic setting  Patients: balanced | NR | Type I error=0.05; 80% power to detect effect differences | NR | At 1, 2, and 5 yrs, depending on group | NR |
| Nease et al., 2008 [28] | Full publication | NCI & AHRQ | Physicians clustered under practice  Practices randomly grouped into 3 arms; 1 arm unable to follow protocol and reassigned | Practice data collected; no comparisons made | Practices were not blinded | NR | NR | At 9 mos | NR |
| Potter et al., 2009 [29] | Full publication | ACS | Randomized by practice | Unbalanced | NR | NR | NR | At 9 mos | NR |
| **Non-clustered** | | | | | | | | | |
| Christie et al., 2008 [26] | Full publication | NCI | Randomized using random-number-generating program | NR | NR | NR | NR | NR | NR |
| Beach et al., 2007 [16] | See *Client Reminders: Breast Cancer* |  |  |  |  |  |  |  |  |
| Myers et al., 2007 [24] | Full publication | NIH/NCI | NR | NR | Providers of participants blinded to study group assignment | 95% power to detect differences on order of 10% age points; 5% Type I error | NR | At 12 & 24 mos | Yes |
| Denberg et al., 2006 [27] | Full publication | ACS | Random-number generator, then random and sequential assignment to group | Balanced | Blinded manual review of claims data unnecessary | Target sample size 792 pts (or 396 in each group) with 80% power and alpha value set at 0.05. | Yes | At 4 mos | Yes |
| Dietrich et al., 2006 [17] | See *Client Reminders: Breast Cancer* |  |  |  |  |  |  |  |  |
| Chan et al., 2008 [32] | Full publication | NCI & AHRQ | Separate sets of sealed envelopes used to randomly assign participants | Unbalanced between public and private access groups  Control and intervention arms unbalanced for education only | Allocation staff blinded to patient arm | Pilot study with small sample size, hence significance testing not conducted | No | At 2 or 3 mos depending on group | NR |
| Lee et al., 2009 [30] | Full publication | VA San Diego Healthcare System | Patients sequentially assigned to either arm using a random-number generator | Balanced | Chart reviewers blinded to randomization and return FOBT cards | Target sample size 792 pts to detect 10% difference with 80% power and alpha value set at 0.05 (two-tailed) | No; sample size for analysis = 769 pts | At 6 mos | Yes |
| Potter et al., 2009 [31] | Full Publication | ACS & NCI (AANCART) | 9/17 randomly selected clinic sessions in blocks of 2 or 3 execute intervention | Unbalanced; intervention group more likely to be younger and have lower income | Clinic staff and patients blinded to intervention day | NR | NR | At 3 and 6 wks | NR |

Notes: AANCART, Asian American Network for Cancer Awareness, Research and Training; ACS, American Cancer Society; AHRQ, Agency for Health Research and Quality; F/U, follow-up; GP(s), general practitioner(s); ITT, intention-to-treat; mos, months; NCI, National Cancer Institute; NIH, National Institutes of Health; NINR, National Institute of Nursing Research; NR, not reported; pts, patients; UCSF, University of California, San Francisco; yr(s), year(s).
