# Supplementary material for: What implementation interventions increase cancer screening rates? a systematic review
Source: Implement Sci. 2011 Sep 29;6:111. doi: 10.1186/1748-5908-6-111 (PMC3197548; doi:10.1186/1748-5908-6-111)
Supplement: Additional file 6 — Randomized controlled trial results: Client Reminders. All studies are related to client reminders since no trials were obtained for client incentive interventions. Information on participant criteria, study group numbers, intervention descriptions, reporting, and results are provided. [file 1748-5908-6-111-S6.DOC]

**Additional File 6. Randomized controlled trial results: Client Reminders**

| **Article**  **(References)** | **Population**  **Description** | **Study Group**  **Numbers** | **Grouping & Description** | **Reporting** | **Results*** |
| --- | --- | --- | --- | --- | --- |
| ***Client Reminders: Breast Cancer*** | | | | | |
| **Non-clustered** | | | | | |
| Beach et al.,  2007 [16]  US – NYC  **Breast, cervical, & colorectal screening** | 50 – 69 y  Not up-to-date for screening for at least one study cancer  Urban  Spanish 848  English 498  Low SES  Accrual: 2001-2004 | 1346  Control 676  Intervn 670 | Baseline interview  Control group: Usual care  Prevention Care Manager (PCM) group  In language of choice (English or Spanish) periodic reminder telephone calls from PCM to help overcome barriers, provide emotional support & schedule appts + mailed educational materials & reminders | Medical records | PCM intervention resulted in higher mammography screening adherence vs. control group at 18 mos follow-up:  I 68% - C 57% = +11.0 PPI  OR _adj_ =1.59; 95% CI (1.27- 2.00);  p≤.001, corrected χ^2^  In all the subgroups, Spanish speakers scored higher vs. English speakers  **I vs. C**  Spanish: I 72% - C 58% = +14.0 PPI  OR=1.83; 95% CI (1.38-2.44); p≤.001, corrected χ^2^  English: I 60% - C 56% = +4.0 PPI  OR= 1.21; 95% CI (0.85-1.73) p≤.001, corrected χ^2^ |
| Champion et al., 2007 [15]  US– St Louis, MO & Indianapolis, IN | ≥50 y  Non-adherent 15 mo prior  Urban  Cost covered  African-American 54%  Caucasian 44%  Low SES | 1244  Group 1 294  Group 2 314  Group 3 329  Group 4 308 | Initial study description letter & brochure  Group 1/Comparison = Usual care  Group 2 = Tailored telephone intervention - verbal delivery of same information as for Group 3 + answering specific questions (about tailored information or about cost)  Group 3 = Tailored print intervention – physician-signed cover letter + 1-3 page newsletter  Group 4 = Tailored print & phone counselling | Electronic medical records | Intervention groups showed greater adherence than control:  Group OR 95% CI LRTp  4 1.9 1.30-2.83 .001  3 1.7 1.16-2.53 .006  2 1.6 1.07-2.38 .021  Marital status, employment, race, income, & age had no significant effects on adherence  Group %screened PPI  4 35 12.0  3 32 9.0  2 29 6.0  1 23 |
| Chaudhry et al.,  2007 [18]  US – Rochester, MN | 40-75 y  Due for test  Urban | 6665  Control 3339  Intervn 3326 | Control group  Usual care with mammography; either part of office visit or requested by patients  Intervention  Personalized reminder letter + brochure  No response at 1 mo🡪 letter or e-mail  No response at 2 mos🡪 scripted telephone call | Web-based patient reminder tracking system (PRECARE) | Annual mammography screening rate improved in favour of intervention group:  I 64.3% - C 55.3% = +9.0 PP increase, p<.001 |
| Dietrich et al., 2006 [17]  US – New York City, NY  **Breast, cervical, & colorectal screening** | 50-69 y  Overdue  English  Spanish  Haitian  Known ethnicity:  Black 38%  White 39%  Urban  Low SES  Accrual:  2001-2004 | 1390 (initially 1413)  Control 694  Intervn 696 | All received Initial preventive services guide  Usual Care group/Comparison  Single telephone call to answer preventive care questions, inform of status, advise to obtain such care from primary care clinician  Intervention group  Tailored telephone reminder & support calls + Print reminders from trained prevention care managers over 18 mo or until patient up to date for screening – combined with educational material, barrier identification & reduction/access advice | Medical charts | Telephone-based intervention increased mammography screening  Intervention increased:  10.0 PP (95% CI 0.05%, 0.15%)  or 17% (p<.001)  Control decreased:  -0.02 PP (95% CI -0.08%, 0.02%)    I 10.0 – C -0.02 = +10.0 PPI |
| Allen et al.,  2005 [19]  US – Los Angeles, CA | ≥ 40 y  Non-adherent 1 y prior  African-American 38.1%  Hispanic 44.9%  Other 17.0%  Inner city  Low SES 47%<$US20000 | 430  Compar 211  Intervn 219 | Community-based survey questionnaire prior to participants enrolment  Comparison group: No intervention  Intervention group  Tailored scripted telephone call (Spanish or English) with screening information, appointment scheduling, & barrier counselling. Reminder letter with screening shower cards and brochures  All participants  Follow-up questionnaire and telephone interview at 6 mo to confirm if mammogram done or not | Self-report | No statistically significant difference found between intervention and control group mammogram screening rate:  I 36.8% - C 29.0% = 7.8 PPI |
| DeFrank et al.,  2009 [21] US – North Carolina | 40 – 75 y  SHP holders with previous screening conducted a year ago and due for their next mammogram  White 87.9%  Black 10.6%  Accrual: 2004-2005 | 3327  (initially 3547)  EUCR 799  ATR 1259  ELR 1269  Allocation of patients in larger proportions to ATR and ELR for future analyses EUR 25.0% ATR 37.5% ELR 37.5% | Baseline telephone interview  EUCR: Enhanced Usual Care Reminders  Mailed letters, including dates of last mammogram, benefits of mammography, recommendations and SHP coverage  ATR: Automated Telephone Reminders  Same content as EUCR but delivered as an automated telephone call using a real woman’s voice  ELR: Enhanced Letter Reminders  Mailing contained additional information in a coloured 4 page booklet including the severity of breast cancer, susceptibility and contact information to their previous screening facility | Self-report and health claims data | ATR intervention resulted in higher repeat screening adherence Group %Screened PPI  EUCR 71.8  ATR 76.3 4.5  ELR 74.5 2.7  Group AOR 95% CI p-value  EUCR ref  ATR 1.32 1.06, 1.64 .014  ELR 1.19 0.96, 1.48 .117  (adjusted for demographic variables)  White women, those aged 50-75 y, those reporting no financial hardship, and those reporting excellent or good health were more likely to have been adherent to repeat mammography.  Overall, 74.5% were adherent to repeat mammography screening post-intervention compared to 56.7% prior to intervention delivery, resulting in an absolute increase of 17.8% |
| Goel et al.,  2009 [20]  US– Wayne County (Detroit), MI | 40 – 64 y  Uninsured or underinsured  Eligible, but not already enrolled in the WCBCCCP  African-American 77.5% White 22.5%  Low SES | 1209  Control 610  Intervn 599 | Usual Care group/Control  Postcard mailed out one year after the last recorded WCBCCCP enrolment to encourage re-enrolment and re-screening  Intervention group  Usual care plus telephone reminder  The WCBCCCP removes financial and health care access barriers facing economically disadvantaged women Re-screening defined as completing either a CBE and/or a mammogram | WCBCCCP records | Annual mammography screening rate improved in favour of intervention group at 6 mos f/u:  I 31% - C 23% = 8.0 PPI, p=.004    60-day re-screening intervn data AOR=1.91; 95% CI (1.37-2.65)  Among the subgroup of women who re-enrolled within 180 days 60-day re-screening intervn data  AOR=0.72; 95% CI (0.37-1.40) |
| ***Client Reminders: Cervical Cancer*** | | | | | |
| **Clustered** | | | | | |
| Jensen et al.,  2009 [23]  Denmark - Aarhus | 23-59 y  Overdue for a Pap smear test | 117129   Control 59183  Intervn 57946 | Unit of randomization: GP  All eligible women received a normal invitation letter to the screening program  Control Group: Usual care  Intervention Group  The GP’s received a visit from a facilitator to provide quality enhancements to the cervical screening program and offered to contact non-attenders by mailing out a special targeted letter personally signed by the GP emphasizing reasons for screening | Medical database | The intervention effectively and consistently increased the proportion of women participating in cervical screening;  PPI 95% CI  3mos +0.70% (0.13%-1.28%) 6mos +0.94% (0.21%-1.67%)  9mos +1.97% (0.03%-3.91%) trend test: p<0.036  Screening rates 1.17 (95%CI: 1.04-1.30) times higher for intervention group than control group  (adjusted for GPs characteristics and proportion of non-attenders) |
| **Non-clustered** | | | | | |
| Beach et al.,  2007 [16]  US – NYC  **Breast, cervical, & colorectal screening** | For details, see Client Reminders: Breast Cancer | 967  Control 476  Intervn 491 | For details, see Client Reminders: Breast Cancer | Medical records | PCM intervention resulted in higher cervical screening adherence vs. control group  I 70% - C 57% = +13.0 PP increase  OR_adj_=1.73; 95% CI (1.31-2.27); p≤.001, corrected χ^2^  In all the subgroups, Spanish speakers scored higher vs. English speakers  I vs. C  Spanish: 76% - 60% = +16.0 PPI  OR=2.18; 95% CI (1.53-3.10); p≤.001, corrected χ^2^  English: 59% - 53% = +6.0 PPI  OR=1.23; 95% CI (0.81-1.86)  Statistically significant interaction between language & study group for intervention impact among Spanish speakers:  OR=1.77; 95% CI (1.03-3.05); p≤.05 |
| Dietrich et al., 2006 [17]  US – NYC  **Breast, cervical, & colorectal screening** | For details, see Client Reminders: Breast Cancer | 1390  (initially 1413)  Control 694  Intervn 696 | For details, see Client Reminders: Breast Cancer | Medical charts | Telephone-based intervention increased cervical screening rates  Intervention increased:  7% (95% CI 0.03%, 0.11%)  or 10% (p<.001)  Control unchanged:  0% (95% CI -0.03%, 0.05%)  I 7% – C 0% = +7.0 PP increase |
| Morrell et al.,  2005 [22]  Australia – NSW | 20-69 y  Overdue for screening >48 mos  Low – high SES  Urban & rural  Accrual: 2002-2003 | 90,000  Control 30,000  Intervn 60,000 | Control group: no intervention  Intervention group  Either of 2 personally addressed reminder letters | NSW Pat Test Register | At 90-day follow-up, significantly higher Pap test rate (p<.05) for intervention group over control group:  I 4.44% – C 2.90% = +1.54 PPI |
| ***Client Reminders: Colorectal Cancer*** | | | | | |
| **Clustered** | | | | | |
| Walsh et al.,  2005 [25]  US – San Francisco CA | 50-79 y  Non-adherent for 2 y prior  Caucasian 59%  African-American 10%  Hispanic 12%  Asian 19%  Accrual:  FOBT 2000-2002  FS/Col 2002-2005 | 7993  (any test)  Control 3717  Intervn 4276 | Unit of randomization: Physician  Control group: Usual care  Intervention group  Physician-signed personal reminder letter + educational brochure + FOBT kit + stamped return envelope  Unclear whether this cluster trial adjusted for design effect. | Medical charts | At 2 y (n=7993):  No difference in CRC screening rates between intervention and control groups for any screening test:  I 12.7% - C 12.5% = +0.2 PPI, p=.51  FOBT screening rate increase greater in control group vs. intervention:  C 13.1 – I 11.4 = +1.7 PPI, p=.05  At 5 y (n=2665):  FS percentage increase greater in intervention group vs. control group:  I 7.4% - C 4.4% = +3.0 PPI, p<.01  No significant increase (p=.46) in colonoscopy rates:  I 9.5% - C 8.9% = +0.6 PPI |
| Nease et al.,  2008 [28]  US – Michigan | ≥ 50 y  Non-adherent for CRC screening based on US Preventative Task Force recommendations  Primarily rural  Accrual: 2003 - 2005 | 12 practices  (initially 13)  (any CRC test)  Clinicians 4  Patients 4  Both 4  Patient only arm reassigned to clinician only arm mid-way through trial | Unit of randomization: Practice  Intervention  Computerized reminder system set in place to deliver reminder forms to clinicians only, patients only, or both patients and clinicians | Medical records | Improvement in screening rates across all 12 practices averaged a 9 PPI, from 41.7% at baseline to 50.9% post intervention (p=0.002, range -9% to 24% PPD). All but one practice improved.  No significant differences or trends seen in the screening rates between practices in the original randomization or the clinician versus patient and clinician reminder groups that practices moved into |
| Potter et al.,  2009 [29] US – San Francisco, CA | >50 y Female 62% White 41%  Asian 32% African- American 12%  Doctor’s appt during 6 mos intervention Accrual: 2003 – 2007 | 5 practices (any CRC test) Usual Care 1 Poster Only 2 Poster/Phone 2 | Unit of randomization: Primary Care Practices  Control group (C) – No intervention  Poster Only arm (P)  A large colourful multilingual (English, Chinese, Russian, Spanish, Tagalog) poster presenting options for CRC screening in each exam room  Poster/ Phone Reminder arm (P+P)  Poster intervention plus follow-up telephone call 2-4 wks after CRC screening ordered to remind the patient and discuss concerns  Cluster trial did not adjust for design effect; unit of analysis error | Medical records | Both intervention arms show a modest statistically significant increase in up to date CRC screening status  % Screened Pre- Post- %Diff p PPI C 54.6% 57.1% +2.5 .185 -  P 55.4% 58.9% +3.5 .009 1.0  P+P 61.9% 65.9% +4.0 <.001 1.5  At baseline, 59.7% of patients adherent to CRC screening recommendations. As a result of the study, several patients in the subset due for screening received testing;  Control 19.2%  Poster Only 22.1% OR=1.04;p=.147 Poster+Phone 28.1% OR=1.49;p=.001 |
| **Non-clustered** | | | | | |
| Christie et al.,  2008 [26]  US – NYC | 49-70 y  Physician-referred for Col  Female 75%  African-American 21%  Hispanic 71%  Low SES  Accrual: 2004 | 21 (Colonoscopy)  PN- 8  PN+ 13 | PN-/Control - Usual care group  Physician referral 🡪 GI scheduler in 1^st^ wk telephoned pt to schedule screening & discuss procedure & questions, mailed prescriptions and instructions 🡪 endoscopy unit phoned pt 2 days prior to confirm appt  PN+/Intervention group  Physician referral 🡪PN phoned pt in 1^st^ wk (and GI scheduler) to make screening appt; phoned pt to confirm appt, explain procedure and preparation, answer questions or refer pt; mailed prescriptions & instructions; phoned reminder 1 wk prior, explained preparation & answered questions; phoned 2 days prior to confirm appt & preparation & address any concerns; phoned 2 days after to confirm screening done and if not, to discuss how to alleviate barriers & reschedule appt | Medical records | 53.8% of intervention group completed Col screening vs. 13% (n=1) of control group (p=.058)  53.8% - 13% = 40.8 PP increase  23% of intervention group refused screening vs. 63% of control group |
| Beach et al.,  2007 [16]  US – NYC  **Breast, cervical, & colorectal screening** | For details, see Client Reminders: Breast Cancer | 1070  (any test)  Control 542  Intervn 528 | For details, see Client Reminders: Breast Cancer | Medical records | PCM intervention resulted in higher CRC screening adherence vs. control group  53% - 38% = 15.0 PP increase  OR_adj_=1.92; 95% CI 1.49, 2.47; p≤.001, corrected χ^2^  In all the subgroups, Spanish speakers scored higher vs. English speakers  I vs. C  Spanish: 54% - 37% =17.0 PPI  OR=2.01; 95% CI, 1.48-2.73;  p≤.001, corrected χ^2^  English: 50% - 39% = 11.0 PPI  OR=1.58; 95% CI, 1.06-2.36; p≤.001, corrected χ^2^  Overall, FOBT return rate in PCM intervention group higher for Spanish speakers vs. English:  OR=2.12, 95% CI 1.53-2.96 vs. OR=1.40; 95% CI, 0.90-2.17 |
| Myers et al.,  2007 [24]  US – Philadelphia PA  **(IFIT used, not guaiac-based FOBT)** | 50-74 y  Non-adherent for CRC screening at least 1 y prior  Female 67%  African-American 58%  Accrual: 2002-2004 | 1546  (SBT, FS, & Col)  Control 387  SI 387  TI 386  TIP 386 | All participants  Baseline survey  Midpoint survey at 12 mo  Endpoint survey at 24 mo  Control group: Usual care  Intervention groups  SI— mailed standard intervention (CRC screening invitation letter encouraging SBT [IFIT] return + informational booklet + SBT + reminder letter)  TI— standard intervention + 2 tailored message pages addressing personal barriers to SBT & FS  TIP— standard intervention + tailored message pages + reminder telephone call by trained health educator | Chart audit, billing & lab databases, self-report | All interventions groups showed significantly higher CRC screening uptake than control group:  SI 46% - C 33% = 13.0 PPI  TI 44% - C 33% = 11.0 PPI  TIP 48% - C 33% = 15.0 PPI  Group OR 95% CI p-val  SI 1.7 1.25-2.53 .001  TI 1.6 1.18-2.12 .002  TIP 1.9 1.42-2.56 <.001  Screening uptake did not differ significantly across intervention groups  TIP group members who received reminder call were more likely to be screened (52%) than members who did not receive call (35%):  OR, 2.0; 95% CI (1.2–3.1)  Screening use significantly higher for TIP group participants who received reminder call vs. control group (OR, 2.3; 95% CI, 1.7-3.2), SI group (OR, 1.4; 95% CI, 1.0-1.9), and TI group (OR, 1.5; 95% CI, 1.1-2.0) |
| Denberg et al.,  2006 [27]  US – Denver | ≥ 50 y  Physician referral for Col  Female 62%  White 57%  African-American 9%  Latino 4%  Low/Med/High SES  Urban  Accrual: 2005 | 781 (Colonoscopy)  Control 395  Intervn 386 | Control group  Usual care—physician’s referral with written instructions to schedule appt for CRC screening  Intervention group  Personalized educational brochure (1-page, 2-sided with eighth-grade level text) about CRC cancer and Col procedure (description, risks) and including reminder & instructions for scheduling appt | Hospital claims records | Intervention group Col completion rate 11.7 PPI (95% CI, 5.1%-18.4%) greater than control group:  I 70.7% vs. C 59.0%; p=.001  Multivariate results:  Intervention group 20% more likely (RR, 1.20; 95% CI 1.09, 1.33) and participants ≥ 65 y 18% (RR, 1.18; 95% CI 1.04, 1.34) more likely to complete screening |
| Dietrich et al.,  2006 [17]  US – NYC  **Breast, cervical, & colorectal screening** | For details, see Client Reminders: Breast Cancer | 1390 (initially 1413)  (any test)  Control 694  Intervn 696 | For details, see Client Reminders: Breast Cancer | Medical charts | Telephone-based intervention increased CRC screening rates:  Intervention increased significantly:  0.24% PP (95% CI 0.20%, 0.29%)  or 63% (p<.001)  Control decreased:  0.11% PP (95% CI 0.08%, 0.16%)  I 24% - C 11% = +13.0 PP increase |
| Chan et al.,  2008 [32]  US – Houston, TX | ≥ 50 y  Due for CRC screening  No prior history of colorectal cancer/surgery Email and internet access  Female 59%  African-American 42%  White 49%  Accrual:  2004 – 2005 | 97  (FOBT)  Private access, 77 pts eligible  Control 35   Intervn 42  Public access, 20 pts eligible  Control 9  Intervn 11 | Private versus Public access  Patients able to access the internet and email either at home/work (private) or at a library (public) for the intervention arms  Control  Personalized letter from their physician, a reminder and FOBT kits. A second reminder sent 2 wks later and a F/U survey mailed 2 & 3 mos after enrolment.  Intervention  Patients received a NetLET email reminder (personalized letter from physicians, educational webpage links, test results from prior CRC screening) and mailed a FOBT kit. NetLET reminders sent twice at weekly intervals. F/U survey sent out 2 & 3 mos after.  All participants  Given $US20 coupon upon completing the enrolment survey and sent a $55 money order upon receipt of the F/U survey | Return of FOBT | Private access arm I 26% - C 23% = +3.0 PP increase  Public access arm I 0% - C 33% = -33.0 PP increase  Researchers unable to address system and access barriers. As a result, it is not feasible to implement the NetLET intervention |
| Lee et al.,  2009 [30] US – San Diego, CA | ≥ 50 y  Physician referral for Col  Male 96%  White 73%  Black 12%  Accrual: 2007 | 769 (initially 775) (FOBT)  Control 382  Intervn 387 | Control group  Usual care—physician’s referral for FOBT, patients instructed to pick up FOBT cards and return them to the lab for analysis  Intervention group  Usual care + educational reminder (1-page, eighth-grade level text) about returning the FOBT card, CRC risk, benefits of screening, quote from CRC survivor and 24/7 contact information | Medical record database | Significantly higher odds of FOBT card return in intervention arm:  I 64.6% - C 48.4% = +16.2 PPI  OR**_Adj_**, 2.02; 95% CI (1.48 – 2.74); p<0.001; (adjusted for multivariate logistic regression)  This represents a 33.5% increase in FOBT card return rate |
| Potter et al.,  2009 [31]  US- San Francisco, CA | 50-79 y  Due for CRC screening  Low SES Ethnic diversity  Accrual: 2006 | 514  (FOBT)  Control 246  Intervn 268 (153 eligible for FOBT kit; 90 completed) | All participants  Mailed multilingual health campaign notice to encourage a clinic visit for the flu shot  Control group: Usual Care  Intervention group  Received CRC prevention educational sheet and eligible patients given a FOBT kit with postage paid return envelopes. Telephone reminder calls made at 3 and 6 wks. | Medical records and return of FOBT | The colorectal cancer screening rate dramatically improved in favour of intervention  % Screened   Pre- Post- %Diff p-val C 52.9% 57.3% +4.4% p=.07 **I** 54.5% 84.3% +29.8% p<.001  I 29.8% - C 4.4% = +25.4 PPI  OR, 11.3; 95% CI (5.8-22.0); p<.001 |

NOTES: (un)adj, (un)adjusted; appt(s), appointment(s); C, control group; CBE, clinical breast examination; CI, confidence interval; Col, colonoscopy; Compar, comparison group; CRC, colorectal cancer; d, day; diff, difference; FOBT, fecal occult blood test; FS, flexible sigmoidoscopy; F/U, follow-up; GI, gastroenterology; GP(s), general practitioner(s); IFIT, InSure Fecal Immunochemical Test; Intervn or I, intervention group; LRT, likelihood ratio test(s); M, men; mam, mammogram(s)/phy; mo, month(s); NCI CIS, National Cancer Institute Cancer Information Service; NSW, New South Wales (AUS); PCM, Prevention Care Management (US); P, poster group; P+P, poster + phone group; P-P, per-protocol; Pap, Papanicolau; PP. Percentage point; PPI, percentage point increase; pt(s), patient(s); (A)OR, (adjusted) odds ratio; RR, relative risk ratio; SBT, stool blood test; SES, socioeconomic status(es); SHP, State Health Plan; SI, standard intervention; ST, standard intervention; TI, tailored intervention; TIP, tailored intervention + telephone reminder; US, United States; vs., versus; W, women; WCBCCCP, Wayne County Breast and Cervical Cancer Control Program; wk(s), week(s); y, year(s).

* If data were available in a report and the percentage point (PP) increase was not already reported, the PP increase was calculated and included in the Results column.
