# Supplementary material for: What implementation interventions increase cancer screening rates? a systematic review
Source: Implement Sci. 2011 Sep 29;6:111. doi: 10.1186/1748-5908-6-111 (PMC3197548; doi:10.1186/1748-5908-6-111)
Supplement: Additional file 7 — Study quality characteristics of included randomized controlled trials for small media interventions. All studies are related to small media since no trials were obtained for mass media interventions. Information on publication status, funding, randomization method, baseline, characteristics, blinding, statistical power, target sample size, follow-up period and intention to treat analysis are provided. [file 1748-5908-6-111-S7.DOC]

**Additional File 7. Study quality characteristics of included randomized controlled trials for small media intervention. (No trials were obtained for mass media interventions.)**

| **Study** | **Publication status** | **Funding** | **Randomization method** | **Baseline characteristics** | **Blinding** | **Statistical Power** | **Achievement of Target Sample Size** | **Follow-up** | **Intention-to-Treat (ITT) analysis** |
| --- | --- | --- | --- | --- | --- | --- | --- | --- | --- |
| **Interventions targeting the public to increase demand for screening** | | | | | | | | | |
| ***Small Media: Breast Cancer*** | | | | | | | | | |
| **Clustered** | | | | | | | | | |
| Abood et al.,  2005 [34] | Full publication | ACS | NR | More white participants & more reporting breast cancer symptoms  in comparison  group | NR | 82% power to detect difference of 12% between the two groups with n=112 in experimental and n=992 in control | Yes | At 6 mos. | Yes, analysis included all randomized participants |
| Michielutte et al., 2005 [35] | Full publication | NCI | NR | Balanced | NR | NR | NR | At 4 mos, 9 mos, & 12 mos | No; 11% attrition rate for complete f/u |
| Bodurtha et al., 2009 [38] | Full publication | NCI | Biostatistician prepared clinic stratified block randomization assignments before the study | Balanced | NR | Target sample size 900 pts with 80% power to detect a 10% difference assuming 10% attrition; alpha value set at 0.05 | Yes | At 1, 6 & 18 mos | Yes; data available for 84% of subjects |
| **Non-clustered** | | | | | | | | | |
| Vernon et al., 2008 [37] | Full publication | NIH | Computer-generated random-number assignment | Balanced | Staff members conducting mailings & telephone f/u blind to intervention group status | 80% power and a  two-sided test of statistical significance | Yes | At year 1 or year 2 | Yes; also modified ITT and per-protocol |
| Page et al.,  2006 [33] | Full publication | NR | Computer-generated random-number assignment, then consecutive assignment to 1 of 4 groups | Balanced | NR | Sample size calculation based on detecting  hypothesised differences of 5% and 7% in screening rates for each intervention, with 80% power & 95% confidence level | Yes | At 12 wks | Yes |
| DeFrank et al., 2009 [20] | Full publication | NCI & AHRQ | Eligible participants pre-randomized to a group with larger proportions allocated to 2/3 intervention arms | Data collected; no comparisons made | NR | 3545 pts needed to provide 80% power to detect a 6% difference in intervention arms; two- tailed tests with alpha 0.05 | Yes | At 1, 2, 3, and 4 yrs | Yes; Analysis of all randomized pts minus 220 (excluded) |
| Russell et al., 2010 [36] | Full publication | NIH/NCI & Indiana University School of Nursing/ CEQLCI | Stratified random assignment into 3 age groups using a computer generated list | Low-dose comparison group more likely to have insurance | Data collectors blinded; assistants and participants  not blinded | NR | Yes | At 6 mos | Yes |
| ***Small Media: Cervical Cancer*** | | | | | | | | | |
| **Non-clustered** | | | | | | | | | |
| Corkrey et al., 2005 [39] | Full publication | Hunter Medical  Research Inst. & University of Newcastle & Hunter Centre for Health Advancement | Principal components analysis to randomize by socioeconomic status and postal code, then assigned by alternate pairs to intervention or control | NR | NR | NR | NR | At 6 mos | NR |
| Hou et al.,  2005 [40] | Full publication | Cheng-Chin Hospital, Taiwan | NR | Balanced | NR | NR | NR | At 3 mos | NR |
| Stein et al.,  2005 [41] | Full publication | NEDHA, SWDHA | NR | Balanced | Allocation Unit staff blinded to participant identity | 80% power and 95% precision; n=219 participants needed per group | Yes | Within 3 mos | Yes; 304 lost to f/u: 193 excluded by GP, 111 not contactable |
| ***Small Media: Colorectal Cancer*** | | | | | | | | | |
| **Clustered** | | | | | | | | | |
| Fitzgibbon et al., 2007 [43] | Full publication | NR | Randomized by primary care clinic (n=2: usual care=1, intervention=1) | Balanced | NR | NR | NR | Every 4-6 mos over 2 yrs | NR |
| Manne et al., 2009 [49] | Full publication | NIH | Patients randomized by family unit | Data collected; no comparisons made | NR | NR | NR | At 6 - 8 mos | Yes; if screening status not collected, pt considered non-compliant |
| Potter et al.,  2009 [29] | Full publication | ACS | Randomized by practice | Unbalanced | NR | NR | NR | At 9 mos | NR |
| Sequist et al., 2009 [48] | Full publication | NCI | One individual from the patient pairs randomly assigned to intervention arm | Balanced | NR | NR | NR | At 15 mos | NR |
| Stephens et al., 2007 [46] | Full Publication | NR | Patients clustered by family and randomised as a unit to either arm via sealed envelopes | Balanced | Allocation staff blinded to patient care | Target size of 100 subjects in each arm for a power of 90%, alpha = 0.05 | No | At 3 mos | NR |
| **Non-clustered** | | | | | | | | | |
| Rawl et al.,  2008 [45] | Full publication | NCI | Computerized randomization | Balanced | NR | NR | NR | At 3 mos | No; 68 not analyzed: 16 lost due to attrition; 52 excluded from analysis—15 already had test, 37 underage |
| Cole et al.,  2007 [51] | Full publication | NHMRC, Australia | NR | NR | NR | NR | NR | At 12 wks | NR |
| Ruffin et al.,  2007 [44] | Full publication | NCI & MDCH | Computerized block randomization  process & two strata, race and gender | Balanced | Investigators,  data collectors, data entry, and data analyst all blinded to study arm assignment; participants blind to study arms | Power 80%, alpha 0.05, intervention effect size  estimated at 20% | Yes | At 24 wks | Yes, analysis included all randomized participants |
| Marcus et al., 2005 [47] | Full publication | NR | NR | NR | NR | NR | NR | At 6 & 12 mos | Yes; pts lost to f/u NR |
| Miller et al.,  2005 [50] | Full publication | Cancer Center Core Grant, Comprehensive Cancer Center, Wake Forest University | Pts randomized with equal probability using permuted blocks | Balanced | Telephone contact RA or investigator completing questionnaire blinded to randomization scheme | Initial sample n=400 allowed detection of adherence rate differences of ≥15% with 80% power. Revised sample n=200 allowed detection of rate differences of ≥20% with 80% power | Yes | At 30 days | 10 excluded: 2 refusals, 2 had work-up, 2 already tested, 4 <50 yrs; analysis only of included pts |
| Zapka et al.,  2004 [42] | Full publication | NIH | Computer-generated random-number table assignment | Balanced | Interviewer blinded to group assignment at baseline and F/u | Power 80%, alpha=.05 | Yes | At 6 mos | Yes |
| Gimeno-Garcia et al., 2009 [52] | Full publication | Instituto de Salud Carlos III, Consejeria de Educacion, and Caja de Canarias | Patients (stratified for age and gender) randomized to receive intervention according to clinic visit day | Balanced | Gerontologists not blinded; Assistants blinded to intervention day | A sample size of 79 participants per group yields 80% power to detect a 15% difference; one-tailed test, alpha= 0.05 | Yes | At 2 wks | All randomized patients included in the analysis |
| Potter et al.,  2009 [32] | Full Publication | ACS & NCI (AANCART) | 9/17 randomly selected clinic sessions in blocks of 2 or 3 execute the intervention | Unbalanced; intervention group more likely to be younger and have lower income | Clinic staff and patients blinded to intervention day | NR | NR | At 3 and 6 wks | NR |

Notes: AANCART, Asian American Network for Cancer Awareness, Research and Training; ACS, American Cancer Society; AHRQ, Agency for Health Research and Quality; CEQLCI, Centre for Enhancing Quality of Life in Chronic Illness; f/u, follow-up; GP(s), general practitioner(s); ITT, intention-to-treat; MDCH, Michigan Department of Community Health; mos, months; NCI, National Cancer Institute; NEDHA, North and East Devon Health Authority. England; NHMRC, National Health and Medical Research Council; NIH, National Institutes of Health; NR, not reported; pts, patients; SWDHA, South and West Devon Health Authority, England; wks, weeks; yr(s), year(s).
