# Supplementary material for: What implementation interventions increase cancer screening rates? a systematic review
Source: Implement Sci. 2011 Sep 29;6:111. doi: 10.1186/1748-5908-6-111 (PMC3197548; doi:10.1186/1748-5908-6-111)
Supplement: Additional file 8 — Randomized controlled trial results: Small Media. All studies are related to small media since no trials were obtained for mass media interventions. Information on participant criteria, study group numbers, intervention descriptions, reporting, and results are provided. [file 1748-5908-6-111-S8.DOC]

**Additional File 8. Randomized controlled trials results: Small Media**

| **Article**  **(References)** | **Population**  **Description** | **Study Group**  **Numbers** | **Grouping & Description** | **Reporting** | **Results*** |
| --- | --- | --- | --- | --- | --- |
| ***Small Media: Breast Cancer*** | | | | | |
| **Clustered** | | | | | |
| Abood et al.,  2005 [34]  US – Florida | 50-64 y  White 88%  African-American 12%  Urban clinics  Under/uninsured  Free screening | 1104  Compar 992  Intervn 112 | Unit of randomization: urban catchment area phone centres (unpredictable variation in service demand)  Control group  Standard telephone message to encourage obtaining mammogram  Intervention group  Trained staff asked screening eligibility questions, offered & scheduled appt, and delivered scripted loss-framed educational information message  Unclear whether this cluster trial adjusted for design effect. | NR | Significantly higher odds of mammogram in intervention arm:  Rate  I 27.7% - C 15.8%= +11.9 PPI  OR**_Unadj_**, 2.035; p=.0019; 95% CI (1.30-3.19)  OR**_Adj_**, 1.914; p=.0063; 95% CI (1.20-3.05) (adjusted for race & breast cancer symptoms) |
| Michielutte et al.,  2005 [35]  US - NC  3-stage study of provider (practice) & patients | ≥ 65 y  White 93%  Urban/Rural  Primary group overdue ≥ 15 mos  Maintenance group reported mam in previous year | 2147  Primary  Control 689  Intervn 799  Maintenance  Control 333  Intervn 326 | Unit of randomization: Health care practice  Control group consisted of patients of practices not receiving intervention materials for provider or patient  Intervention group  Stage 1 - pamphlet from practitioner  Stage 2 – 4 mos post-Stage 1 🡪 mailed educational material (pamphlet & fact  sheet), letter from study coordinator encouraging screening  Stage 3 – 4 mos post-Stage 2 🡪brief telephone counselling  Maintenance group used to assess effectiveness of intervention on repeat mammograms | Chart review | Stage 3 telephone counselling was not an effective intervention  Stage 2 (mailed educational materials) showed small significant difference favouring intervention group overall and favouring intervention group within primary group:  %Screened  I C PPI p  Total 14.4% 10.2% +4.2 .020  Primary 13.0% 8.6% +4.4 .026  Maint 17.7% 13.3% + 4.4 .148  Larger percentage of Stage 1 and 2 underserved women (over 80, or black, or < 9 y education, or with no private coverage) received mammograms |
| Bodurtha et al.,  2009 [38] US - VA | ≥ 40 y Not pregnant No history of breast cancer  Urban African 45% | 899  Compar 450  Intervn 449 | Unit of randomization: clinic  Baseline Survey  Control group  General information about breast cancer prevention  Intervention group  Information sheets containing personal 5-year and lifetime risks, HBM (barriers, seriousness and benefits) factors, lifestyle recommendations and instructions for scheduling mammogram/genetic counselling visits | Medical records and self-report | The intervention group faired lower than the control group in improving mammography uptake  % Screened   Pre- Post- %Diff C 64.0% 75.1% +11.1%  **I** 64.1% 73.1% +9.0%   I 9.0% - C 11.1%= -2.1 PP increase OR 0.89; 95% CI (0.65-1.22)  No significant difference between control and intervention groups (p_adj_=.4621). The study was associated with improved rates in women most worried about breast cancer I 85.0% vs. C 63.5%;  OR_adj_=3.06; 95% CI (1.22-7.68) |
| **Non-clustered** | | | | | |
| Vernon et al.,  2008 [37]  US | Veterans  ≥ 52 y | ITT 5500  Group 1 1803  Group 2 1857  Group 3 1840  MITT 3126  Group 1 1034  Group 2 1052  Group 3 1040  P-P 2681  Group 1 886  Group 2 907  Group 3 888 | All participants:  Baseline survey questionnaire  Follow-up: Survey at 1 and 2 y  Group 1/Control: Baseline survey  Intervention groups  Group 2 = Survey + targeted letter  Group 3 = Survey + tailored + targeted letter  Targeted intervention  Folder of 4 educational booklets, letter for use in screening discussion,  pamphlet about VA mammography services  Tailored intervention  Letter (4 page) & 3 of 14 bookmarks personalized to individual’s baseline  survey response | Self-report post-intervention:  Coverage =  1 mam  Compliance =  2 mam | Tailored plus targeted intervention did NOT result in higher mammography  rates than targeted-only intervention  Limited evidence for either intervention being more  effective than baseline survey alone |
| Page et al.,  2006 [33]  Australia | 50-54 y  Never had mammogram with BSNSW  White 98.6%  Rural/urban  All SES | 3144  Group 1 788  Group 2 786  Group 3 785  Group 4 785 | Group 1 = no intervention  Intervention groups  Group 2/One letter = standard practice BSNSW personalized letter  Group 3/Two letters = personalized letter + reminder letter at 6 wks  Group 4/One letter + phone call = personalized letter + reminder call at 6 wks | BSNSW attendance records | Higher screening rates for all 3 intervention groups vs. non-intervention group at 12 weeks  (I - C) %Screened OR p PPI  1 1.4  2 5.5 1.00 4.1  3 8.5 1.61 .05 7.1  4 7.8 1.46 6.4 4x 13.3 .65 .001 11.9 |
| DeFrank et al.,  2009 [20] US – North Carolina | 40 – 75 y  SHP holders with previous screening conducted a year ago and due for their next mammogram  White 87.9%  Black 10.6%  Accrual: 2004-2005 | 3327  (initially 3547)  EUCR 799  ATR 1259  ELR 1269  Allocation of patients in larger proportions to ATR and ELR for future analyses EUR 25% ATR 37.5% ELR 37.5% | Baseline telephone interview  EUCR: Enhanced Usual Care Reminders  Mailed letters, including dates of last mammogram, benefits of mammography, recommendations and SHP coverage  ATR: Automated Telephone Reminders  Same content as EUCR but delivered as an automated telephone call using a real woman’s voice  ELR: Enhanced Letter Reminders  Mailing contained additional information in a coloured 4 page booklet including the severity of breast cancer, susceptibility and contact information to their previous screening facility | Self-report and health claims data | ATR intervention resulted in higher repeat screening adherence  Group %screened PPI  EUCR 71.8  ATR 76.3 4.5  ELR 74.5 2.7  Group AOR 95% CI p  EUCR ref  ATR 1.32 (1.06-1.64) .014  ELR 1.19 (0.96,-1.48) .117  (adjusted for demographic variables)  White women, those aged 50-75 y, those reporting no financial hardship, and those reporting excellent or good health were more likely to have been adherent to repeat mammography.  Overall, 74.5% were adherent to repeat mammography screening post-intervention compared to 56.7% prior to intervention delivery, resulting in an absolute increase of 17.8% |
| Russell et al., 2010 [36] US – Indianapolis, IN | 40-75 y African American  ≤250% federal poverty level No mammogram within last 15 mos No breast cancer history  Urban Accrual: 2006 – 2008 | 181  Compar 90  Intervn 91 | All participants  Baseline and 6 mos survey  Given $25 gift certificate to a local business  Low-Dose: Comparison group  Cultural pamphlet about breast cancer and LHA recommendations to schedule a mammography. 4 nutritional postcards at monthly intervals  Combined Intervention group  Tailored computer assessment at baseline and 4 monthly LHA sessions. LHA offered access enhancing services, including referral to low-cost mammograms, assistance with scheduling screening appointments and help for those requiring transportation. | Medical records and self-report | The combined intervention improved mammography screening rates in low income African-American women  I 50.6% - C 17.8%= +32.8 PPI OR= 4.7; 95% CI (2.4-9.4); p<0.0001 RR_adj_= 2.7; 95% CI (1.8-3.7); p<0.0001  (adjusted for employment status, disability, insurance, 1^st^ degree relatives and previous breast biopsies) |
| ***Small Media: Cervical Cancer*** | | | | | |
| **Non-clustered** | | | | | |
| Corkrey et al.,  2005 [39]  Australia – Hunter region, NSW | 18-69 y  Urban/rural  Accrual: 2001 | Control postal codes 15  Intervn postal codes 15  Intervn phone calls 17,008 | Control group (15 randomly selected postal codes)  No telephone calls or letters  Intervention group (Interactive voice response [IVR] population health intervention)  IVR-information letter + instruction sheet mailed 1 wk prior to call  IVR telephone call (educational component) | Government database | Intervention seen as ‘feasible’ for increasing screening rates  At 3 mo, overall increase of 0.43% screens in intervention postal code areas compared to control postal code areas  Greatest rate increase seen in 50-69 y group at 6 mos—group more at risk for cervical cancer and with lower screening rates:  1.35 PPI, 95% CI (1.28-1.42) |
| Hou et al.,  2005 [40]  Taiwan | ≥ 30 y or < 30 y if married  Non-adherent 1 y prior  Chinese  Accrual: 2000 | 424  Control 212  Intervn 212 | Control group  3 monthly newsletters on general health information  Intervention group  1^st^ mo—personally addressed welcome letter + educational brochure + screening schedule with health hotline number  2^nd^ mo—personalized invitation letter + role model stories & testimonials + cervical cancer & screening fact sheet + updated screening schedule  3^rd^ mo—telephone call to check if screened + counselling + appt arrangement offer  All participants  Small gift on completion of follow-up survey | Self-report & follow-up mailed survey | Intervention group more likely to obtain cervical smear than control group:  OR=2.29; p=.002  Significantly higher screening completion for contemplators rather than pre-contemplators:  OR=4.41; p=.001 |
| Stein et al.,  2005 [41]  UK – Devon | 39 -64 y  Non-adherent ≥ 15 y  Accrual: 2001 | 1140  Control 285  Intervn:  Group 1 222/285  Group 2 219/285  Group 3 221/285 | Control group: Standard invitation letter  Intervention  Group 1—scripted telephone call from nurse  Group 2—letter from Health Authority district Cervical Screening Commissioner (public health doctor)  Group 3—Letter from well-know celebrity | Health authority database | No significant difference between intervention groups and control  Small, non-significant percentage increase in screening uptake with letter from public health doctor (Group 2):  Control 1.8% 95%CI (.57-4.0)  Group 1 1.4% 95%CI (.38-3.6)  Group 2 4.6% 95%CI (2.5-7.7) PPI 2.8  Group 3 1.8% 95%CI (.57-4.0)  Telephone intervention most expensive & least effective |
| ***Small Media: Colorectal Cancer*** | | | | | |
| **Clustered** | | | | | |
| Fitzgibbon et al.,  2007 [43]  US – Chicago | ≥ 50 y  Non-adherent  Male veterans  White 47%  African- American 49%  Accrual: 2-year study | 986  (any tests-FOBT, FS, & Col)  Control 728  Intervn 258 | 2 VA clinics randomized as control & intervention setting  Examined provider intervention & patient intervention  Control group/control clinic: usual care  Intervention group/intervention clinic  Viewed 8-minute motivational & educational video targeted at low literacy + low-literacy CRC screening pamphlet + simplified FOBT instructions | NR | No significant difference between intervention and control groups for completing any screening:  C I  FOBT 30% (218) 26.4% (68) p=.30  FS/Col 18.8% (137) 17.4% (45) p=.71  ALL 41.6% (303) 39.5% (102) p=.61 |
| Manne et al., 2009 [49] US | ≥35 y or ≤10 y younger than the sibling’s diagnosis age Full sibling living with CRC, diagnosed since 1997 and no family history of hereditary cancers English-speaking Female 60% Caucasian 91% Accrual: Dec 2003 – July 2007 | 412  (Colonoscopy)  GP 139  TP 161 TP+TC 112 | Unit of randomization: family  All participants  Baseline survey and $US15 gift certificate for each assessment  Generic Print (GP) group  Cover letter and generic pamphlet reviewing basic CRC information  Tailored Print (TP) group  Personalized letter and tailored booklet (4 pages, coloured, images) discussing specific gender and ethnicity risks for CRC, barriers and cancer stages. F/u newsletter mailed in 1 mo.  Tailored Print + Telephone Counselling (TP+TC)  TP + personalized letter + motivational telephone counselling session 1 wk after enrolment to discuss perceptions, support services and CRC screening recommendations | Self-report | Screening adherence between groups shows a significant tailored intervention effect  %Screened PPI p  GP 13.7%  TP 24.8% +11.1% 0.013 TP+TC 25.9% +12.2% 0.036  The combined data from the 2 TP arms create a reconfigured group with a combined treatment effect 2.12 times higher than the control group  No difference in screening adherence between groups in the tailored condition. A single telephone counselling session did not add significantly to treatment effects. |
| Potter et al.,  2009 [29] US – San Francisco, CA | >50 y Female 62% White 41%  Asian 32% African-American 12% Doctor’s appointment during 6 mo intervention Accrual: 2003–2007 | 5 practices (any CRC test) Control 1 Poster 2 Poster/Call 2 | Unit of randomization: Primary Care Practices  Control group (C) – No intervn  Poster Only arm (P)  A large colourful multilingual (English, Chinese, Russian, Spanish, Tagalog) poster presenting options for CRC screening in each exam room  Poster/Phone Reminder arm (P+P)  Poster intervention plus follow-up telephone call 2-4 wks after CRC screening ordered to remind the patient and discuss concerns  Cluster trial did not adjust for design effect; unit of analysis error | Medical records | Both intervention arms show a modest statistically significant increase in up to date CRC screening status  %Screened  Pre- Post- %Diff p PPI C 54.6 57.1 2.5 .185 -P 55.4 58.9 3.5 .009 +1.0  P+P 61.9 65.9 4.0 .001 +1.5  At baseline, 59.7% of patients adherent to CRC screening recommendations. As a result of the study, several patients in the subset due for screening received testing;  Usual Care 19.2%  Poster Only 22.1% OR 1.04; p=0.147 Poster/Phone 28.1% OR 1.49; p=0.001 |
| Stephens et al., 2007 [46] AUS – Adelaide, South Australia | 1^st^-degree relatives  > 18 y Female 57% English-speaking  Accrual: 2000–2001 | 91  (any test- FOBT, FS & Col)  Control 59  Intervn 32 | Control/Standard Care  Information provided to index patients by the treating surgeon regarding risk  associated with family CRC.  Intervention  Information pamphlet sent to patient’s relatives 1 wk after operation regarding  CRC risks, benefits to screening, cues and barriers. | Self- Report | This targeted intervention was not effective in influencing screening activity  %Screened  I C PPI X^2^  p-value  6% 8% -2% 1.19 0.91 |
| Sequist et al.,  2009 [48]  US – Massachusetts | 50-80 y Overdue for CRC screening Female 57% Study conducted: April 2006- June 2007 | 21860  (any test- FOBT, FS & Col)  Control 10930  Intervn 10930 | Control group: Usual care  Intervention group  Patients received a mailing with 4 components during the 1^st^ mo of intervention and a second mailing 6 mos later  (1) letter from HVMA indicating the patient is due for screening  (2) educational pamphlet of screening options  (3) FOBT kit + stamped return envelope  (4) telephone number to schedule FS or Col | Medical record database | Subgroup analysis of screening rates higher for patients who received intervention mailings compared with control group  Rate PPI(95% CI) p-value I 44.0% +5.8(4.5-7.1) <0.001 C 38.1%  The intervention effect increased with age; (p=0.1 for trend) 50-59 y +3.7% PPI 60-69 y +7.3% PPI  70-80 y +10.1% PPI  The mailing primarily increased the performance of FOBT among intervention patients  %Screened  I C Diff p-value  FOBT 25.4 20.4 5.1% <0.001  FS 0.1 0.1 0.0% 0.66 COL 18.4 17.4 0.7% 0.17  Sequist et al implemented separate interventions directed at either patients or physicians. A comparison between the two intervention groups found a small, negative and non statistically significant value. OR 0.6%; 95%CI (-1.2%-1.1%); p=0.08 |
| **Non-clustered** | | | | | |
| Rawl et al.,  2008 [45]  US | 1^st^-degree relatives  > 40 y  Female 69%  Nonadherent with ACS CRC screening guidelines  Caucasian 77%  African-American 18%  Low/Mid/High SES  ($US<20,000->75,000) | 140  (any tests-FOBT, FS, & Col)  Compar 61  Tailored 79 | Baseline telephone interview  Nontailored group - comparison  ACS brochure on colon testing mailed at 1 mo post-baseline  Tailored group  Customized 10-page print booklet unique to each participant & including personally relevant educational cancer information, message on overcoming personal top 3 barriers, personal risk profile, & appropriate screening test recommendation  All participants  F/U interview 3 mos post-intervn | Self-report | No significant difference between groups on overall adherence  3-mo adherence to any test (FOBT, FS, Col) was slightly lower for tailored intervention group (14.3%  increase) vs. nontailored comparison group (21.3% increase) (p=.30)  14.3% - 21.3% = -7.0 PPI  ACS CRC brochure seen as moderately effective and inexpensive intervention |
| Cole et al.,  2007 [51]  Australia  **IFIT Testing** | 50-74 y  Accrual: 2005 | 2400  Control 600  Risk 600  Advocacy 600  Advance 600 | All participants  Standard invitation letter + IFIT kit+ reply paid envelope  Control  Standard invitation letter about CRC prevention & screening  Risk  Standard invitation letter + positively framed messages about CRC risk  Advocacy  Standard invitation letter + messages from lay advocates encouraging CRC screening  Advance Notification  Advance Notification letter 🡪 standard invitation letter 2 wks later | IFIT return | Screening participation:  Control 39.5%  Risk 40.3% 40.3 – 39.5 = +0.8 PPI  Advocacy 36.0% 36.0 – 39.5 = -3.5 PPI  Advance 48.3% 48.3 – 39.5 = +8.8 PPI  Advance Notification group had significant increase in screening rate compared to control group at wk 12:  +8.8 PPI; RR, 1.23; 95% CI (1.06, 1.43)  No difference between Risk & Advocacy groups and Control |
| Ruffin et al.,  2007 [44]  US-Detroit, Flint/Saginaw, St.Joseph/Benton Harbor MI | 50-70 y  Non-adherent for CRC screening  Women 55%  White 53%  African-American 47%  Suburban, urban, & rural  Accrual: 2002-2003 | 174  (any tests-FOBT, FS, & Col)  Control 87  Intervn 87 | All participants  Pre-session personal history questionnaire  Post-session questionnaire  Follow-up telephone interviews at 2, 8 and 24 wk or until screened  Post-completion $US20.00 honorarium  Control group  Viewed standard CRC website—non-interactive, primarily text, no comparison among CRC screening options  Intervention group  Viewed Colorectal Web—interactive, limited text, high use of graphics & videos, educational objective presentation of CRC tests—participants select CRC test of choice | Self-report | Intervention group significantly more likely to get screened than control arm (p=.035, 2-tailed), with no difference as to type of CRC screening obtained  OR, 3.23; 95%CI, 2.73-3.50  I 64% - C 38% = 26.0 PP increase  CRC screening test obtained by choice:  I C  FOBT 48% 49%  FS 18% 15%  Col 34% 36% |
| Marcus et al.,  2005 [47]  US – 9 NCI CIS 1-800 call centres | ≥ 50 y  Eligible for CRC screening during 1 y follow-up period  Did not request CRC screening information  Female 83%  White 85%  50-59 y 54%  English-speaking | 4014  (any tests-FOBT, FS, & Col) | All participating CSI callers:  Standard brief educational message (BEM)  Baseline interview by trained CIS staff  6 mo follow-up telephone interview  14 mo follow-up telephone interview  Group 1 – SU (control)  BEM + single untailored (SU) NCI booklet  Group 2 - ST  BEM + single 16-page tailored (ST) booklet  Group 3 - MT  BEM + 4 tailored print communication (TPC) mailouts: ST booklet; 6 mos & 9 mos – 4-page newsletter; 12 mos 2-page newsletter  Group 4 – MRT  BEM + 4 retailored TPC (retailoring based on 6 mo interview) mailouts | Self-report | At 6 mo, 75% of participants remained non-adherent for CRC screening  CRC screening adherence at 14 mo follow-up:  significant trend across groups moving from baseline through groups:  Base SU ST MT MRT  0.20 0.42 0.44 0.51 0.48  Trend: 0.03 p=.05  Significant increase shown only for MT vs. SU (9.0 PP increase, p=0.03) |
| Miller et al.,  2005 [50]  US | ≥ 50 y  Physician offered non-diagnostic FOBT  English-speaking  Female 60%  Black 72%  35% adherent for screening  Accrual: 2001-2002 | 194  (FOBT)  Counselling 101  Computer 93 | All participants:  FOBT kit with instructions & return envelope  Telephoned post-intervention questionnaire on CRC knowledge & screening attitude  Nurse Counselling Group: usual care  One-on-one session with office nurse to teach FOBT procedure & answer questions  Educational Computer Program Group  Brief computer-use instructions & then privately viewed multimedia education & information program—animation, photographs, audio clips, & digital vide—on CRC & on FOBT screening procedure  Reminder letter if FOBT cards not returned after 30 d | Medical records | No significant difference found in return rates for FOBT screening for either group:  30 d follow-up  63% of usual care nurse counselling group and 62% of computer group returned FOBT cards = 1.0 PP difference favouring usual care over intervention  95% CI, -15%, +13%; p=.89  Post-30 d  8 patients in nurse counselling group and 3 in computer group returned FOBT cards later (after reminder letter)  No significant difference in return rates (p=.42)  More likely to return card:  Females vs. males (71% vs. 51%, p=.006)  Prior CRC screening history none (79% vs. 54%, p<.001) |
| Zapka et al.,  2004 [42]  US – Massachusetts | 50-74 y  Eligible for FS  White  Urban/ suburban/rural | 938  Control 488  Intervn 450 | All participants  Baseline telephone survey  Follow-up telephone survey at 4-6 mos.  Control: did not receive video  Intervention  Mailed 15-minute educational video encouraging pt to discuss CRC screening, particularly FS, with own physician at next appointment + letter from physician advocating viewing video | Self-report | Intervention had no effect on overall CRC screening rate (55% for both groups); increased FS screening rate only if participant watched video:  I% C% OR(95%CI) PPI  FS 26.2 21.3 1.22(0.88-1.70) +4.9  Watched video:  FS with/ 38.9 21.3 2.81(1.85-4.26) +17.6  without |
| Gimeno-Garcia et al., 2009 [52]  SPAIN - Tenerife | 50-79 y  No individual/  family history of CRC  Overdue for screening  Male 25%  Urban 85%  Accrual: 2007 | 158  (FOBT)  Control 79  Intervn 79 | All participants  Baseline questionnaire  After watching video, participants met with the gastroenterologist and received FOBT kits  Control group  Short non-medical video documentary  Intervention group  3.5 min education video regarding CRC prevention including information about risk factors, prognosis, symptoms, advantages to screening and available procedures | Return of FOBT | Significa nt increase in screening rates for intervention group versus control:  I 69.6% - C 54.4% = 15.2 PP increase, OR=2.0; 95% CI (1.02-3.84); p=0.044  Similar FOBT return rates seen among those not enrolled in the study (52.1%) and the control group (54.4%)  6.6 individuals are required to watch the educational video to increase FOBT-return rates by 1 |
| Potter et al.,  2009 [32]  US- San Francisco, CA | 50-79 y  Due for CRC screening  Low SES Ethnic diversity  Accrual: Sept - Dec 2006 | 514  (FOBT)  Control 246  Intervn 268 (153 eligible for FOBT kit; 90 completed) | All participants  Mailed multilingual health campaign notice to encourage a clinic visit for the flu shot  Control group: Usual Care  Intervention group  Received CRC prevention educational sheet and eligible patients given a FOBT kit with postage paid return envelopes. Telephone reminder calls made at 3 and 6 wks. | Medical records and return of FOBT | The colorectal cancer screening rate dramatically improved in favour of intervention  % Screened   Pre- Post- %Diff p-value C 52.9 57.3 +4.4 0.07 I 54.5 84.3 +29.8 <0.001  I 29.8% - C 4.4% = +25.4 PP increase  OR=11.3; 95%CI (5.8-22.0); p<0.001 |

NOTES: ACS, American Cancer Society; (un)adj, (un)adjusted; appt, appointment; BSNSW, Breast Screen New South Wales; C, control group; CI, confidence interval; Col, colonoscopy; Compar, comparison group; Compl, compliance; CRC, colorectal cancer; d, days; FOBT, fecal occult blood test; FS, flexible sigmoidoscopy; f/u, follow-up’ GP, general practitioner; HBM, Health Belief Model; HVMA, Harvard Vanguard Medical Associates; I, intervention group; IFIT, InSure Fecal Immunochemical Test; Intervn, intervention group; ITT, intention to treat; LHA, lay health advisor; M, men; maint; maintenance; mam, mammogram(s)/phy; min, minutes; mo, month(s); MIT, modified ITT; NC, North Carolina; NCI CIS, National Cancer Institute Cancer Information Service; NSW, New South Wales (AUS); %Diff, percent difference; P, poster; P+P, poster + phone; P-P, per-protocol; PP, Percentage point; PPI, percentage point increase; pt, patient(s); OR, odds ratio; RR, relative risk ratio; SES, socioeconomic status(es); SHP, State Health Plan; ST, standard intervention; TP, tailored print; US, United States; VA, Veterans Affairs (US); vs., versus; W, women; wk(s), week(s); y, year(s).

a Group 4 women without available phone numbers included in denominator.

b Group 4x only women with available phone number included in denominator.

* If data were available in a report and the percentage point (PP) increase was not already reported, the PP increase was calculated and included in the Results column.
