# Supplementary material for: What implementation interventions increase cancer screening rates? a systematic review
Source: Implement Sci. 2011 Sep 29;6:111. doi: 10.1186/1748-5908-6-111 (PMC3197548; doi:10.1186/1748-5908-6-111)
Supplement: Additional file 9 — Study quality characteristics of included randomized controlled trials for group education and one-on-one education. Information on publication status, funding, randomization method, baseline, characteristics, blinding, statistical power, target sample size, follow-up period and intention to treat analysis are provided. [file 1748-5908-6-111-S9.DOC]

**Additional File 9. Study quality characteristics of included randomized controlled trials for Group Education and One-On-One Education**

| **Study** | **Publication status** | **Funding** | **Randomization method** | **Baseline characteristics** | **Blinding** | **Statistical Power** | **Achievement of Target Sample Size** | **Follow-up** | **Intention-to-Treat (ITT) analysis** |
| --- | --- | --- | --- | --- | --- | --- | --- | --- | --- |
| ***Group Education: Breast Cancer*** | | | | | | | | | |
| **Clustered** | | | | | | | | | |
| Mishra et al.,  2007 [53] | Full publication | CBCRP/UC & NCMHHD | NR  (Churches stratified on denomination and congregation  size; randomly assigned to either intervention or  control) | Clustering group: NR  Patient groups: Balanced | Interviewers blinded to study group status | Sample n=300 per study arm would have ›80% power  to detect 15% difference in primary outcome | Yes | At approx. 8 mos | 96% of original randomized sample analyzed: 33 pts lost to attrition; analysis group:14 not located, 11 deceased, 8 moved |
| Allen et al., 2008 [55] | Full publication | NCI | NR  (Work-site cluster stratified by age; randomly assigned to either intervention or  control) | Clustering group: control vs. intervention balanced  Patient groups:  Stratum 1 (<52 yrs) had higher incomes, education and more likely to be married compared to stratum 2 (>52 yrs) | NR | NR | NR | At 24 mos | 98% of original randomized sample analyzed |
| **Non-Clustered** | | | | | | | | | |
| Nguyen et al., 2009 [54] | Full publication | CDC, NCI & AANCART | Random drawing of participant names (odd selections assigned to one group, even selections to the other) | Balanced | NR | Target sample of 495 pts in each group to detect 15% intervention effect, a 5% comparison effect size and a net effect size of 10%  (alpha=0.05, beta=0.20) | Yes | At 3 & 6 mos | Analysis of 99% of randomized pts; 11 pts lost to follow-up |
| ***Group Education: Cervical Cancer*** | | | | | | | | | |
| **Clustered** | | | | | | | | | |
| Mishra et al., 2009 [56] | Full publication | NCI & NCMHHD | Randomization of the study locations (Eastern & Western districts) and study sites (churches) | Unbalanced for age and marital status; control group pts more likely to be younger and single | Interviewers blinded to study group status | NR | NR | At 6 mos | NR |
| ***Group Education: Colorectal Cancer*** | | | | | | | | | |
| **Clustered** | | | | | | | | | |
| Braun et al.,  2005 [57] | Full publication | NCI | Randomized by coin toss | Balanced | NR | NR | NR | At 16 wks | 4 lost to f/u; not included in analysis |
| Blumenthal et al., 2010 [58] | Full publication | CDC, NCI & NCRR | Participants randomized by site (church, community/ senior centre, or clinic) | Insurance coverage unbalanced among the 3 groups | NR | underpowered | No | At 3 and 6 mos | Yes; unable to contact 112 participants; loss to f/u = 30.4% |
| ***One-on-One Education: Breast Cancer*** | | | | | | | | | |
| **Clustered** | | | | | | | | | |
| Paskett et al., 2006 [60] | Full publication | NCI/NIH | Stratified selection and blocked randomization by race and clinic | Balanced | Interviewers,  abstractors, and primary care physicians  blinded to intervention assignment | Sample size = 800 designed to achieve 80% power to detect difference of at least 10% between groups & of at least 20% between groups within each racial group | Yes | At 12-14 mos | Yes; 41 lost to f/u: 14 deceased, 16 moved, 11 unable to participate |
| Fernandez et al., 2009 [62] | Full publication | CDC & NCI | Matched pairs of communities randomly assigned to either group  Participants clustered by community | Balanced | NR | NR | NR | At 6 mos | Yes; 33.1% lost to f/u |
| **Non-clustered** | | | | | | | | | |
| Dietrich et al., 2007 [59] | Full publication | NCI | Stratified by clinic, then by age decade, then randomized: method NR | Balanced | NR | NR | NR | At 11 mos | Yes; 42 dropouts |
| Carney et al., 2005 [61] | Full publication | ACS & NCI | NR | Balanced | NR | NR | NR | At 12 & 27 mos | NR |
| ***One-on-One Education: Cervical Cancer*** | | | | | | | | | |
| **Clustered** | | | | | | | | | |
| Fernandez et al., 2009 [62] | *See One-on-One Education: Breast Cancer* |  |  |  |  |  |  |  |  |
| **Non-Clustered** | | | | | | | | | |
| Dietrich et al., 2007 [59] | See *One-on-One Education: Breast Cancer* |  |  |  |  |  |  |  |  |
| ***One-on-One Education: Colorectal Cancer*** | | | | | | | | | |
| **Clustered** | | | | | | | | | |
| Blumenthal et al., 2010 [58] | *See Group Education: Colorectal Cancer* |  |  |  |  |  |  |  |  |
| **Non-clustered** | | | | | | | | | |
| Turner et al.,  2008 [67] | Full publication | PMCBF | Randomized in blocks of 6 | Intervention group contained more minorities | Randomization not blinded | 70 patients per arm could detect 20% difference; alpha=0.05; beta=0.80; two-tailed test | No | Date of 1^st^ scheduled appointment | NR |
| Costanza et al., 2007 [64] | Full publication | CDC & NCI | NR | Balanced | NR | NR | NR | At 17 and 22 mos | NR |
| Dietrich et al., 2007 [59] | See *One-on-One Education: Breast Cancer* |  |  |  |  |  |  |  |  |
| Basch et al.,  2006 [63] | Full publication | NCI | Random permutations table;  individuals blocked as to gender and age, assigned randomly within blocks to groups | Balanced | Staff ascertaining outcomes blinded to intervention status | Necessary sample  sizes per group for power of 0.80 found to be 207 for men and 395 for women on basis of effect sizes, for 1-tailed test with p=.01 | No | At 6 mos | Yes; 26 pts lost to f/u: 12 could not be contacted, 14 refused to complete survey |
| Tu et al.,  2006 [68] | Full publication | NCI | Computerized random number  generator to assign to group | Balanced | Chart audit staff blinded to trial arm assignment | NR | NR | Within 6 mos | NR |
| Jandorf et al., 2005 [66] | Full publication | NCI | NR | Balanced | Physicians blinded; chart reviewer not blinded | NR | NR | At 2-3 wks, 3 mos, & 6 mos | NR |
| Lipkus et al.,  2005 [65] | Full publication | NCI | NR | Balanced | NR | NR | NR | At 3, 12, & 24 mos | Yes; lost to f/u NR |
| Stokamer et al., 2005 [69] | Full publication | NR | Sequentially numbered, sealed, opaque envelopes | Balanced | Providers, staff, & pts not blinded | 388 per group would be required to detect absolute difference of 10%; power of 80% and a two-tailed α=.05. | Yes | Within 6 mos | Yes; 326 did not return FOBT card |
| Simon et al.,  2010 [70] | Full publication | HPHCF | Patient allocated to either arm using a computerized random number generator | Balanced | Not blinded | NR | NR | Within 12 mos | Yes |

Notes: AANCART, Asian-American Network for Awareness, Research & Training; ACS, American Cancer Society; CBCRP/UC, California Breast Cancer Research Program, University of California; CDC, Centres for Disease Control and Prevention; f/u, follow-up; HPHCF, Harvard Pilgrim Health Care Foundation; ICC, intra-cluster coefficient; ITT, intention-to-treat; mos, months; NCI, National Cancer Institute; NCMHHD, National Center for Minority Health and Health Disparities; NCRR, National Centre for Research Resources; NIH, National Institutes of Health; NR, not reported; PMCBF, Pennsylvania Medical Center’s Bach Fund; pts, patients; vs., versus; wks, weeks.
