# Supplementary material for: What implementation interventions increase cancer screening rates? a systematic review
Source: Implement Sci. 2011 Sep 29;6:111. doi: 10.1186/1748-5908-6-111 (PMC3197548; doi:10.1186/1748-5908-6-111)
Supplement: Additional file 10 — Randomized controlled trial results: Group Education and One-On-One Education. Information on participant criteria, study group numbers, intervention descriptions, reporting, and results are provided. [file 1748-5908-6-111-S10.DOC]

**Additional File 10. Randomized controlled trial results: Group Education and One-On-One Education.**

| **Article**  **(References)** | **Population**  **Description** | **Study Group**  **Numbers** | **Grouping & Description** | **Reporting** | **Results*** |
| --- | --- | --- | --- | --- | --- |
| ***Group Education: Breast Cancer*** | | | | | |
| **Clustered** | | | | | |
| Mishra et al.,  2007 [53]  US – Los Angles & Orange County, CA | ≥ 42 y  Non-adherent 24 mos prior  American-Samoan | 61 churches  Control 29  Intervn 32  809 pts randomized  776 analyzed  Control 385  Intervn 391 | Unit of randomization: Church  All participants  Screening interview & pretest & posttest (at 8 mo) surveys  $US5.00 at survey completion  Control group: Usual care  Intervention group  English and Samoan interactive group discussion sessions  supplemented by educational booklets & skill building & behavioural  exercises | Self-report | Reported mammogram use not significantly different between groups:  Rate OR (95% CI) p PPI  I 47% .26 (0.74-2.14) 0.390 8.0  C 39%  Most likely to obtain mam in test I vs. C:  Women who heard of but not had mam: 52% - 36% = 16.0 PP increase; OR=1.99 95% CI, 1.03, 3.85; p=.04  Women with previous mam: 60% - 53% = 7.0 PP increase |
| Allen et al.,  2008 [55]  US – Massachusetts | Stratum 1 40-51y Stratum 2 ≥52y Union representation <90min to clinic White 85% | 26 work sites  1475 Patients  Stratum 1  Control 377  Intervn 385  Stratum 2  Control 350  Intervn 363 | Unit of randomization: Work sites  All participants  Baseline & follow-up surveys  Control group: Usual care  Intervention group  Peer health advisors conducted small group education sessions and if necessary one-to-one out reach among coworkers in the form of instrumental, emotional and informational support for mammography screening | Self-report | Reported mammogram use not significantly different between groups: Stratum 1:  Rate OR (95% CI) p PPI  I 87% 0.94(0.58-1.55) 0.82 -1.0  C 88%  OR_adj_= 1.06; 95% CI (0.63-1.78);  (adjusted for association with social network measures)  Stratum 2:  Rate OR (95% CI) p PPI  I 92% 0.74(0.34-1.62) 0.82 -2.0  C 94%  OR_adj_= 0.72; 95% CI (0.35-1.47);  Recent baseline mammogram strong predictor of follow-up screening in comparison to those who had not; Stratum 1: 17 times greater Stratum 2: 37 times greater  Stratum 1 women predictor for mammography screening; -encouragement by family; OR 2.20  -subjective norms; OR 1.18  Stratum 2 women predictor for mammography screening; -perception that screening was normative; OR 1.46 |
| **Non-clustered** | | | | | |
| Ngyuen et al., 2009 [54] US – Santa Clara County, CA | ≥40 y Female  Vietnamese ethnicity Accrual: 2004 - 2007 | 1089 (initially 1100)  ME 546  ME+LHWO 543 | All participants  LHW conducted pre- and post- intervention telephone survey. Participants received a $30 incentive upon enrolment.  Media Education (ME): Comparison group  Community–wide breast cancer campaign included 6 Vietnamese TV and radio ads, 13 newspaper ads and 6 newspaper articles. 45000 bilingual booklets, 8500 silk rose reminder cards and 7000 reminder calendars were distributed at community events, churches and flea markets    ME + LHW Outreach intervention  In addiction to media campaign exposure, participants attended outreach sessions. LHW cancer presentation and f/u Q&A session. Within 1-2 mos, LHW calls to explain how to access screening and help scheduling appts. Reminders at 3 & 4 mos. | Self-report | Intervention effect size significantly greater than the comparison effect size for mammography screening within the past 2 years  % Screened   Pre- Post- %Diff◊ p-value C 74.0% 75.6% +2.4% 0.37 **I** 64.7% 82.1% +16.2% <0.001  I 16.2% - C 2.4% = +14.2 PPI◊  OR_adj_ 3.21; 95%CI (1.92-5.36)  (adjusted for sociodemographics, knowledge and media exposure) |
| ***Group Education: Cervical Cancer*** | | | | | |
| **Clustered** | | | | | |
| Mishra et al.,  2009 [56]  US – Los Angles & Orange County, CA | ≥20 y  Non-adherent 24 mos prior No history of cervical cancer or hysterectomy  American-Samoan | 26 churches  Control 13  Intervn 13  398 patients  Control 197  Intervn 201 | Unit of randomization: Church  All participants  $US5.00 for pre-test & post-test survey  Control group: Usual care  Intervention group  English and Samoan culturally tailored interactive group discussion sessions supplemented by educational booklets, skill building & behavioural exercises. Participants given $US5.00 for each session  Cluster trial did not adjust for design effect; unit of analysis error | Self-report | Self-reported Pap smear use significantly different between groups, favouring an intervention effect:  Rate OR_adj_(95% CI) p PPI  I 61.7% 2.0(1.3-3.2) <0.01 23.4  C 38.3%  Cervical screening higher among women >40 y, household income of $10,000 or above, and having concerns about the screening exam |
| ***Group Education: Colorectal Cancer*** | | | | | |
| **Clustered** | | | | | |
| Blumenthal et al.,  2009 [58]  US – Atlanta, GA | >49 y  African American  No history of CRC  Due for CRC screening  Accrual: Jan 2003 - April 2005 | 65 sites (Any test - FOBT, FS or Col)  369 Patients  Group 1 88  Group 2 98  Group 3 99  Group 4 84 | Unit of randomization: site  All participants  Given information at introductory meeting about CRC guidelines, referrals, insurance, screening sites and transport services. Completed pre-test survey. Offered supermarket gift cards/key rings  Group 1: Control group – usual care  Group 2: One-on-One Education  Health educator met participants for three 45 min sessions to review educational materials on CRC  Group 3: Group Education Health educator met participants in groups of 4-14 for 4 sessions to review CRC educational info  Group 4: Financial Support Offered financial reimbursement for up to $500 for out-of-pocket expenses incurred for screening  Unclear whether this cluster trial adjusted for design effect. | Medical records | Intervention groups showed greater adherence than control: Group %screened PPI  4 16.7 4.2  3 22.2 9.7  2 17.4 4.9  1 12.5  Compared with controls, the group education cohort nearly doubled the rate at which participants were screened. The other two interventions show promise, however in comparison, their level of efficacy did not reach statistical significance. |
| Braun et al.,  2005 [57]  US – Hawaii | ≥ 50 y  Female 72%  Native 90%  Urban/rural  Accrual: 2002-2003 | Civic Clubs 16  Participants 121  (FOBT)  Control  8 clubs  52 pts  Experimental  8 clubs  69 pts | Unit of randomization: Hawaiian civic clubs  Control arm  Culturally relevant targeted educational slide presentation to group  by non-Hawaiian nurse + CRC brochure + free FOBT kit with basic  instructions given by nurse  Telephone reminder if FOBT not completed in 1 mo  Experimental arm  Culturally relevant targeted educational presentation by Native Hawaiian physician + personal story of Native Hawaiian CRC survivor + free FOBT kit with model instructions from physician + challenge to share educational materials with family members  Multiple barrier assessment telephone calls 4-16 wks post-presentation to those not completing FOBT test | Laboratory results | 1 (59%) of experimental arm & 36 (69%) of control already up-to-date for screening  13 participants (11%) screened for 1^st^ time as result of study  Control = 8  Experimental = 5  31 participants (26%) remained unscreened  Conclusion: culturally relevant educational group presentation by Native Hawaiian health professional less effective than culturally targeted program by non-Native Hawaiian health professional at increasing rates in highly compliant individuals |
| **One-on-One Education: Breast Cancer** | | | | | |
| **Clustered** | | | | | |
| Paskett et al.,  2006 [60]  US – Robeson Co. NC | > 40 y  Non-adherent 1 y prior  Native 42%  White 25%  African-American 33%  Rural  Low SES 83%  Accrual: 1998 - 2002 | 851 participants  Compar 418  Intervn 433  Blocked randomization by race & clinic | All participants  $US10 grocery gift certificates for baseline & f/u survey completion  Comparison group  At 6 mo sent letter and brochure on need for regular *cervical* screening  At 3 mo post-follow-up survey sent mam invitation letter & information brochure  Intervention group  Culturally & personally tailored lay health advisor (LHA) interactive,  face-to-face, intensive educational intervention—3 in-person visits,  with educational materials; small gift at last visit; follow-up phone  calls and mailings after each visit  Unclear whether this cluster trial adjusted for design effect. | Medical records & self-report | Intervention showed statistically significant association with mammography receipt, including by race:  Rate RR (95% CI) p PPI  I 42.5% 1.56(1.29,1.87) <.001 15.2  C 27.3%  African 1.54(1.11,2.14) .008  Native 1.58(1.18,2.13) .002  Whites 1.54(1.05,2.25) .024 |
| Fernandez et al.,  2009 [62]  US – NM, TX & CA | ≥50 y  Hispanic  Farm-worker status  Low literacy No prior/ current cancer diagnosis  Non-adherent to breast screening  Accrual: 2004 | 464 participants  Control 257  Intervn 207 | Unit of randomization: Community  All participants  $US20 incentive upon completion of each interview; baseline and follow-up  Control group: Usual Care  Intervention group  A LHW set up a 1-on-1 session 2 mos after enrolment to present and discuss breast cancer screening using Cultivando la Salud materials. Within 2 wks participants contacted again by LHW to provide any further assistance | Medical records & self-report | Mammography screening completion higher among women in the intervention group for the subset of women who completed the f/u:  Rate PPI p  I 40.8% +10.9 <.05  C 29.9%  In an ITT analysis including all women within the cohort, differences between I vs. C groups remained, but were not significant  Rate PPI p I 25.6% +5.0 >.05  C 20.6% |
| **Non-Clustered** | | | | | |
| Dietrich et al.,  2007 [59]  US – NYC  **Breast, cervical, & colorectal screening** | 40-69 y  English & Hispanic  Urban  Low-moderate SES | 1316  (all tests)  AMOP 653  PCM 663 | Comparison: AMOP intervention  Established screening telephone outreach program + Educational material mailed about breast, cervical, & colorectal screening + 2-sentence telephone call about *cervical* & *colorectal* screening  PCM intervention  ACOP + AMOP expanded to include detailed telephoned scripted barrier assessment & assistance + scheduling assistance + appt reminders for all 3 screening tests  Financial incentive ($US25.00 gift certificate) upon mammogram completion | Administrative data | No significant mammogram uptake difference between the 2 groups:  PCM 19% - AMOP 17% = +2.0 PPI  OR_adj_=1.16; 95%CI(0.86-1.57); p=.3  At follow-up, women ≥50 years in PCM group almost twice as much up-to-date for all 3 tests (59 of 317, 18.6%) as in AMOP (33 of 309, 10.7%):  OR 2.00; 95% CI, 1.24-3.22; p<.01  Drawbacks:  No control group  PCM intervention modified in ways that actually reduced its impact |
| Carney et al.,  2005 [61]  US - NH | ≥ 40 y  Non-adherent 24 mos  Accrual: 1999-2000 | 258  Mailed 132  Telephone 126 | Mailed intervention – comparison group  Two mailings one year apart of breast & mammography information  brochures  Telephone counselling intervention  Two educational telephone calls with counselling support to help  overcome identified barriers | NH mammography registry records | Telephone intervention resulted in significantly (p=.04) more mammograms than the mailed intervention between first & second intervention contact:  60.3% - 47.7% = 12.6 PP increase  After second contact, telephone group rate dropped significantly from 60.3% to 41.3% (p=.002) and was similar to mailed group (41.3% vs. 34.8%, p=.29) |
| **One-on-One Education: Cervical Cancer** | | | | | |
| **Clustered** | | | | | |
| Fernandez et al.,  2009 [62]  US – NM, TX & CA | For details, see One-on-One Education: Breast Cancer | 243 participants  Control 111  Intervn 132 | For details, see One-on-One Education: Breast Cancer | Medical records & self-report | Cervical screening completion higher among women in the intervention group:  Rate PPI p  I 39.5% +15.9 <.05  C 23.6%  In an ITT analysis, the differences between I vs. C groups remained, but were not significant Rate PPI p I 24.2% +5.3 >.05  C 18.9% |
| **Non-clustered** | | | | | |
| Dietrich et al.,  2007 [59]  US – NYC  **Breast, cervical, & colorectal screening** | For details, see One-on-One Education: Breast Cancer | 1316  AMOP 653  PCM 663 | For details, see One-on-One Education: Breast Cancer | Administrative data | No significant difference between the 2 groups for cervical screening increase:  PCM 12% - AMOP 11% = 1.0 PPI,  OR_adj_ 1.18 (0.82, 1.70), p=.38  At follow-up, women ≥50 years in PCM group almost twice as much up-to-date for all 3 tests (59 of 317, 18.6%) as in AMOP (33 of 309, 10.7%);  OR 2.00; 95% CI, (1.24-3.22); p<.01  Drawbacks:  No control group  PCM intervention modified in ways that actually reduced its impact |
| ***One-on-One Education: Colorectal Cancer*** | | | | | |
| **Clustered** | | | | | |
| Blumenthal et al.,  2009 [58]  US – Atlanta, GA | For details see Group Education: Colorectal Cancer | 65 sites (Any test FOBT, FS or Col)  369 Participants  Group 1 88  Group 2 98  Group 3 99  Group 4 84 | For details see Group Education: Colorectal Cancer  Group 1: Control group – usual care Group 2: One-on-One Education  Group 3: Group Education Group 4: Financial Support  Unclear whether this cluster trial adjusted for design effect. | Medical records | Intervention groups showed greater adherence than control:  Group %screened PPI  4 16.7 4.2  3 22.2 9.7  2 17.4 4.9  1 12.5  Compared with controls, the group education cohort nearly doubled the rate at which participants were screened. The other 2 interventions show promise, however in comparison, their level of efficiency did not reach statistical significance. |
| **Non-clustered** | | | | | |
| Turner et al.,  2008 [67]  US – Burlington VT | ≥ 50 y  Scheduled for 1^st^ Col + poor attendance=<75% for primary care scheduled office visits since 2002  Female 69%  White 34%  Black 62%  Urban  Accrual: 2005-2006 | 136  (Col)  Brochure 66  Coach 70 | All participants Movie ticket coupon after recruitment  Intervention groups  (require support to ensure attendance)  Brochure group: Usual care + 2 educational information pt-oriented brochures—ACS & CDCP—about screening, especially Col  Peer coach support group: Usual care + scripted motivational/educational telephone interviews + barrier discussion within 2 wks of appt from peer coach | Medical records | Intervention group:  Peer coach group attendance 11% higher than Brochure group (p=.18)  OR_adj_=2.14; 95% CI (0.99-4.63); p=.05 |
| Costanza et al.,  2007 [64]  US – Massachusetts & Connecticut | 50-75 y  No Col prior 10 y  White 92%  English-speaking  Low/Mid/High SES  Medical coverage for CRC screening  Accrual: 2001-2004 | 2448 audited  (2806 at baseline)  (any test)  Control 1187  Intervn 1261 | All participants  Mailed baseline survey  Letter indicating personal physician recommended CRC screening but not specific type of test  Control group: Usual care  Intervention group  Step 1: 2 mo post-survey— 3-section educational print brochure on  CRC and screening  Step 2: 3 mo post-brochure— computer-scripted telephone  counselling call from trained counsellors—educational, motivational,  & barriers counselling | Chart audit | No overall difference in screening rates between intervention (25%) and control (24%) groups (less than half intervention group received counselling)  In subsequent subgroup analysis, highly significant screening uptake of any CRC test for counselled intervention subgroup (26%) vs. not-counselled intervention subgroup (15%) and control group (19%) (p.0001)  I subgroup 26% - C 19% = +7.0 PPI |
| Dietrich et al., 2007 [59]  US – NYC  **Breast, cervical, & colorectal screening** | ≥ 50 y  English & Hispanic  Urban  Accrual: 2005 | 626  (any test)  AMOP 309  PCM 317 | For details, see Client Reminders: Breast Cancer | Administrative data | PCM women more up-to-date for CRC screening after intervention vs. AMOP women:  PCM 32% - AMOP 25% = 7.0 PPI  OR_adj_=1.69; 95% CI (1.03-2.77);p=.04  At follow-up, women ≥50 years in PCM group almost twice as much up-to-date for all 3 tests (18.6%) vs. AMOP (10.7%):  OR=2.00; 95% CI (1.24-3.22); p<.01 |
| Basch et al.,  2006 [63]  US – NYC | >52 y  No screening in prior 2y  Female 70%  Black 68%  Low/middle SES  Accrual: 2000-2003 | 456  (any test - FOBT, FS, & Col)  Phone 226  Print 230 | Control group  Welcome letter + CDC brochure about CRC & screening with message to talk to physician & obtain screening  Intervention group  Tailored semi-structured telephone CRC education—median time 23.5 minutes & up to 5 calls—also addressing any barriers & with objective of verbal commitment to obtain screening  All participants  6-month follow-up telephone call to determine screening uptake | Medical claims & records  Self-report | Screening rates 4.4 times higher for intervention group than control group  RR = 4.4; 95% CI (2.6, 7.7)  I 27.0% - C 6.1% = +20.9 PP increase  7/226 in intervention group had only rectal examination + single stool test in physician’s office  Intervention group screening (n=61)  FOBT 29  Col 29  FS 2  FOBT + Col 1  Control group screening (n=14)  Col 13  FOBT + FS 1 |
| Tu et al.,  2006 [69]  US – Seattle WA | 50 – 78 y  Non-adherent to any CRC screening 1 y prior  Chinese, Cantonese,  Mandarin and/or English speaking  Female 63%  Low SES  Accrual: 2003-2004 | 210  (FOBT)  Control 105  Intervn 105 | Control group  Usual care— FOBT ordered by primary care provider🡪 kit with instructions  Intervention group  Health education by trilingual Chinese-American health educator about CRC, screening, population CRC risks & addressing barriers + Cantonese/Mandarin motivational video & pamphlet, CRC information pamphlet, & FOBT instructions | Medical records | Intervention effect size for FOBT screening completed = 42 percentage points  I 69.5% - C 27.6% = 41.9 PPI  Crude OR for FOBT screening within 6 mos follow-up:  OR=5.98; 95% CI (3.29, 10.85)  Adjusted for age, at 6 mos:  OR_adj_=5.91; 95% CI (3.25, 10.75)  Adjusted for all covariates, at 6 mos:  OR_adj=_6.38; 95% CI (3.44-11.85) |
| Jandorf et al.,  2005 [66]  US – East Harlem, NYC | ≥ 50 y  Non-adherent at least 1 y prior for FOBT & FS  Female 74%  Hispanic 82%  English 46%  Low SES <$US10,000  Accrual: 2002 | 78  (FOBT & FS)  PN- 40  PN+ 38 | Initial sociodemographic questionnaire  PN- group: no patient navigation (PN) services  PN+ group  Education & help with completing screening process provided by culturally equivalent trained patient navigator  PN telephoned subject 2-3 wks after initial contact to begin education and navigation services🡪 over 6 mo assessed & addressed information needs & any barriers; assisted with appt scheduling; telephoned to ensure appts kept; explain & answer questions on FOBT & FS procedure | Medical records | No significant differences noted for FOBT completion a 3-wk chart review  Non-significant differences at 3 mo review  FOBT: I 42.1% – C 25.0%= 17.1 PP increase; p=.086  FS: I 15.8% – C 5.0% = 10.8 PP increase; p=.115  Significant difference at 6 mo review for FS (p=.019):  I 23.7% – C 5.0% = 18.7 PPI |
| Lipkus et al.,  2005 [65]  US – New Jersey, NY | 50 – 75 y  Never had FS or Col  No FOBT within 15 mo prior  Caucasian  Carpenters  Male 99-100% | 860  (any tests - FOBT, FS, & Col)  N1 216  N2 212  T1 218  T2 214 | All participants:  Baseline, 3-mo, 1-y, & 2-y post-baseline telephone interview  Non-tailored Basic Information Group (N1)  4-page brochure with information on 3 basic CRC risk factors,  colorectal function, CRC lifetime risk, & 3 screening types (FOBT,  SF, & Col)  Non-tailored Comprehensive Information Group (N2)  N1 brochure + comprehensive information on lifestyle &  occupational risk factors  Tailored Basic Information Group (T1)  N1 information + information on own personal CRC risk factors (3  basic risk factors) + telephone counselling call 2 wk later to discuss  basic personal risk factors & need for screening  Tailored Comprehensive Info Group (T2)  N2 print material + tailored section on personal lifestyle and  occupation CRC risk factors + telephone counselling call to discuss  comprehensive & detailed specific personal risk factors (including  any new findings) | Medical claims data & Self-report | Overall, initial, yearly, and repeat screening rates not different between groups  Year 1 screening rate higher in T2 group than other 3 (p<.05)  Year 3 screening rate higher in TI group than other 3 (p<.05) |
| Stokamer et al.,  2005 [69]  US – NYC | ≥ 50 y  FOBT ordered by health care provider  Male 96%  White 47%  Black 35%  Hispanic 16%  ≥1 prior FOBT 60%  Accrual: 2002 | 788  (FOBT)  Standard 392  Intensive 396 | All participants:  Baseline data collection  Instructed to return completed FOBT cards within 2 wks  Standard education group  FOBT card (kit) with enlarged versions of the standard instructions for collecting specimen & prepare cards; pt to call clinic if any questions  Intensive education group  10-15 min educational session with primary care nurse on CRC  screening & FOBT—instruction on proper collection, card  preparation, how test works, what results means + 2-page handout  + FOBT kit—nurse answered any questions & pt to call clinic if any  additional questions | Returned card | FOBT card return significantly higher in intensive education group than in standard education  I 65.9% - C 51.3% = +14.6 PP increase p<.001  No return rate difference by gender or race  Return rate higher for pt with previous FOBT history (p=.002)  Median return time  Intensive education group 36 d  Standard education group 143 d  (P<.001 by log-rank test) |
| Simon et al.,  2010 [70]  US – Maine, New Hampshire, Massachusetts | 50-64 y  Health Plan members Non-adherent to CRC screening  Female 52.8%  Whit 85.9%  Black 4.6% | 20938  (initially 80000) (any tests)  Control 10506  Intervn 10432 | Control group: Usual Care  Intervention group  A single ATO call targeted knowledge deficits, addressed attitudes, provided positive encouragement for screening and assessed risk factors for CRC | Health plan membership records | No significant difference in obtaining CRC screening between intervention and control groups after adjusting for covariates  Rate OR_adj_(95% CI) p PPI  I 30.6%  1.01(0.94-1.07) .76 +0.2  C 30.4%  A small intervention effect on the adjusted OR of receiving a colonoscopy OR_adj_ 1.08; 95% CI (1.00-1.16) |

NOTES: ACOP, Affinity’s Clinical Outreach Program (US); ACS, American Cancer Society; adj, adjusted; ads, advertisements; AMOP, Affinity’s Mammography Outreach Program (US); appt, appointment; ATO, automated telephone outreach; C, control group; CDCP, Centers for Disease Control and Prevention; CI, confidence interval; Col, colonoscopy; Compar, comparison group; Compl, compliance; CRC, colorectal cancer; Diff, Difference; FOBT, fecal occult blood test; FS, flexible sigmoidoscopy; f/u, follow-up; I or Intervn, intervention group; ITT, intention-to-treat; mam, mammogram(s)/phy; LHW, lay health worker; M, men; min(s), minute(s); mo, month(s); NC, North Carolina; NCI CIS, NH, New Hampshire; NYC, New York City; OR, odds ratio; PCM, Prevention Care Management (US); PP. Percentage point; PPI, percentage point increase; pt, patient(s); Q&A, question & answer; RR, relative risk ratio; SES, socioeconomic status(es); SI, standard intervention; ST, standard intervention; TI, tailored intervention; TIP, tailored intervention + telephone reminder; TV, television; US, United States; vs., versus; W, women; wk(s), week(s); y, year(s).

* If data were available in a report and the percentage point (PP) increase was not already reported, the PP increase was calculated and included in the Results column.

◊ Calculation error in publication; numbers seen are as reported
