# Supplementary material for: What implementation interventions increase cancer screening rates? a systematic review
Source: Implement Sci. 2011 Sep 29;6:111. doi: 10.1186/1748-5908-6-111 (PMC3197548; doi:10.1186/1748-5908-6-111)
Supplement: Additional file 11 — Study quality characteristics of included randomized controlled trials for reducing structural barriers and out-of-pocket expenses. Information on publication status, funding, randomization method, baseline, characteristics, blinding, statistical power, target sample size, follow-up period and intention to treat analysis are provided. [file 1748-5908-6-111-S11.DOC]

**Additional File 11. Study quality characteristics of included randomized controlled trials: Reducing Structural Barriers and Out-Of Pocket Expenses.**

| **Study** | **Publication status** | **Funding** | **Randomization method** | **Baseline characteristics** | | **Blinding** | **Statistical Power** | **Achievement of Target Sample Size** | **Follow-up** | **Intention-to-Treat (ITT) analysis** |
| --- | --- | --- | --- | --- | --- | --- | --- | --- | --- | --- |
| ***Reducing Structural Barriers: Breast Cancer*** | | | | | | | | | | |
| **Non-clustered** | | | | | | | | | | |
| Nguyen et al., 2009 [54] | Full publication | CDC, NCI & AANCART | Random drawing of participant names (odd selections assigned to one group, even selections to the other) | Balanced | | NR | Target sample of 495 pts in each group to detect 15% intervention effect, a 5% comparison effect size and a net effect size of 10%  (alpha=0.05, beta=0.20) | Yes | At 3 & 6 mos | Analysis of 99% of randomized pts; 11 pts lost to follow-up |
| Russell et al., 2010 [36] | Full publication | NIH/NCI & Indiana University School of Nursing/ CEQLCI | Stratified random assignment into 3 age groups using a computer generated list | Low-dose comparison group more likely to have insurance | | Data collectors blinded; assistants and participants  not blinded | NR | Yes | At 6 mos | Yes |
| ***Reducing Structural Barriers: Cervical Cancer*** | | | | | | | | | | |
| **Non-clustered** | | | | | | | | | | |
| Oscarsson et al., 2008 [71] | Full publication | SRC & HRC | Computerized random assignment | NR | NR | | NR | NR | NR | Analysis incld all randomized pts; 230 lost from intervention group: 65 ineligible, 80 excld, 122  declined participation, & 13 refused smear |
| ***Reducing Structural Barriers: Colorectal Cancer*** | | | | | | | | | | |
| **Non-clustered** | | | | | | | | | | |
| Percac-Lima et al. 2008 [72] | Full publication | MGH, Jane’s trust, MCPCRN & MGPCPBRN | 1:2 ratio of patient randomization into intervention and control groups | Balanced | | NR | Target sample size of 408 pts in intervention group to detect 10% difference using a power of 80% with a 0.05 two-sided significance level | Yes | At 9 mos | NR |
| ***Reducing Out-of-Pocket Costs: Breast Cancer*** | | | | | | | | | | |
| **Non-clustered** | | | | | | | | | | |
| Slater et al.,  2005 [73] | Full publication | NCI/MLF | Cases split into the low and high mammography rate strata; computer- generated random numbers assigned; cases grouped by number values | Balanced | | Matching& adjudication process staff blind to intervention assignment; calling staff not blinded | ≥90% power to detect effects of 1% in absolute size while controlling overall α level at <0.05 | Yes | At 13 mos | Analysis included all randomized participants |
| ***Reducing Out-of-Pocket Costs: Colorectal Cancer*** | | | | | | | | | | |
| **Clustered** | | | | | | | | | | |
| Blumenthal et al., 2010 [58] | Full publication | CDC, NCI & NCRR | Participants randomized by site (church, community/senior centre, or clinic) | Insurance coverage unbalanced among the 3 groups | | NR | underpowered | No | At 3 & 6 mos | Yes; unable to contact 112 participants; loss to f/u = 30.4% |

Notes: AANCART, Asian-American Network for Awareness, Research & Training; CDC, Centres for Disease Control and Prevention; CEQLCI, Center for Enhancing Quality of Life in Chronic Illness; excld, excluded; f/u, follow-up; incld, included; ITT, intention-to-treat; MCPCRN, Massachusetts Cancer Prevention Community Research Network; MGH, Massachusetts General Hospital; MGPCPBRN, Massachusetts General Primary Care Practice-Based Research Network; MLF, Minnesota Lynx Foundation; mos, months; NCI, National Cancer Institute; NCRR, National Centre for Research Resources; NIH, National Institutes of Health; NR, not reported; pts, patients. SRC, Swedish Research Council
