# Supplementary material for: What implementation interventions increase cancer screening rates? a systematic review
Source: Implement Sci. 2011 Sep 29;6:111. doi: 10.1186/1748-5908-6-111 (PMC3197548; doi:10.1186/1748-5908-6-111)
Supplement: Additional file 12 — Randomized controlled trial results: Reducing Structural Barriers and Out-of-Pocket Expenses. Information on participant criteria, study group numbers, intervention descriptions, reporting, and results are provided. [file 1748-5908-6-111-S12.DOC]

**Additional File 12. Randomized controlled trial results: Reducing Structural Barriers and Out-of-Pocket Expenses.**

| **Article**  **(References)** | **Population**  **Description** | **Study Group**  **Numbers** | **Grouping & Description** | **Reporting** | **Results*** |
| --- | --- | --- | --- | --- | --- |
| ***Reducing Structural Barriers*** | | | | | |
| **Breast Cancer** | | | | | |
| Ngyuen et al., 2009 [54] US – Santa Clara County, CA | ≥40 y Female  Vietnamese ethnicity Accrual: 2004 - 2007 | 1089 (initially 1100)  ME 546  ME+LHWO 543 | All participants  LHW conducted pre- and post- intervention telephone survey. Participants received a $30 incentive upon enrolment.  Media Education (ME): Comparison group  Community–wide breast cancer campaign included 6 Vietnamese TV and radio ads, 13 newspaper ads and 6 newspaper articles. 45000 bilingual booklets, 8500 silk rose reminder cards and 7000 reminder calendars were distributed at community events, churches and flea markets    ME + LHW Outreach intervention  Media campaign exposure & LHW presentation and f/u Q&A session. Within 1-2 mos, LHW explained how to access screening and helped scheduling appts. Reminders sent 2 & 3 mos later | Self-report | Intervention effect size significantly greater than the comparison effect size for mammography screening within the past 2 years  % Screened   Pre- Post- %Diff◊ p C 74.0% 75.6% +2.4% .37 **I** 64.7% 82.1% +16.2% <.001  I 16.2% - C 2.4% = +14.2 PP increase◊; p<.001  OR_adj_ 3.21; 95% CI (1.92-5.36)  (adjusted for sociodemographics, knowledge and media exposure) |
| Russell et al., 2010 [36] US – Indianapolis, IN | 40-75 y African-American  ≤250% federal poverty level No mammogram within last 15 mos No breast cancer history  Urban  Accrual: 2006 – 2008 | 181  Compar 90  Intervn 91 | All participants  Baseline and 6 mos survey  Given $US25 gift certificate to a local business  Low-Dose: Comparison group  Culturally appropriate pamphlet about breast cancer and screening recommendations from a LHA to schedule a mammography. Received 4 mailed postcards at monthly intervals regarding general nutrition information  Combined Intervention group  Tailored computer assessment at baseline + 4 monthly LHA sessions. LHA offered access enhancing services, including referral to low-cost mammograms, assistance with scheduling screening appointments and help for those requiring transportation. | Medical records and self-report | The combined intervention improved mammography screening rates in low income African-American women  I 50.6% - C 17.8% = +32.8 PPI OR= 4.7; 95% CI (2.4-9.4); p<0.0001 RR_adj_= 2.7; 95% CI (1.8-3.7); p<0.0001  (adjusted for employment status, disability, insurance, 1^st^ degree relatives and previous breast biopsies) |
| **Cervical Cancer** | | | | | |
| Oscarsson et al.,  2008 [71]  Sweden | 28-65 y  Non-adherent at least 5 y prior  Accrual: 2004 | 800  Control 400  Intervn 400 | Control group  Standard invitation (automatic every 3 y)  Intervention group  Invitation letters + telephone interviews + promotive efforts (arranging appts, taking smears off-hours, transportation help, etc.) | Government database records (Sympathy) | Significant increase in screening rates for intervention group vs. control:  I 29.5% - C 18.5% = +11.0 PP increase, p<.001  Intervention group dropped to n=120, with n=50 requesting help obtaining smear (friendly treatment n=23; special appt scheduling n=21) Promotive efforts:  Special appt times n=16  (afterhours n=7)  Free appts n=2  Alternative venues n=6  (near home n=2)  Transport n=2  Examiner preference n=10 |
| **Colorectal Cancer** | | | | | |
| Percac-Lima et al., 2008 [72] US – Chelsea, MA | 52-79 y Due for CRC screening Female 60% Latino >40% White 47% Black 5% Low SES Accrual: 2007 | 1223 (Any test)  2:1 randomized  Control 814  Intervn 409 | Control group: Usual Care  Intervention group  Navigator sent an introductory letter to the patient in their native language and educational materials. Navigator reviewed available methods, helped schedule appointments, gave patients reminders, reviewed & translated materials and organized transportation | Medical records | CRC screening rates twice as high in the intervention group versus the control  Rate PPI p  I 27.4 +15.6 <0.001  C 11.9%  Colonoscopy specific rates I 20.8% - C 9.6% = +11.2 PPI; p<0.001  In subsequent subgroup analysis, relatively larger intervention effect seen in females, older patients, non-Latinos, English speakers and those without private insurance |
| ***Reducing Out-of-Pocket Costs*** | | | | | |
| **Breast Cancer** | | | | | |
| Slater et al.,  2005 [73]  USA- Minnesota | 40-64 y  Low SES  Underinsured  Actual population by census-block sampling using commercial mailing list  Accrual: 2000-2001 | 145,467 eligible participants  C 94,201  Mail 25,633  Mail+ 25,633 | Pre-intervention letter to all Minnesota primary physicians informing them of study and use of SAGE state screening program  Control (C): no intervention  Intervention 1/Mail  2 different personally addressed mailers 1 mo apart—card about  free mammogram + toll-free number for information  Intervention 2/Mail Plus Incentive  2 different personally addressed mailers 1 mo apart—card about  free mammogram + toll-free number for information + $US10  monetary incentive offer if mammogram obtained in approximately  1 year  Toll-free numbers for both groups accessed telephone direct appointment scheduling system determining eligibility and if so, providing 3-way connection (toll-free staff/caller/clinic) with conveniently located clinic + follow-up call to ensure appt made if not done at first call + appointment confirmation letter | SAGE record database | Interventions increased screening significantly  Mail Plus Incentive significantly enhances intervention effectiveness  Intervention effect versus Control: Pooled estimates statistically significant  Rate% (95% CI) p-value  Mail 0.23 (0.09-0.37) <.005  Mail+ 0.75 (0.58-0.92) <.001  Mail+ statistically significant greater effect than Mail:  Rate=0.52 (0.32, 0.72) p<.001  Incentive Claims:  N Mail+ eligible for SAGE = 488  N Mail+ claimed incentive = 123 (25%) |
| **Colorectal Cancer** | | | | | |
| Blumenthal et al.,  2009 [58]  US – Atlanta, GA | >49 y  African-American  No history of CRC  Due for CRC screening  Accrual: 2003 - 2005 | 65 sites (Any test - FOBT, FS or Col)  369 Patients  Group 1 88  Group 2 84 Group 3 98  Group 4 99 | Unit of randomization: site  All participants  Info session about CRC guidelines, referrals, insurance, screening sites and transport services. Completed pre-test survey. Offered supermarket gift cards/key rings  Group 1: Control group – usual care  Group 2: One-on-One Education  Health educator met participants for three 45 min sessions to review educational materials on CRC  Group 3: Group Education Health educator met participants in groups of 4-14 for 4 sessions to review CRC educational info  Group 4: Financial Support Offered financial reimbursement for up to $US500 for out-of-pocket expenses incurred for screening  Unclear whether this cluster trial adjusted for design effect. | Medical records | Intervention groups showed greater adherence than control:  Group %screened PPI  4 16.7 4.2  3 22.2 9.7  2 17.4 4.9  1 12.5   Compared with controls, the group education cohort nearly doubled the rate at which participants were screened. The other 2 interventions show promise, however in comparison, their level of efficacy did not reach statistical significance. |

Abbreviations: adj, adjusted; ads, advertisements; appt(s), appointment(s); C, control group; compar, comparison; CI, confidence interval; Col, colonoscopy; CRC, colorectal cancer; Diff, difference; FOBT, fecal occult blood test; FS, flexible sigmoidoscopy; f/u, follow-up; I, intervention group; Intervn, intervention; ITT, intention to treat; LHA, lay health advisor; LHW, lay health worker; mo, month(s); N, number; OR, odds ratio; PP(I), Percentage Point (Increase); pt(s), patient(s); Q&A, question & answer; RR, relative risk ratio; SES, socioeconomic status(es); TV, television; y, year(s).

* If data were available in a report and the percentage point (PP) increase not reported, the PP increase was calculated and included in the Results column.
◊ Calculation error in publication; numbers seen are as reported
