# Supplementary material for: What implementation interventions increase cancer screening rates? a systematic review
Source: Implement Sci. 2011 Sep 29;6:111. doi: 10.1186/1748-5908-6-111 (PMC3197548; doi:10.1186/1748-5908-6-111)
Supplement: Additional file 13 — Study quality characteristics of included randomized controlled trials for interventions directed at providers. All studies are related to provider assessment/feedback since no trials were obtained for provider incentive interventions. Information on publication status, funding, randomization method, baseline, characteristics, blinding, statistical power, target sample size, follow-up period and intention to treat analysis are provided. [file 1748-5908-6-111-S13.DOC]

**Additional File 13. Study quality characteristics of included randomized controlled trials: Interventions Directed at Providers. (No trials were obtained for provider incentive interventions.)**

| **Study** | **Publication status** | **Funding** | **Randomization method** | **Baseline characteristics** | **Blinding** | **Statistical Power** | **Achievement of Target Sample Size** | **Follow-up** | **Intention-to-Treat (ITT) analysis** |
| --- | --- | --- | --- | --- | --- | --- | --- | --- | --- |
| **Interventions directed at health care providers** | | | | | | | | | |
| ***Provider Assessment and Feedback: Breast, Cervical, and Colorectal Cancers*** | | | | | | | | | |
| **Clustered** | | | | | | | | | |
| Ferreira et al., 2005 [74] | Full publication | NCI & DVA | Random allocation of 1 clinic to control arm & 1 to intervention arm | Balanced | NR | 80% power to detect screening rate = 40.8% for intervention; two-tailed test ; type I error rate = 5% | NR | Within 6 to 18 mos | NR |
| Aspy et al.,  2008 [75] | Full publication | CMMS | Physicians randomly assigned using an internet randomizer | Missing data thus reasonable comparisons could not be made | NR | NR | Yes | Within 3 to 15 mos | NR |
| **Non-clustered** | | | | | | | | | |
| Mold et al.,  2008 [76]  Breast and Colorectal Cancer | Full publication | AHRQ | Practices randomized in blocks of 4 using a series of coin tosses | Physician data collected; no comparisons made | Expert panel and research assistant blinded to practice assignment; Clinician partially blinded | Powered to have better than 80% chance to detect implementation rate twice as high in the intervention group | NR | At 6 mos | NR |
| ***Provider Assessment but no Feedback Component: Breast, Cervical, and Colorectal Cancers*** | | | | | | | | | |
| Dubey et al.,  2006 [77] | Full publication | PSI | Random number table used | More female pts & less comorbidity and more older experienced doctors in intervention arm | Abstractors blinded in pre-intervention period | 5% type I error, and 85% power—220 charts needed per group to detect mean difference=30%; alpha type I error=0.05 and 90% power—273 charts per group needed | Yes | At 5 mos | Yes |
| Walsh et al.,  2005 [79] | Full publication | ACS & UCSF | Block randomization of physicians, stratified by group size | Physicians: more of intervention group in academic setting  Patients: balanced | NR | Type I error=0.05; 80% power to detect effect differences | NR | At 1, 2, and 5 yrs, depending on group | NR |
| Michielutte et al., 2005 [35] | Full publication | NCI | NR | Balanced | NR | NR | NR | At 4 mos, 9 mos, & 12 mos | No; 11% attrition rate for complete f/u |
| Jensen et al., 2009 [23] | Full publication | NR | Block randomization at the physician level according to practice numbers | Data from practices collected; balanced | GPs and researchers not blinded | NR | NR | At 5, 8 & 11 mos | NR |
| Lane et al.,  2008 [78] | Full publication | NCI & AHCQR | Physicians clustered under health centre | Balanced | Chart abstractors blinded to health centre study condition | Detecting a treatment effect of 15% at 80% power, alpha = 0.05 (two-tailed) | Yes | At 12 mos | NR |
| ***Provider Incentives: Breast, Cervical, and Colorectal Cancers*** | | | | | | | | | |
| **Clustered** | | | | | | | | | |
| Federici et al., 2006 [80] | Full publication | NR | NR | NR for GPs; balanced for pts | NR | Power 90%, alpha .05, to detect RR=1.44; average cluster size = 55; resulting study size 130 cluster and 7150 subjects | Yes | After 45 days | NR |

Notes: AHCQR, Agency for Health Care Quality and Research; AHRQ, Agency for Health Research and Quality; CMMS, Centres for Medicare and Medicaid Services; DVA, Department of Veterans Affairs; GP(s), general practitioner(s); ITT, intention-to-treat; mos, months; NCI, National Cancer Institute; NR, not reported; PSI, Physicians’ Services Incorporated Foundation.
