# Supplementary material for: What implementation interventions increase cancer screening rates? a systematic review
Source: Implement Sci. 2011 Sep 29;6:111. doi: 10.1186/1748-5908-6-111 (PMC3197548; doi:10.1186/1748-5908-6-111)
Supplement: Additional file 14 — Randomized controlled trial results: Interventions Directed at Providers. All studies are related to provider assessment/feedback since no trials were obtained for provider incentive interventions. Information on participant criteria, study group numbers, intervention descriptions, reporting, and results are provided. [file 1748-5908-6-111-S14.DOC]

**Additional File 14. Randomized controlled trial results: Interventions Directed at Providers**

| **Article**  **(References)** | **Population**  **Description** | **Study Group**  **Numbers** | **Grouping & Description** | | | **Reporting** | **Results*** |
| --- | --- | --- | --- | --- | --- | --- | --- |
| ***Provider Assessment and Feedback*** | | | | | | | |
| **Breast, Cervical, and Colorectal Cancers** | | | | | | | |
| Ferreira et al.,  2005 [74]  US – Chicago  Colorectal screening | VA medical centre  Providers:  Physicians  Residents  Nurse  Practitioners  Accrual: 2001-2003 | 2 clinics:  Control:  Physician 3  Resident 49  NP 1  Pts 963  Intervn:  Physician 3  Resident 55  NP 3  Pts 1015 | 2 VA clinics randomized as control & intervention setting  Examined provider intervention & patient intervention (See Table 3c, Fitzgibbon et al for patient results for this study)  Control clinic: usual care  Intervention clinic  2-hr workshop on rationale/guidelines for CRC screening & effective communication with patients  1-hr feedback sessions every 4-6 mo to provide clinic and individual practitioner (confidential data) with test recommendation and patient adherence results; small group discussions; role playing | | | Electronic medical records | Intervention significantly increased colorectal screening recommendations (7% absolute increase) and completion rates (9% absolute increase).  Screening recommendations (%) (n=963):  C I  FOBT 2.8 6.3  FS/Col 44.4 19.2  FOBT/FS/Col 22.1 50.4  Any test 69.4 76.0 p=.02  Screening completion (%) (n=1,015):  C I  FOBT 14.3 22.6  FS/Col 15.3 12.2  FOBT/FS/Col 2.8 6.5  Any test 32.4 41.3 p=.003  I 41.3% – C 32.4% = +8.9 PPI |
| Aspy et al., 2008 [75] US – Oklahoma  Breast Screening | ≥50 y Clinic visit at least 12 months prior No breast cancer diagnosis | 16 practices  Control 8  Intervn 8 | Unit of randomization: practice  Control clinics  Intervention clinic   1. Practice audit results in comparison to network 2. Academic detailing of exemplar principles 3. Services of a facilitator; providing information and feedback to guide practice redesign activities 4. Information technology support if requested | | | Medical records | The intervention group significantly increased mammography rates % Screened   Pre- Post- %Diff C 40% 35% -5%  I 40% 52% +12% I 12% - C -5% = +17 PPI; p=0.015  Intervention group: 6/8 practices improved screening rates; 2/8 practices remained the same  Control group: 2/8 practices showed an improvement; 3/8 practices declined in referral rates |
| Mold et al., 2008 [76] US – Oklahoma  Breast Screening | 50-75 y Overdue for breast screening | NR | Control clinics  Feedback and benchmarking  Intervention group  Combination of feedback, benchmarking, academic detailing of evidence processes (standing orders, reminders and prevention clinics), practice facilitation and IT support. Facilitator assisted in developing forms, training staff, and auditing charts. | | | Medical records | In the sub-group analysis for mammography screening rates, intervention practices increased uptake in comparison to control groups %Screened   Pre- Post- %Diff p-value C 54% 61% +7% 0.26 I 33% 60% +27% 0.001  I 27% - C 7% = +20 PPI |
| Mold et al., 2008 [76] US – Oklahoma  Colorectal Screening | 50-75 y Overdue for CRC screening | NR | See breast cancer Provider Assessment and Feedback for grouping & description details | | | Medical records | Sub-group analysis for CRC screening rates, similar differences were noted pre- and post- intervention in both arms %Screened   Pre- Post- %Diff p-value C 30% 46% +16% 0.02  I 28% 44% +16% 0.001  No significant difference between groups on overall adherence  I 16% - C 16% = 0 PPI |
| *Assessment but no feedback component* | | | | | | | |
| Dubey et al.,  2006 [77]  Canada – Toronto  Breast, Cervical & Colorectal Screening | St Michael’s Hospital/ University of Toronto clinics  Involved 13 preventive health services | 4 clinics  Control 2  Physician 18  Intervn 2  Physician 20 | Control: usual care  Intervention  Prompt/reminder form: Patient gender-specific Preventive Care Checklist Forms© attached by clerical staff to charts | | | Medical charts | Rate of form utilization in intervention group = 84%  Statistically significant change for FOBT in favour of intervention:  Post-intervention  RR 95% CI  Mam 1.41 (0.76-2.61)  Pap 0.92 (0.83-1.01)  FOBT 6.69 (1.9-24.1) p<.05    B PI %Diff p  Mam  I 41.8 76.6 34.8 <0.001  C 57.5 50.0 -7.5 0.76  I 34.8% - C -7.5% = 42.3 PPI  Pap  I 73.6 84.7 11.1 0.02  C 86.2 88.3 2.1 0.72  I 11.1% – C 2.1% = 9.0 PPI  FOBT  I 13.4 50.6 37.2 <0.001  C 3.6 7.5 3.9 0.45  I 37.2% – C 3.9% = 33.3 PPI |
| Walsh et al.,  2005 [79]  US - San Francisco CA  Colorectal screening | Individual practitioner association  Community or academic setting  Accrual: 2000-2005 | 94 (any CRC test)  Control 44  Intervn 50 | All participants:  Initial educational seminar  Letters summarizing recent colorectal study results  Control: usual care  Intervention  Questionnaire  One-on-one academic detailing by physician ‘opinion leader’ to discuss CRC guidelines and individual barriers, define objectives | | | Claims data | Physician screening rates similar between intervention and control groups  Any CRC test: Increase in control vs. intervention group:  Change mean = 13.7% vs. 12.6%, p=.47  FOBT: Physician screening rates increased in control group vs. intervention group:  Change mean = 15.9% vs. 12.7%, p=.25 |
| Michielutte et al.,  2005 [35]  US – NC    Breast Screening  3-stage study of provider & patients | urban/rural | Practices 43  (Physicians 127)  Control 22  Intervn 21 | Stage 1 of study: Practice/physician component  Stage 2/3: Control group - no intervention  Intervention – physician education  Current information on mammography fact sheet, telephone follow-  up (on request) and mammography pamphlets | | | Medical harts | No significant difference in obtaining mammography between intervention and control groups:  **I**% C% %Diff  Total 18.6 19.4 -0.8 p=.702 |
| Jensen et al.,  2009 [23]  Denmark – Aarhus  Cervical Screening | 23-59 y  Overdue for a Pap smear test | 117129 Patients  Control 59183  Intervn 57946 | Unit of randomization: GP  All eligible women received an invitation to the screening program  Control Group: Usual care  Intervention Group  The GP’s received a visit from a facilitator to provide quality  enhancements to the cervical screening program and offered to  contact non-attenders by mailing out a special targeted letter  personally signed by the GP emphasizing reasons for screening | | | Medical database | The intervention effectively and consistently increased the proportion of women receiving cervical screening;  PPI 95% CI  3mos +0.70% 0.13%-1.28% 6mos +0.94% 0.21%-1.67%  9mos +1.97% 0.03%-3.91% trend test: p<0.036  Screening rates 1.17 (95%CI: 1.04-1.30) times higher for intervention group than control group  (adjusted for GPs characteristics and proportion of non-attenders) |
| Lane et al., 2008 [78] US – Long Island, NY  Colorectal Screening | ≥50 y No prior CRC diagnosis Due for CRC screening Female 63%  Non-white 78% | 8 health centre (any CRC test)  Control 4  Intervn 4 | Unit of randomization: health centre  Control group: no intervention  Intervention Group:  The GP’s received pre-intervention assessment of their practice. The  group physician education component explained common CRC  barriers, facilitation of risk communication as well as informed  shared decision making skills. A non-educational intervention  followed using SWOT analysis to increase practice efficiency | | | Medical records | Pre- to post-intervention changes in CRC screening significantly greater in intervention group  %Screened   Pre- Post- %Diff p-value C 37% 41% +4% 0.40 I 45% 61% +16% <0.001 I 16% - C 4% = +12 PPI  OR_adj_= 2.25; 95%CI (1.67-3.04); p<0.001 (adjusted for clustering pts within health centres and health centre and patient level covariates)    *CRC screening rates include referral/ dispensing/ completion |
| ***Provider Incentives*** | | | | | | | |
| **Breast, Cervical, and Colorectal Cancers** | | | | | | | |
| Federici et al.,  2006 [80]  Italy  Colorectal Screening | Guaiac & Immuno- chemical FOBT  Accrual: 2002-2003 | Usual Care  Hospital 13  Pts 3675  Intervention  GPs 130  Pts 3657 | | Hospital Group: no intervention  GP Provider Group Intervention  Economic incentive €1,000 ($CAN1.57, 2008 10 31) for participation, €10 for each patient screened |  | | GP Provider Group screening compliance rate for FOBT return higher than hospital rate:  I 50.3% - C 16.2% = +34.1 PPI  RR, 3.40; 95% CI (3.13-3.70) |

Notes: B, baseline; C, control; Col, colonoscopy; CRC, colorectal; Diff, difference; FOBT, fecal occult blood test; FS, flexible sigmoidoscopy; GP, general practitioner; hr, hour; I or Intervn, intervention; IT, information technology; Mam, mammography; mo(s), month(s); n, number; NP, Nurse practitioner; NR, not reported; OR, odds ratio; Pap, Papanicolau; PI, post-intervention; PP, percentage point; PPI, percentage point increase; pt(s) patient(s); RR, relative risk ratio; SWOT, Strengths, Weaknesses, Opportunities & Threats tool; US, United States; VA, Veterans Administration; y, year;

* If data were available in a report and the percentage point (PP) increase not reported, the PP increase was calculated and included in the Results column.
